# Physiological and transcriptome profiling revealed defense networks during *Cladosporium fulvum* and tomato interaction at the early stage

**DOI:** 10.3389/fpls.2022.1085395

**Published:** 2022-12-06

**Authors:** Rong Peng, Sheng Sun, Na Li, Lingjuan Kong, Zhifeng Chen, Peng Wang, Lurong Xu, Hehe Wang, Xueqing Geng

**Affiliations:** ^1^ College of Horticulture, Shanxi Agricultural University, Jinzhong, Shanxi, China; ^2^ School of Agriculture and Biology, Shanghai Jiao Tong University, Shanghai, China; ^3^ Vegetable Department, Shanghai Agricultural Technology Extension and Service Center, Shanghai, China; ^4^ College of Biology and Agricultural Technology, Zunyi Normal University, Zunyi, China; ^5^ Clemson University, Edisto Research and Education Center, Blackville, SC, United States

**Keywords:** *Cladosporium fulvum*, tomato, RNA-seq, plant hormones, defense responses, differentially expressed genes (DEGs)

## Abstract

Tomato leaf mold caused by *Cladosporium fulvum* (*C. fulvum*) is a serious fungal disease which results in huge yield losses in tomato cultivation worldwide. In our study, we discovered that ROS (reactive oxygen species) burst was triggered by *C. fulvum* treatment in tomato leaves. RNA-sequencing was used to identify differentially expressed genes (DEGs) induced by *C. fulvum* inoculation at the early stage of invasion in susceptible tomato plants. Gene ontology (GO) terms and Kyoto Encyclopedia of Genes and Genomes (KEGG) databases were used to annotate functions of DEGs in tomato plants. Based on our comparative analysis, DEGs related to plant-pathogen interaction pathway, plant hormone signal transduction pathway and the plant phenylpropanoid pathway were further analyzed. Our results discovered that a number of core defense genes against fungal invasion were induced and plant hormone signal transduction pathways were impacted by *C. fulvum* inoculation. Further, our results showed that SA (salicylic acid) and ABA (abscisic acid) contents were accumulated while JA (jasmonic acid) content decreased after *C. fulvum* inoculation in comparison with control, and quantitative real-time PCR to detect the relative expression of genes involved in SA, ABA and JA signaling pathway further confirmed our results. Together, results will contribute to understanding the mechanisms of *C. fulvum* and tomato interaction in future.

## Introduction

Tomato (*Solanum lycopersicum*) is one of the most economically valuable vegetable crops in the world (Krishna et al., 2016). Tomato leaf mold caused by the biotrophic pathogen *Cladosporium fulvum* (*C. fulvum*) is a fungal disease which affects the quality and yield of tomato production severely (Griffiths et al., 2018). In China, yield losses due to tomato leaf mold are typically in the range of 10-25% and may exceed 50% when the disease is severe (Wang et al., 2018). Symptoms of tomato leaf mold usually appear in the leaf adaxial surface with irregular yellowish spots and the leaf abaxial surface with white mold layer at the early onset stage. As the disease develops, the spots become yellow-brown, the leaves curled and withered (Iida et al., 2010). As a model system to investigate the mechanism of tomato and *C. fulvum* interaction, a total of ten *C. fulvum* effector proteins have been identified, including four avriulence effector proteins (Avr2, Avr4, Avr3E and Avr9) and six extracellular proteins (Ecp1, Ecp2, Ecp4, Ecp5, Ecp6, and Ecp7) (Van Kan et al., 1991; Van Den Ackerveken et al., 1993; Joosten et al., 1994; Lauge et al., 2000; Luderer et al., 2002; Westerink et al., 2004; Bolton et al., 2008). Race-specific resistance responses against *C. fulvum* follow the typical gene-for-gene hypothesis. Once the avriulence effector was recognized by the dominant *C. fulvum* (*Cf*) resistance genes, the host activates the immune response against the pathogen (Rivas, 2005). To date, at least 24 *Cf* resistance genes have been discovered and applied in resistance breeding of tomato (Jiang et al., 2022b).

Plant hormones play key regulation roles not only in plant growth and development processes but also in plant responses to a wide range of biotic and abiotic stresses (Benoit and Patrick, 2016). More and more evidence has demonstrated that multiple hormones have been involved in plant and pathogen interactions (Denance et al., 2013; Li et al., 2019a; Liu et al., 2021). In general, salicylic acid (SA) signaling regulates plant defense against biotrophic pathogens, while the jasmonic acid (JA)/ethylene (ET) pathway normally defends against necrotrophic pathogens and herbivorous insects (Jane, 2005; Bari and Jones Jonathan, 2009). It has been documented that SA- and JA/ET-mediated defense signaling pathways synergistically or antagonistically depending on different pathogens and plants interaction (Jia et al., 2013; Yang et al., 2015; Li et al., 2019a). Plants were treated with low concentrations of SA and JA resulting in synergistic expression of both the SA target gene *PR1* and the JA marker gene *PDF1.2*, but the antagonism effect was observed when plants were treated with higher concentrations of SA and JA resulting to the antagonistic expression of these genes (Mur et al., 2006). When tomato plants were infected by the pathogen *Alternaria alternata* f. sp. *Lycopersici*, SA, JA and ET-dependent pathways synergistically activated the defense pathway (Jia et al., 2013). And the antagonistic mechanism between SA and JA has been demonstrated in many gymnosperm and angiosperm species (Yang et al., 2015). Recently, the synergistic action of SA and JA pathways was found to enhance plant resistance to herbivores in tea plants (Jiao et al., 2022). Abscisic acid (ABA) plays the multifaceted role in disease resistance, a general pattern is that ABA plays a stimulatory role in plant defense during early stages of pathogen invasion (Ton et al., 2009).

Sequencing of RNAs (RNA-seq) has been widely applied in many biological analyses including host and pathogen interaction (Gao and Chen, 2018; Li et al., 2018; Gao and Chen, 2021; Zhang et al., 2022). For examples, it has been conducted RNA-seq to study the mechanism of the interaction between gray leaf spot fungi and tomato (Zhang et al., 2022); it discovered the potential defense pathway of cucumber against downy mildew through RNA-seq (Gao et al., 2021). Previously, the transcriptome profiling analysis has been performed to study the interaction mechanism of *C. fulvum* and tomato carrying the resistance gene *Cf16* (Zhang et al., 2020). In our study, we used a universally susceptible cultivar moneymaker model plant which lacks any *Cf* resistance gene. We wondering the plant early defenses responses with fungal invasion in order to provide some theoretical basis for biocontrol of leaf mold disease at the early stage in future. Therefore, we analyze the transcriptome change during *C. fulvum* and tomato interaction at the time point of 24 hours which can be defined the early interaction period that a pathogen completes the invasion of a host plant (Shen et al., 2017). Gene ontology (GO) terms and Kyoto Encyclopedia of Genes and Genomes (KEGG) databases have been used to annotate functions of differentially expressed genes (DEGs) in tomato after *C. fulvum* inoculation. We first focused on analysis of DEGs involved in plant-pathogen interaction pathways. As we expected, a set of genes related to plant defense have been induced. Next, we sought to identify DEGs involved in plant hormone signal transduction pathways and DEGs related with secondary metabolism. Further, our results showed that SA and ABA contents increased while JA contents decreased after *C. fulvum* invasion, which are consistent results with the relative expression level of genes involved in SA, JA and ABA dependent signaling pathways. Together, our results will broaden our understanding for investigating the mechanism of *C. fulvum* and tomato interaction.

## Materials and methods

### Plant materials and *Cladosporium fulvum* infection

The tomato (*Solanum lycopersicum*) cultivar Moneymaker which is susceptible to *C. fulvum* was used in this work. All tomato plants were grown in pots containing nutrient soil in a plant growth incubator with photoperiod conditions of 16h/8h, control temperatures of 26°C/18°C, and humidity set at 75%. *Cladosporium fulvum* was kindly provided by the Institute of Plant Protection, Chinese Academy of Agricultural Sciences. Conidia of* C. fulvum* were harvested from one-week-old PDA plates with distilled water, and adjust the spore suspensions concentration to 1× 10^6^ conidia/ml (de Wit et al., 2012). Tomato plants grown in soil at 3 weeks old were sprayed with a conidial suspension (1× 10^6^ conidia/ml) on both adaxial and abaxial sides of the leaves (Van Esse et al., 2007). After 24 h of inoculation, the third leaves below the growing point were collected from treated and control plants separately, rapidly frozen in liquid nitrogen, and stored at -80°C (Yu et al., 2015). Remaining plants were monitored for disease progression for up to 20 days. Three biological replicates were performed.

### RNA extraction, library preparation, and sequencing

Leaf surface sprayed with *C. fulvum* (1× 10^6^ conidia/ml) or sterile water. At 24 h after inoculation (hai), leaf samples were harvested and total RNA was isolated using the Trizol method (Meng and Feldman, 2010; Gao et al., 2014a). The RNA concentration was quantified by a NanoDrop-2000 nucleic acid spectrophotometer (Thermo Fisher Scientific, Wilmington, DE). After detecting the RNA integrity in 1% agarose gel, mRNA was enriched from a pool of RNA by Oligo-dT magnetic beads. Based on the manufacturer’s instructions, RNA was sheared into small pieces by using RNA fragmentation kit (Illumina, San Diego, CA, USA). cDNA was synthesized by reverse transcription with N6 primers. The cDNA fragments were subjected to end repair and adapter ligation. Following the PCR amplification, the PCR products were used to generate cDNA libraries. Quality control and library construction were entrusted to Huada Gene Technology Company (Wuhan, Hubei, China). The cDNA sequencing was carried out with BGISEQ-500 platform for generating raw reads. The RNA-seq data for *C. fulvum* inoculated samples (accession no: SRR21437047、SRR21437048、SRR21437049) and control-inoculated samples (accession no: SRR21437044、SRR21437045、SRR21437046) are available at the NCBI gene expression omnibus server (https://www.ncbi.nlm.nih.gov/geo/).

### Processing of sequencing data

Raw data obtained from BGISEQ-500 platform are subsequently subjected to quality control (QC) to determine whether the sequencing data are suitable for further analysis. The filtering is performed by SOAPnuke software as follows: first, remove the reads containing junction (junction contamination); second, remove the reads with unknown base N content greater than 5%; finally, reads with over half of the component bases with a quality score below 15 were defined as low-quality reads and subsequently removed. After quality control, the filtered clean reads were mapped to the reference genome sequence (GCF_000188115.3_SL2.50, https://www.ncbi.nlm.nih.gov/assembly/GCF_000188115.3/) using HISAT (Hierarchical Indexing for Spliced Alignment of Transcripts). After the alignment, the comparison result is judged by the statistics of the mapping rate and the distribution of reads on the reference sequence to pass the second QC of alignment. The dataset analysis was performed after meeting the requirement.

### Functional annotation and enrichment pathway analyses of DEGs

DEGs with |Log_2_ Fold Change| ≥ 1 and Q-value ≤ 0.05 were functionally classified and enriched using the phyper function in R software to calculate *P*- value, False discovery rate (FDR) correction was then performed to obtain a normalized *P*-value, also known as Q-value. Gene Ontology (GO, http://geneontology.org/) annotation and Kyoto Encyclopedia of Genes and Genomes (KEGG, https://www.genome.jp/kegg/) pathway enrichment were used to analysis DEGs. KEGG pathways with an adjusted Q-value of ≤0.05 were regarded as significantly enriched (Gao et al., 2014b).

### Quantitative real-time PCR

A total of randomly selected 10 genes were used for the measurement of transcript abundance by quantitative real-time PCR to verify the reliability of the transcriptome data. Total RNA was extracted from frozen leaf tissue using Trizol reagent according to the manufacturer’s instructions. The integrity of RNA was analyzed by 1% (w/v) agarose gel electrophoresis (Gao et al., 2017). The reverse transcription was performed using the All-in-One First-Strand Synthesis MasterMix Kit (with dsDNase) (Lablead, Beijing,China), while qRT-PCR analysis was conducted using the TB GreenTM Premix Ex TaqTM kit (TaKaRa, Shanghai, China) with a RealTime PCR System (Bio-Rad, Hercules, CA, USA). The PCR running procedure was as follows: a pre-denaturation at 95°C for 30 seconds, 40 cycles with each cycle employing a denaturation temperature at 95°C for 5 seconds and an annealing/extension temperature at 60°C for 30 seconds, followed by melt curve analysis. For each biological sample, three technical replicates were used and there was a total of three biological replicates. Relative quantification of genes was carried out using the 2^-ΔΔCT^ method. The primer sequences for the ten genes are listed in **Table 1**. *SlACTIN* (LOC101260631) served as an internal control. The same method was used to detect the relative expression of six genes related to plant hormones signaling pathway with specific primers listed in **Table 2**. This experiment was done in three independent biological replicates.

**Table 1:** Primer sequences used for qRT-PCR validation in this study.

Gene/Gene number	Forward	Reverse	Product length
*SlACTIN*	GATGGTGGGTATGGGTCAAA	AGGGGCTTCAGTTAGGAGGA	199
LOC101249624	ATGCCGATGGATACCGAAACA	CGAGAACTGAACTCCGAAAGA	131
LOC109119038	GATGGCTTCACACTTCCCAAG	TACTCTCCATTTTGGTATCCC	141
LOC101245298	GTCAAGCCTTTTGGGTTATCG	AGGATCCACTTCGTTCATCAT	134
LOC101266084	GAAGCCTTTGAAGGACCATCT	AAGCTTTCCACACTGCTGGTA	112
LOC101246590	GGAATGGAATTAGGGTTTGGC	AAATTGAAGGACCTCTTGTGC	99
LOC101263535	AAATTGTGGAAGTAATCTCTG	GCATTGACATCATTAAAGTCC	103
LOC101265854	GCTCGTGGTCAAGTCGGGGTT	GACCAGAATGAATCAAGTTGC	112
LOC104648161	CAAATGCATGTCCCTTGTGTT	CAGATGAAAATCTACCTGACG	142
LOC101258353	GTTACTTTGTGCTTCAGCCAA	TCGGAGAGTGAGCTGGTGAGT	138
LOC101267111	AGCAGCTTCTTACATGTCAAC	TGGTTATGAGAACACGATCAA	116

**Table 2:** Primer sequences for verification of gene expression related to plant hormones signaling pathways used in this study.

Gene	Forward	Reverse	Product length
*SlACTIN*	GATGGTGGGTATGGGTCAAA	AGGGGCTTCAGTTAGGAGGA	199
*SlNPR1*	GGTCAGTGTGCTCGCCTAT	TGAAAGGTAAAGGATGCGT	150
*SlPR1*	CACCACTTGATCAAAAAAGTCTAG	TGAATGAATAAGTCTACAATCTTC	227
*SlSRK2C*	CGGATATTCGTAGCTGATCCA	TACTAAACTAGCTTCCTCTCC	120
*SlPYR1*	CGTTCATCAGGAAGCAGAAGA	CACTTGATTTGAGCTCATCGG	98
*SlLoxC*	AACACCGTTTACTCCGCCCTA	AGTCCTGAAAGATCGACACCC	131
*SlAOC*	TCGGAGATCTTGTCCCCTTTA	CGTGCTTGATCAGAATGCAGA	96

### Detection of reactive oxygen species burst

Leaves were taken from 3-week-old plant, and a hole punch was used to make a 6 mm diameter round sample from the same part of every leave. The round leave samples were placed in a container with distilled water for 9 h at room temperature to get rid of the mechanical damage for the leaves. Horseradish peroxidase (Shenggong, Shanghai, China) was dissolved in sterile water to make a working solution with a final concentration of 10 mg/ml. *C. fulvum* were maintained on potato dextrose agar (PDA) at 22°C prior to use, and diluted the spore suspensions in sterile water to 1×10^6^ conidia/ml (de Wit et al., 2012). Mixed solution with 1μl horseradish peroxidase working solution, 1μl conidial suspension (1×10^6^ conidia/ml) as treatment or 1μl sterile water as the control, and 98μl of ECL (enhanced chemiluminescence) solution (Shenggong, Shanghai, China) was as ROS determination solution. For ROS measurement, the cut leaves were removed from water container and put in ROS determination solution immediately. The machine used to measure ROS production is Glomax microplate luminometer (Promega, Wisconsin, USA). One value was detected every 10 seconds, for a total of 160 values per sample. The trend of the values indicates the degree of ROS accumulation (Robineau et al., 2020). The results are presented as means ± SE of three biological and three technical replicates.

### Determination of SA, ABA and JA contents

Spore suspensions with *C. fulvum* (1×10^6^ conidia/ml) were sprayed uniformly on the adaxial and abaxial surfaces of leaves, using sterile water as a control. At 24 h after inoculation, 3-4 leaf samples were taken from three plants for each treatment. The collected samples were immediately frozen in liquid nitrogen and stored at -80°C. The contents of SA, ABA and JA were determined simultaneously by ultra performance liquid chromatography (UPLC) LC-30A coupled with a triple quadrupole mass spectrometer LCMS-8040 (Waters, Massachusetts, USA). The leaf samples prepared in advance were placed on ice and transferred to the rapid nucleic acid extractor. Rapid nucleic acid extraction reagent (900 mg) and 1 ml of ethyl acetate extraction reagent were added and shaken for 45 seconds. The resulting solution was centrifuged at 4°C for 20 min at 16000 g and the supernatant was extracted and evaporated at 30°C under reduced pressure. The dried sample was dissolved in 0.5 ml of 70% methanol solution and centrifuged at 16000 g for 20 min. The supernatant obtained was filtered and put on the machine. The mobile phase consisted of binary gradients of acetonitrile with 0.01% (v/v) formic acid and 0.01% (v/v) aqueous formic acid, with a flowing speed at 0.5 ml/min. A 20 µL portion of each sample was injected into the UPLC-ESI-MS/MS system (Simura et al., 2018; Xin et al., 2020). The results are presented as means ± SE of three biological replicates and every biological repeat includes three technical replicates.

## Results

### Symptoms induced by *Cladosporium fulvum* inoculation in tomato plants

**Figure 1:**
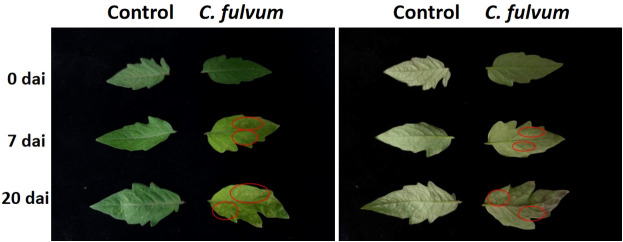
Symptoms after tomato leaves infected by *Cladosporium fulvum.* Tomato leaves were sprayed exogenously with a conidial suspension (1×10^6^ conidia/ml) of *C. fulvum* on the adaxial and abaxial sides of the leaves, water as a control treatment. Pictures were taken from 0 days, 7 days and 20 days after inoculation. The left half of the figure is the adaxial side of the leaves. The right half of the figure is the abaxial side of the leaves. The disease symptoms are more obvious in the places marked by red circles. dai: days after inoculation.

In order to observe the symptoms induced by *C. fulvum*, a suspension of spores containing the leaf mold pathogen (1× 10^6^ conidia/ml) was sprayed on tomato leaves. Compared to the control group, pathogen-inoculated leaves with yellow spots at 7 days after inoculation. At 20 days after inoculation, yellow spotted area expanded; mold layer can be observed in the abaxial side of leaves and when severe leaves were obviously curled (**Figure 1**). These symptoms indicated successful infection of *C. fulvum* and the plant materials we taken at the time point of 24 hours were reliable for experiments.

### ROS production triggered by *Cladosporium fulvum* treatment

**Figure 2:**
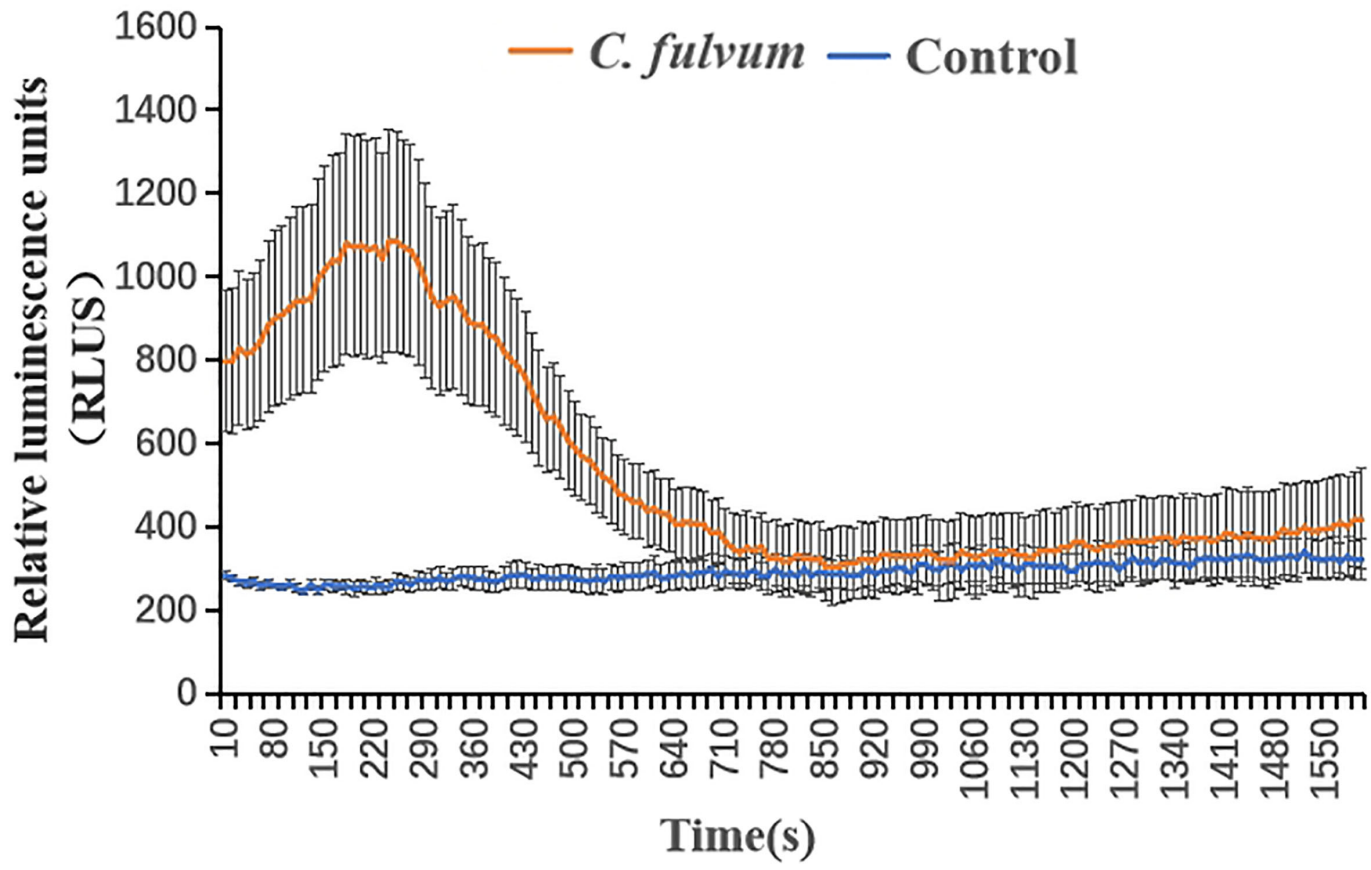
Production of reactive oxygen species (ROS) in tomato leaf disks after treatment with *Cladosporium fulvum*. Tomato leaf disks were treated with *C. fulvum* at the concentration of 1×10^6^ conidia/ml and water as control. ROS production was measured using the chemiluminescence of luminol and photon counts were expressed as relative luminescence units (RLUs). The *X*-axis indicates time, and the instrument measures one value every 10 s, for a total of 160 values, *Y*-axis indicates the level of ROS accumulation by relative luminescence units. The results are presented as means ± SE of three biological and three technical replicates.

Detection of high levels of ROS, a signal substance, can reflect the ability of the plant to resist pathogen at some extent (Hernandez et al., 2016). The leave samples were firstly put in distilled water up to 9 hours to avoid impact caused by mechanical damage, then we measured the ROS level of tomato leaves treated with *C. fulvum.* Results showed that the timing and accumulation of ROS production in tomato leaves in response to *C. fulvum* were different compared with control treatment. Tomato leaves treated with *C. fulvum* produced a peak of ROS accumulation and the overall value was higher than the control (**Figure 2**). This indicates that *C. fulvum* infection activates an early ROS burst in leaves.

### Overview of the *Solanum lycopersicum* transcriptome

Tomato infected by *C. fulvum* was used as the treatment group and sterile water treatment was used as the control group for transcriptome sequencing. Three biological repeats were performed for each group. A total of six samples were sequenced using the DNBSEQ platform, and each sample yielded an average of 1.19G of data. We obtained an average of 23.81 M high-quality reads per sample, accounting for > 99% of the raw reads for each sample. The reads with a quality value of 30 accounted for > 95% of the total reads and a quality value of 20 account for > 98% of the total reads, indicating that the sequencing reads are of high quality (**Table 3**). Hierarchical Indexing for Spliced Alignment of Transcripts (HISAT) was used to align the clean reads to the reference genome sequence (http://daehwankimlab.github.io/hisat2/) after obtaining the clean reads. The average alignment rate to the reference genome of *Solanum lycopersicum* was 97.54%, indicating the high quality of the data, guaranteeing the reliability of subsequent differentially expressed genes (DEGs) analysis.

**Table 3:** Quantitative analysis and comparison ratio of raw RNA-seq data.

Sample	Total Raw Reads (M)	Total Clean Reads (M)	Clean Reads Q20 (%)	Clean Reads Q30 (%)	Clean Reads Ratio (%)	Genome Total Mapping (%)
Control_1	23.92	23.79	98.43	95.28	99.44	97.71
Control_2	23.92	23.71	98.43	95.23	99.12	97.02
Control_3	23.92	23.86	98.5	95.41	99.75	97.7
*C. fulvum*_1	23.92	23.76	98.42	95.25	99.31	97.53
*C. fulvum*_2	23.92	23.85	98.54	95.54	99.69	97.64
*C. fulvum*_3	23.92	23.87	98.49	95.43	99.76	97.66

**Figure 3:**
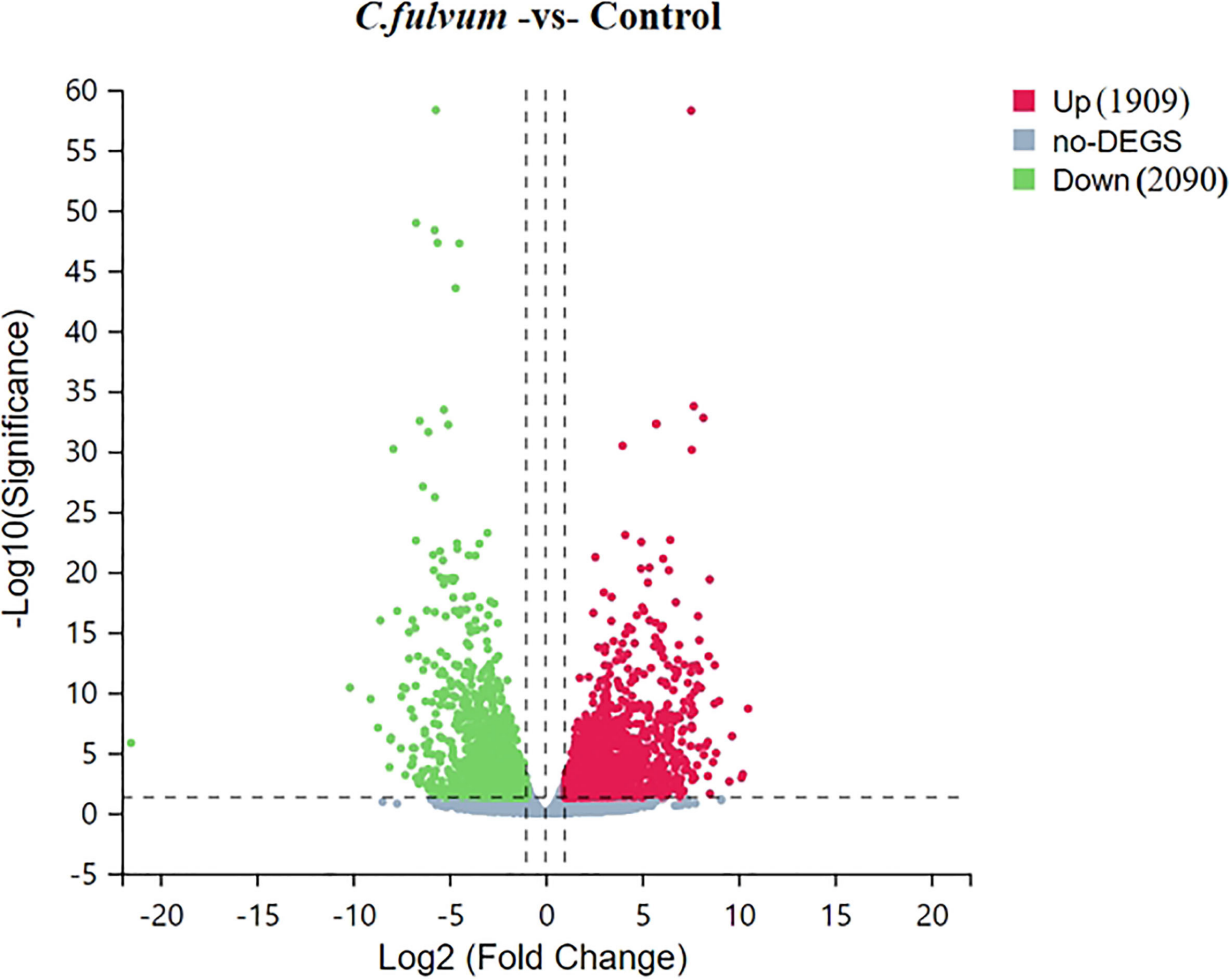
Volcano plot of upregulated and downregulated differentially expressed genes (DEGs) after *Cladosporium fulvum* inoculation. *X*-axis represents log_2_ transformed difference multiplier values and *Y*-axis represents -log_10_ transformed significance values. Red dots represent upregulated DEGs, green dots represent downregulated DEGs, and gray dots represent that were not significantly different between *C.fulvum* and control treatment.

Clean reads were compared to reference gene sequences by Bowtie2 software, and then gene expression levels of individual samples were calculated using RNA-Seq by Expectation-Maximization (RSEM) (deweylab.github.io), followed by DEGs screening according to the method described by Michael (Love et al., 2014). The significant DEGs were determined based on the criteria of |Log_2_ Fold Change| ≥ 1 and Q-value ≤0.05, the distribution of DEGs based on degree of difference and the significance of the difference were visualized with a volcanic plot (**Figure 3**). Compared to the control, *C. fulvum* inoculation plants induced 1909 upregulated DEGs and 2090 downregulated DEGs (**Tables S1**, **S2**).

### Gene ontology enrichment analysis of differentially expressed genes

**Figure 4:**
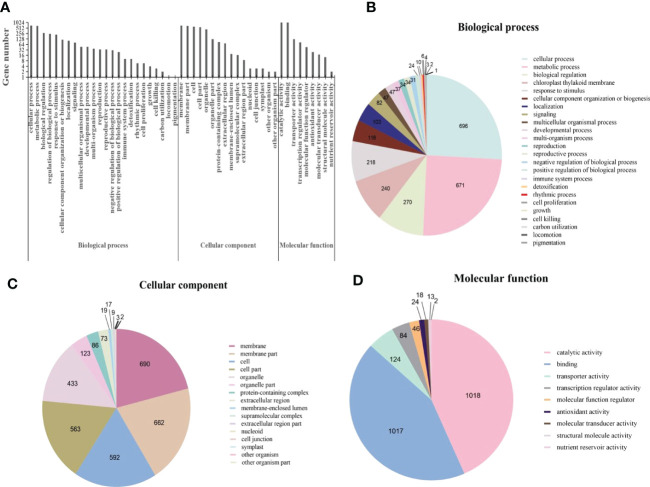
Gene ontology (GO) annotations for differentially expressed genes (DEGs) in *Cladosporium fulvum* inoculated tomato leaves *vs* control treatment. **(A)** GO classifications of differential genes in *C fulvum vs* control treatment. Pie charts showing the breakdown and prevalence of enriched GO terms in the following categories: biological process **(B)**, cellular component **(C)** and molecular function **(D)**.

Gene Ontology (GO) is an international standardized gene function classification system. Based on GO terms, DEGs were classified into three major categories: biological process, cellular component, and molecular function. Within the broad GO category of biological process, a total of 2693 DEGs were involved in 24 GO terms (**Figure 4**). According to previous reports, terms with “cellular process”, “metabolic process”, “biological regulation”, “regulation of biological process”, “response to stimulus” were associated with plant disease resistance (Zhang et al., 2020). In the classification of cellular component, there were 3280 DEGs involving 16 GO terms (**Figure 4**), DEGs mainly concentrated in the integral component of membrane. In the molecular function category, more than 85% of the 2346 DEGs were mainly enriched in the “catalytic activity” and “binding” (**Figure 4**). The enrichment of DEGs in these two terms has been found to associate with the induction of many genes involved in plant hormone signal transduction pathway (Zhang et al., 2020; Singh et al., 2021).

### Kyoto Encyclopedia of Genes and Genomes enrichment analysis of differentially expressed genes

The KEGG (Kyoto Encyclopedia of Genes and Genomes) database is a database that systematically analyzes gene functions, links genomic information and functional information. Through KEGG enrichment analysis, *C. fulvum* infection induced 830 DEGs involved in 125 KEGG pathways. Fifteen pathways with *P-* values ≤0.05 were selected based on the number of involved DEGs of corresponding pathways arranged in a descending order (**Table 4**). The KEGG pathway with the highest number of enriched DEGs was Plant hormone signal transduction pathway (ko04075) involving in 61 DEGs, which includes 40 upregulated DEGs and 21 downregulated DEGs (**Table S3**). The results above suggest that plant hormone pathways were significantly affected by *C. fulvum* infection. More importantly, many pathways associated with plant disease resistance were activated, such as Plant-pathogen interaction (ko04626), MAPK signaling pathway – plant (ko04016), and Peroxisome (ko04146) (Nyathi and Baker, 2006; Jiang et al., 2022a). In addition, many metabolic pathways such as Starch and sucrose metabolism (ko00500) and Circadian rhythm – plant (ko04712) were also altered, indicating that pathogenic fungal infection can also affect normal plant growth and development.

**Table 4:** The Kyoto Encyclopedia of Genes and Genomes (KEGG) pathways (top 15) of differentially expressed genes (DEGs) enrichment by *C. fulvum* infection.

PathwayID	KEGG Pathway	Gene Num	upregulatedDEGs	downregulatedDEGs	*P*- value
04075	Plant hormone signal transduction	61	40	21	0.02
00940	Phenylpropanoid biosynthesis	47	26	21	0.03
04626	Plant-pathogen interaction	45	33	12	0.05
04016	MAPK signaling pathway - plant	45	34	11	2.56E-3
00500	Starch and sucrose metabolism	42	12	30	8.12E-5
00480	Glutathione metabolism	31	19	12	2.39E-3
00230	Purine metabolism	25	9	16	9.00E-3
04146	Peroxisome	23	13	10	0.02
00561	Glycerolipid metabolism	22	17	5	0.01
00562	Inositol phosphate metabolism	20	10	10	0.02
01212	Fatty acid metabolism	20	10	10	0.03
00941	Flavonoid biosynthesis	19	9	10	2.09E-3
04712	Circadian rhythm - plant	17	8	9	4.04E-4
00908	Zeatin biosynthesis	17	9	8	0.04
00592	Alpha-Linolenic acid metabolism	14	7	7	0.03

### A set of core genes involved in plant defense were upregulated after *Cladosporium fulvum* inoculation

In our study, a total of 45 DEGs related with the plant defense responses were identified after *C. fulvum* inoculation, in which 33 genes were upregulated and 12 genes were downregulated (**Table S4**). These genes can be considered indicators of plant defense in response to *C. fulvum* invasion. A total of six serine/threonine-protein kinase receptors or receptor like kinases were identified (**Table 5**), all of which were upregulated after *C. fulvum* inoculation. In particular, two genes encoding of LRR receptor-like serine/threonine-protein kinase FLS2 (flagellin sensing 2) and FLS3 (flagellin sensing 3) that binds to flg22 and flgII-28 respectively (Hind et al., 2016; Chi et al., 2021) were significantly induced to 6-fold and 27-fold compared to the control treatment. And gene encoding SERK3B (somatic embryogenesis receptor kinase 3B), which interacts with FLS2 for activating downstream signaling (Ma et al., 2022), was also slightly upregulated. This result indicated that tomato activated the intracellular immune signaling responses after *C. fulvum* invasion.

**Table 5:** Differentially expressed genes related with the plant defense responses after *Cladosporium fulvum* treatment.

Gene ID	Gene Symbol	Seq Description	Log_2_ (*C.fulvum*/Control)	*P*-value
101246110	LOC101246110	LRR receptor-like serine/threonine-protein kinase EFR	2.20	1.97E-04
101248095	*FLS3*	FLAGELLIN-SENSING 3 protein	4.76	1.80E-08
101256183	LOC101256183	Serine/threonine-protein kinase PBS1	0.81	0.003420758
101257866	LOC101257866	Probable serine/threonine-protein kinase PBL7	4.75	1.48E-08
101260980	LOC101260980	Probable LRR receptor-like serine/threonine-protein kinase At3g47570	3.41	0.003189953
101263667	*FLS2*	LRR receptor-like serine/threonine-protein kinase FLS2	2.62	1.82E-04
100736531	*SERK3B*	Somatic embryogenesis receptor kinase 3B	0.87	0.002665399
101055527	LOC101055527	Hop-interacting protein THI080	1.45	9.70E-04
101260391	LOC101260391	Calcium-dependent protein kinase 18-like	2.10	0.008481838
101244290	LOC101244290	Calmodulin-like protein 3	3.15	1.68E-09
101245298	LOC101245298	Calmodulin	4.78	4.67E-06
101245539	LOC101245539	Probable calcium-binding protein CML44	1.24	0.00819727
101257476	LOC101257476	Calmodulin-like protein 8	4.16	2.38E-04
101264550	LOC101264550	Calcium-binding protein CP1	5.09	2.52E-09
101265816	LOC101265816	Caltractin	1.06	0.007541617
112940015	LOC112940015	Calmodulin-like protein 1	1.81	4.66E-04
101245220	*CER6*	3-ketoacyl-CoA synthase 6	0.74	0.007012538
101262858	LOC101262858	3-ketoacyl-CoA synthase 20-like	1.67	1.40E-05
101268230	LOC101268230	3-ketoacyl-CoA synthase 1	2.67	1.06E-09
101268257	LOC101268257	3-ketoacyl-CoA synthase 11-like	4.88	0.004680944
101252097	LOC101252097	WRKY transcription factor 1	2.12	5.29E-05
101260537	LOC101260537	Probable WRKY transcription factor 26	3.09	3.29E-10
100191111	LOC100191111	PR1 protein	6.51	2.22E-09
544123	*PR1b1*	Pathogenesis-related leaf protein 6	7.54	1.38E-14
544185	*P4*	Pathogenesis-related protein P4	8.69	2.47E-06
101246133	LOC101246133	Calcium-dependent protein kinase 29	-2.37	1.23E-08
101250418	LOC101250418	Calcium-dependent protein kinase 24	-1.81	0.006829089
101255379	LOC101255379	Calcium-dependent protein kinase 17-like	-3.60	4.70E-08
101256200	LOC101256200	Calcium-dependent protein kinase 1	-2.30	2.84E-12
101244728	LOC101244728	Caltractin	-3.98	4.44E-06
543984	*CaM6*	Calmodulin 6	-1.13	0.005832385

In response to various stimuli in plants, Ca^2+^ acts as a second messenger to modulate different target protein activities through CAM/CML receptors, thereby regulating a variety of cellular functions (Zhang et al., 2014). A total of 15 DEGs related with Ca^2+^ signaling were identified upon *C. fulvum* infection with 9 upregulated and 6 downregulated genes (**Table 5**). Genes encoding a few of calmodulin (CML) or calmodulin like proteins such as calmodulin (LOC101245298), *CML44* (LOC101245539), calcium-binding protein (LOC101264550, *CP1*), and one potential CDPK protein (LOC101260391, calcium-dependent protein kinase 18-like) were upregulated. Four genes encoding CDPK were downregulated after *C. fulvum* infection, including *CDPK1* (calcium-dependent protein kinase 1), *CDPK17*-like, *CDPK24* and *CDPK29*, which is consistent with previous report that *CDPK1* expression was decreased after 1.5 h treatment with *C. fulvum* (Chico et al., 2002). This result indicated that differently expression of CDPK genes are involved in the complex signaling network of tomato in response to *C. fulvum* invasion.

In addition, four genes encoding 3-Ketoacyl-CoA synthase (KCS), which catalyzes a condensation reaction to form 3-ketoacyl-CoA during very long chain fatty acid synthesis, including *KCS1*, *KCS6*, 3-ketoacyl-CoA synthase 20-like (LOC101262858) and 3-ketoacyl-CoA synthase 11-like (LOC101268257) were all upregulated in our study (**Table 5**). These gene expression might contribute to plant cuticular wax and suberin biosynthesis after pathogen invasion (Edqvist et al., 2018). Moreover, two transcriptional factors WRKY26 and WRKY1, which have been reported as key components of resistance in tomato against *Alternaria solani* (Shinde et al., 2018), were induced in our study. Further, several defense responses related genes such as those encoding pathogenesis-related (*PR*)1 protein (LOC100191111) (Lavrova et al., 2017), *PR1b* (Hoegen et al., 2002), and *P4* (Pieterse and van Loon, 1999) were significantly induced. Together, our data analysis suggested that plant defense pathways are activated after *C. fulvum* inoculation in tomato plant.

### Expression of genes related to multiple hormones affected by *Cladosporium fulvum* inoculation

Next, we chose to analyze DEGs related to plant hormone signal transduction pathway which play key roles in regulating plant defense response against pathogen (**Table S3**). In the SA signaling pathway, a total of 9 DEGs were identified; 6 genes were upregulated and 3 genes were downregulated. The downregulated genes include encoding the transcription factor TGA1 (TGACG MOTIF-BINDING FACTOR 1), pathogenesis-related leaf protein 4 (LOC101265854) and BOP2 (BLADE-ON-PETIOLE protein), which is associated with leaf and flower development in *Arabidopsis* (Zhang et al., 2017). The upregulated genes include a SA receptor *NPR1* (Nonexpressor of PR1, also known as *NIM1*) (Li et al., 2019b), *NML2* (NPR1/NIM1-like protein), *TGA2.2* (Hou et al., 2019) and PR genes (*PR-1b1* and *P4*) (**Table 6**). This result suggested that SA signaling pathway are activated after *C. fulvum* invasion given that PR1 is an indicator for activation of the SA signaling pathway in plants.

**Table 6:** Differentially expressed genes related with plant hormones signaling pathway affected by *Cladosporium fulvum* treatment.

Pathway	Gene ID	Gene Symbol	Seq Description	Log_2_ (*C.fulvum*/Control)	*P*-value
SA	543939	*NPR1*	Regulatory protein NPR1	1.39	2.07E-04
	544270	*NML2*	NIM1-like protein 2	1.94	1.83E-07
	543600	*TGA2.2*	Transcription factor TGA2.2	1.15	0.001692675
	544123	*PR1b1*	Pathogenesis-related leaf protein 6	7.54	1.38E-14
	544185	*P4*	Pathogenesis-related protein P4	8.69	2.47E-06
	100191111	LOC100191111	PR1 protein	6.51	2.22E-09
	101246001	*BOP2*	BLADE-ON-PETIOLE protein BOP2	-3.21	0.006220984
	101265431	LOC101265431	Transcription factor TGA1	-2.12	5.31E-04
	101265854	LOC101265854	Pathogenesis-related leaf protein 4	-2.38	1.94E-06
ABA	101246807	LOC101246807	Abscisic acid receptor PYL9	1.06	0.008230946
	101258886	LOC101258886	Abscisic acid receptor PYL3	1.07	0.003742465
	101267127	LOC101267127	Abscisic acid receptor PYR1	0.97	0.005559441
	101265524	LOC101265524	Protein phosphatase 2C 53	2.68	1.60E-09
	100037510	*SRK2C*	SNF1-related kinase	1.21	0.003101157
	101249794	LOC101249794	Protein phosphatase 2C 51-like	-2.19	0.004197722
	101251432	LOC101251432	Serine/threonine-protein kinase SRK2I	-2.84	3.23E-12
	100820704	*ABF4*	ABA responsive transcription factor	-1.10	0.006243886
JA	101266902	*AOS2*	Allene oxide synthase 2	-2.05	2.30E-3
	544008	*LOXC*	Lipoxygenase	-3.31	3E-8
	544306	*AOC*	Allene oxide cyclase	-1.40	8.09E-3
ET	101249950	LOC101249950	ETHYLENE INSENSITIVE 3-like 3 protein	2.11	0.002604271
	101246590	LOC101246590	Ethylene-responsive transcription factor 1B	4.73	1.18E-19
	543712	*EREB*	Ethylene responsive element binding protein	5.26	5.03E-05
	606712	LOC606712	Ethylene-responsive transcription factor 1	3.19	3.68E-05

We further analyzed genes involved in the ABA signaling pathway affected by *C. fulvum* invasion. The complex consisting of the ABA receptor PYR/PYL/RCAR (Pyrabactin resistance/PYR-like/regulatory components of ABA receptor), PP2C (type 2C protein phosphatase), and SnRK2s (Sucrose Non-fermentation Kinase Subfamily 2) has a key role in ABA signaling (Guo et al., 2011). In our study, genes encoding abscisic acid receptor PYL9 (LOC101246807), PYL3 (LOC101258886), PYR1(LOC101267127), PP2C (protein phosphatase 2C 53)and SRK2C (SNF1-related kinase), analogous to subclass III SnRK2s (Mizoguchi et al., 2010), were all upregulated after *C. fulvum* invasion. In addition, LOC101249794 encoding protein phosphatase 2C 51-like, SRK21 (LOC101251432) encoding serine/threonine-protein kinase SnRK21 and ABF4 (ABA responsive transcription factor) (Orellana et al., 2010) were downregulated (**Table 6**). Previous study suggested that once PYR/PYLs binds to ABA, it represses PP2C activity and releases SnRK2 suppression, triggering downstream ABA responses (Yang et al., 2017). Our data analysis indicated that ABA signaling might be activated after *C. fulvum* invasion.

In the JA synthesis pathway, the genes *LoxC* (lipoxygenase), *AOS2* (allene oxide synthase 2), and *AOC* (allene oxide cyclase) related to JA synthesis (Liu et al., 2012) were downregulated 2-8 fold (**Table 6**). And four genes involved in the ethylene signaling pathway were upregulated, including *ERF1* (ethylene response transcription factor 1), LOC101246590 (ethylene-responsive transcription factor 1B), *EREB* (ethylene responsive element binding protein) (Mingchun et al., 2016) and LOC101249950(ETHYLENE INSENSITIVE 3-like 3 protein) (**Table 6**). These results suggested that *C. fulvum* inoculation impacted the JA and ET signaling pathway differently in plants.

A total of 24 DEGs related to auxin signaling pathway were identified after *C. fulvum* invasion, of which 13 were upregulated and 11 were downregulated (**Table 7**). The *SAUR* (small auxin-up RNA) genes can respond not only to auxin but also to internal and environmental stress with mounting dynamic spatial-temporal responses (Wang et al., 2020). In particular, 11 *SAUR* genes were identified to be affected after *C. fulvum* treatment. Among these 11 DEGs, genes such as *SAUR32*, *SAUR58*, an *SAUR71* were upregulated and those *SAUR71-like* (LOC101248065, LOC101257321) genes were downregulated. The other class of DEGs affected by *C. fulvum* inoculation was IAAs; upregulated genes include *IAA4*, *IAA14*, *IAA35*, *IAA7*, and downregulated genes include *IAA15*, *IAA17*, and *IAA19* (Audran-Delalande et al., 2012). One gene *ARF5* (auxin response factor 5) was inhibited while the gene *ARF1* (auxin response factor 1) was slightly induced (Zouine et al., 2014). Two genes encoding Auxin transporter-like protein LAX2 and LAX5 were downregulated (Pattison and Catala, 2012). The above results indicate that *C. fulvum* infection does affect the auxin-related pathways in tomato plants in a complicated manner.

**Table 7:** Differentially expressed genes related with auxin signaling pathway affected by *Cladosporium fulvum* treatment.

Gene ID	Gene Symbol	Seq Description	Log_2_ (*C.fulvum*/Control)	*P*-value
101246226	LOC101246226	Auxin-responsive protein SAUR32	2.02	2.33E-04
101055583	LOC101055583	Small auxin-up protein 58	3.18	2.27E-12
101253234	LOC101253234	Auxin-responsive protein SAUR50-like	2.23	0.00387351
101250847	LOC101250847	Auxin-responsive protein SAUR50-like	6.60	2.18E-07
101265243	LOC101265243	Auxin-responsive protein SAUR71	3.24	8.21E-04
104645436	LOC104645436	Auxin-responsive protein SAUR21-like	1.81	0.001693518
101255303	*IAA4*	Auxin-responsive protein IAA4	3.22	1.28E-10
543542	*IAA7*	IAA7 protein	1.88	3.94E-05
101055547	LOC101055547	IAA14	0.84	0.003315808
101055555	LOC101055555	IAA35	1.80	0.009160947
104645435	LOC104645435	Auxin-induced protein 15A-like	1.98	5.94E-04
109118704	LOC109118704	Auxin-induced protein 15A-like	2.31	4.33E-04
100736509	*ARF1*	Auxin response factor 1	1.17	0.00645183
101246270	LOC101246270	Auxin-responsive protein SAUR36-like	-2.09	6.36E-04
101248065	LOC101248065	Auxin-responsive protein SAUR71-like	-5.45	0.002862421
101257321	LOC101257321	Auxin-responsive protein SAUR71-like	-3.85	4.31E-05
101248844	LOC101248844	Auxin-responsive protein SAUR50	-5.53	8.48E-04
101255313	LOC101255313	Auxin-responsive protein SAUR71	-1.74	0.006481308
101055548	*IAA15*	Auxin-regulated IAA15	-1.83	0.002256359
543544	*IAA17*	Auxin-responsive protein IAA17	-3.02	6.02E-05
101055549	*IAA19*	Auxin-responsive protein IAA19	-1.92	0.004643909
100736448	*ARF5*	Auxin response factor 5	-3.50	3.37E-11
100736477	*LAX2*	Auxin transporter-like protein 2	-3.38	4.80E-14
100736541	*LAX5*	Auxin transporter-like protein 5	-4.55	6.05E-04

### Genes involved in the plant phenylpropanoid pathway are affected by *Cladosporium fulvum* inoculation

Plant phenylpropanoid pathway is an important pathway for the synthesis of plant secondary metabolites, some of which have been proposed as important components of plant defense responses (Naoumkina et al., 2010; Piasecka et al., 2015). Its downstream metabolites mainly include coumarins, flavonoids, terpenoids, anthocyanins, lignin and other phenylpropanoids (Deng and Lu, 2017; Pratyusha and Sarada, 2022). In this study, many tomato DEGs were related with plant phenylpropanoid pathway after *C. fulvum* invasion. A total of 47 genes was enriched in the phenylpropanoid biosynthesis pathway, with 26 DEGs upregulated and 21 DEGs downregulated (**Table S5**). Phenylalanine ammonia lyase (PAL) is the first rate-limiting enzyme in the phenylpropane metabolic pathway (Ruili et al., 2016). *PAL2* (LOC101249824) was found to be slightly induced after invasion. The other two genes (LOC101244496, LOC101248210) encoding trans-cinnamate 4-monooxygenase and 4-coumarate-CoA ligase, two major players in this pathway, were also upregulated (**Table 8**). The gene encoding cinnamoyl-CoA reductase (CCR, LOC778359), a key enzyme in the formation of lignin monomers (Fan et al., 2015), was upregulated. Peroxidases (PRXs) are often found in lignifying tissues and they have the capability to oxidize a wide variety of small phenolic compounds, including monolignols (Marjamaa et al., 2009). In our study, there are 9 related genes upregulated such as *TAP2* (tomato anionic peroxidase 2, LOC101245316) (Melillo et al., 2014) and *CEVI-1* (citrus exocortis viroid) (Vera et al., 1993) encoding an anionic peroxidase in tomato, which could be induced by compatible viral infection (Mayda et al., 2000), while peroxidase 12, peroxidase 18, peroxidase 3, peroxidase 44 like and 45 like genes were downregulated (**Table 8**). Four genes related with flavonoid biosynthesis, *CHS1* (chalcone synthase 1), *CHS2* (Heredia et al., 2015), *CHI1* (chalcone-flavonoid isomerase 1) (Kang et al., 2014) and *DFR* (dihydroflavonol 4-reductase) (Bongue-Bartelsman et al., 1994) were significantly downregulated (**Table 8**). Both *ANS* (anthocyanidin synthase) and *F3H* (flavanone 3-dioxygenase) (Li et al., 2019c) involved in anthocyanin synthesis were also downregulated (**Table 8**). In addition, a few genes encoding beta-glucosidase, catalyzing the hydrolysis of cellobiose and cello-oligosaccharides containing (1 → 4)-beta-glycosidic bonds to glucose, which is crucial in cellulosic ethanol production were downregulated, such as LOC101254239, LOC101263519, LOC101256554, whereas only 1 beta-glucosidase 18-like (LOC101248595) was upregulated (**Table 8**), suggesting cellulosic ethanol production pathway was inhibited after *C. fulvum* invasion. Our results indicated that a variety of secondary metabolites related to plant disease resistance was affected by fungal infection.

**Table 8:** Differentially expressed genes induced by *C. fulvum* infection are involved in the production of secondary metabolites.

Pathway	Gene ID	Gene symbol	Seq Description	Log_2_(*C.fulvum*/Control)	*P*-value
Phenylpropanoid	101249824	*PAL2*	Phenylalanine ammonia-lyase 2	0.96	0.17
	101244496	LOC101244496	Trans-cinnamate 4-monooxygenase	1.38	1.32E-3
	101248210	LOC101248210	4-coumarate–CoA ligase	2.29	1.37E-6
Lignin	778359	*CCR2*	Cinnamoyl-CoA reductase	2.03	2.74E-4
	101267754	LOC101267754	Peroxidase 51	6.23	1.46E-4
	101265511	LOC101265511	Suberization-associated anionic peroxidase 2-like	6.22	5.3E-10
	101251503	LOC101251503	Peroxidase 21	5.82	1.94E-6
	101258529	LOC101258529	Peroxidase P7-like	5.28	4E-9
	101264425	LOC101264425	Peroxidase P7	5.09	9E-9
	101243845	LOC101243845	Peroxidase 72	4.83	2.86E-3
	101245316	*TAP2*	Suberization-associated anionic peroxidase 2	3.70	5.34E-4
	101261825	LOC101261825	Peroxidase P7	2.95	1.39E-3
	544084	*CEVI-1*	Peroxidase	2.58	3E-8
	101253377	LOC101253377	Peroxidase 12	-1.45	8.97E-4
	101253684	LOC101253684	Peroxidase 12	-1.77	2.55E-3
	101247048	LOC101247048	Peroxidase 45-like	-3.28	4.83E-4
	101254073	LOC101254073	Peroxidase 18	-3.57	7.70E-3
	101250356	LOC101250356	Peroxidase 44-like	-5.44	3.25E-3
	101250523	LOC101250523	Peroxidase 3	-6.39	1.84E-5
Flavonoid	778294	*CHS1*	Chalcone synthase	-5.13	1.4E-12
	778295	*CHS2*	Chalcone synthase	-4.11	9.1E-13
	101249265	*CHI1*	Chalcone–flavonone isomerase 1	-3.06	2.63E-5
	544150	*DFR*	Dihydroflavonol 4-reductase	-5.69	7.5E-11
Anthocyanin	101251607	*ANS*	Anthocyanidin synthase	-3.11	1E-7
	100736482	*F3H*	Flavanone 3-dioxygenase	-2.40	5.11E-6
Cellulosic ethanol	101248595	LOC101248595	Beta-glucosidase 18-like	2.08	1.01E-08
	101254239	LOC101254239	Beta-glucosidase BoGH3B	-5.13	5.09E-16
	101263519	LOC101263519	Beta-glucosidase BoGH3B	-2.17	0.005989607
	101256554	LOC101256554	Beta-glucosidase BoGH3B	-1.67	7.03E-05

### Quantitative real-time PCR validation of transcriptome results

**Figure 5:**
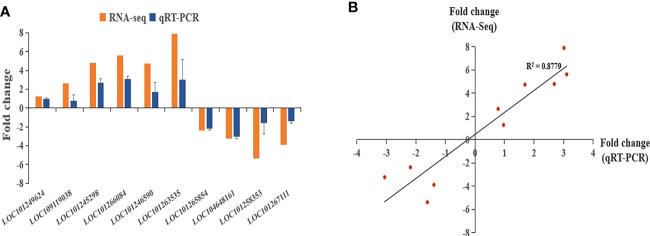
qRT-PCR validation of RNA-seq sequencing results. **(A)** qRT-PCR and RNA-seq comparison between ten randomly selected differentially expressed genes. The qRT-PCR values represent mean ± SE from three biological replicates. **(B)** Regression analysis comparing gene expression ratios by qRT-PCR and RNA-seq analysis.

To validate the reliability of transcriptome data, we randomly picked some upregulated and downregulated genes with one gene related to plant resistance. A total of 10 genes was selected including LOC101249624, LOC109119038, LOC101245298, LOC101266084, LOC101246590, LOC101263535, LOC101265854, LOC104648161, LOC101258353, LOC101267111 (**Figure 5A**). The expression data obtained from qRT-PCR verification had consistent trend with RNA-Seq, with a correlation coefficient of 0.8779 (**Figure 5B**), indicating the RNA-seq data were reliable.

### SA, ABA and JA contents in plants are affected by *Cladosporium fulvum* inoculation

**Figure 6:**
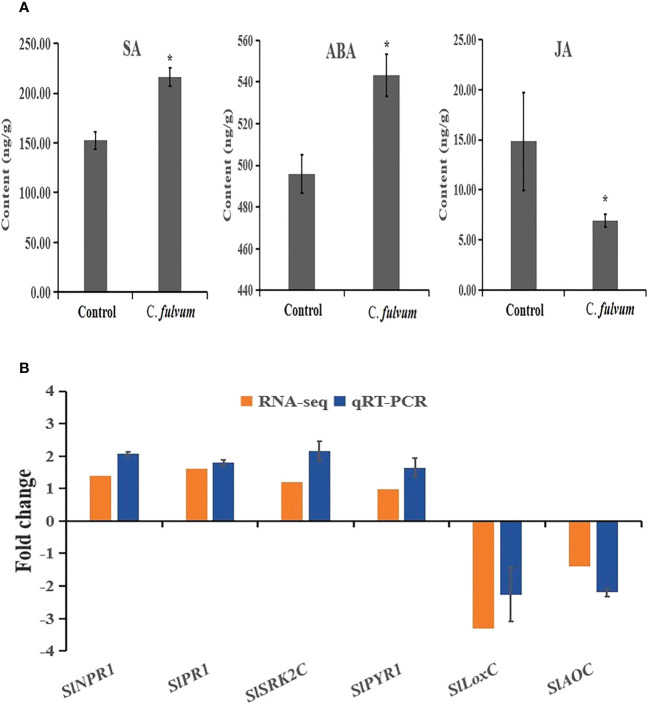
Hormone contents and relative gene expression levels impacted by *Cladosporium fulvum* treatment at the time points of 24 hours. **(A)** The contents of SA, ABA and JA in tomato leaves were detected 24 hours after sprayed exogenously with a conidial suspension (1X10^6^ conidia/ml) of *C fulvum*, water treatment as a control. Shown are the mean and SE of three biological replicates. Statistical differences were determined Student’s t-test (p<0.05). **(B)** Six genes related to plant hormone signal transduction pathway were tested expression level by *C fulvum* inoculation after 24 hours. *SlNPR1* and *SlPR1* are SA-related pathway genes, *SlSRK2C* and *SlPYR1* are ABA-related pathway genes, *SlLoxC* and *SlAOC* are JA-related pathway genes. The qRT-PCR values represent mean ± SE from three biological replicates. * indicate signifificant differences between control and C.fulvum with p < 0.05, as determined by t-test.

According to our RNA-seq data analysis, some key genes involved in SA and ABA signaling were upregulated after *C. fulvum* invasion. Thus, we decided to measure the content of SA and ABA. Our results showed that both SA and ABA contents increased after fungal invasion, indicating SA and ABA singling pathways were activated after *C. fulvum* treatment. We further tested the JA content and showed that JA level decreased after fungal invasion in comparison to the control treatment (**Figure 6A**). This is in consistence with the RNA-seq data in which a few genes related with JA synthesis was inhibited after C*. fulvum* inoculation.

### The related expression of genes involved in SA, ABA and JA signaling pathways after *Cladosporium fulvum* inoculation

To further confirm the impact to SA, ABA and JA hormone pathways after C*. fulvum* invasion, two genes related to each plant hormone signal transduction pathway were selected for qRT-PCR gene expression analysis. Results showed that the relative expression of genes with RNA-seq and qRT-PCR are the similar trend (**Figure 6B**). The qRT-PCR results showed that expression of ABA-related *SlSRK2C* and *SlPYR1*) and SA-related genes (*SlNPR1* and *SlPR1*) were upregulated 2-4 folds. In contrast, expression of synthetic genes of JA (*SlLoxC* and *SlAOC*) were significantly downregulated by more than 3-fold. Our results further confirmed that these three hormone pathways are affected after *C. fulvum* invasion.

## Discussion

Tomato leaf mold caused by *C. fulvum* results in seriously yield losses in tomato cultivation worldwide (Jia, 2010). Considering that the ability of resistance of some tomato cultivar has decreased because of many physiological races of *C. fulvum* rapidly mutated, we focused on investigating the transcriptome profiles during early stage of interaction with *C. fulvum* in tomato leaves by using susceptible tomato plants (Jiang et al., 2022b). As we expected, a variety of defense related genes are identified after *C. fulvum* inoculation, such as *PR1*, *HSP90*, and *WRKY1*, which is consistence with the previous findings (Zhao et al., 2019; Wang et al., 2022). Based on previous reported that flg22 (flagellin 22) binding induces FLS2-BAK1 (BRI1-associated kinase1) heteromerization to activate immune responses in *Arabidopsis thaliana* (Sun et al., 2013). In our study, we found that genes *FLS2*, *FLS3* and *EFR* were all upregulated after *C. fulvum* inoculation, indicating that tomato plants might share the common PAMP pathways in response to biotroph fungi and bacterial pathogens at this early stage of invasion, which needs to be further study.

Facing pathogen invasion, plant have evolved a complex signal transduction network to activate plant resistance. Ca^2+^ is a conserved second messenger involved in nearly all aspects of cellular signaling programs including regulation of plant development as well as stress resistance (Zhang et al., 2014). CDPK senses Ca^2+^ signals and translates them into protein phosphorylation (Liese and Romeis, 2013). In our study, several of genes encoding CDPKs were identified to differently express after *C. fulvum* invasion, either upregulated or downregulated, which are in consistence with many previous reports that CDPKs are widely involved in the regulation of different kinds of disease resistance (Boudsocq et al., 2010; Boudsocq and Sheen, 2013; Romeis and Herde, 2014). For example, the *AtCDPK4/5/6/11* phosphorylate specific WRKY transcription factor regulates immune response by restricting pathogen growth (Gao et al., 2013). The *StCDPK4/5* mediated ROS production by phosphorylating NADPH oxidases (RBOH, Respiratory Burst Oxidase Homolog) in potato (Kobayashi et al., 2007). In our study, *RBOH1* was also upregulated and ROS was produced after *C. fulvum* invasion, suggesting that CDPK may have activated ROS production and that Ca^2+^ signal pathway could play an essential role in plant early defense against *C. fulvum*.

More and more evidence demonstrate that multiple hormones are involved in plant and pathogen interactions (Denance et al., 2013; Li et al., 2019a; Liu et al., 2021). In our study, results showed that genes involved in SA signaling pathway, such as SA receptor *NPR1*, transcription factor *TGA* and *PR1*, were all upregulated. Previous reports showed that SA signaling pathway may be a unique *Cf12* dependent resistance pathway (Xue et al., 2017). When the biotrophic pathogen *P. syringae* infects *Arabidopsis*, plants also activate SA dependent defenses against invasion (DebRoy et al., 2004; Geng et al., 2012). Our results provide further evidence for the important role of SA signaling pathway in plant defense against biotroph pathogens. A few genes involved in SA signaling pathway were downregulated expression such as *BOP2*, a NPR1 like gene, and the function for BOP2 in plant is to control growth asymmetry (Hepworth et al., 2005). Therefore, we also speculate that SA may regulate the balance between cell growth and cell death, but the significance on how to regulate plant development is unclear (Hepworth et al., 2005). It is well known that SA and JA-mediate defense singling pathways have antagonistic effect during pathogen invasion (Thaler et al., 2012). In our study, we also found that genes involved in JA synthesis were downregulated after *C. fulvum* invasion. With the measurement of SA and JA contents after fungal invasion, our results revealed that SA accumulated significantly and the JA level decreased significantly upon *C. fulvum* invasion, indicating the existence of crosstalk between SA and JA signaling pathway at the early stage of invasion. In addition, our results revealed that *C. fulvum* invasion also activated the ABA signaling pathway, which may be induced to trigger the ABA-dependent stomata immune response against pathogen entry (Ou et al., 2022). There are also many DEGs involved in the auxin signaling pathway. Given the tradeoff between development and plant defense, it is not surprised that DEGs in so many pathways were identified after *C. fulvum* invasion.

Plant metabolism can be divided into primary and secondary metabolism, and among the secondary metabolism, the plant phenylpropanoid pathway is one of the most important secondary metabolic pathways (Li et al., 2007). Previous studies have found that the expression of genes related to the phenylpropanoid pathway is also stimulated by Asian soybean rust in *Arabidopsis* (Beyer et al., 2019), suggesting that the phenylpropanoid pathway plays an important role in plant response to pathogen infection (Dong and Lin, 2021). It has been reported that the expression of *PAL* and *4CL*, key genes in the phenylpropanoid pathway, were upregulated both in susceptible and resistant varieties upon *C. fulvum* invasion (Xue et al., 2017), which is in consistence with our results. In this study, *ANS* as the key gene for anthocyanin synthesis (Mattus-Araya et al., 2022), was downregulated, indicating the inhibition of anthocyanin synthesis in plants in response to *C. fulvum.* Interestingly, *CCR2* and some genes encoding peroxidases involved in lignin synthesis pathway were upregulated (**Table 8**); we hypothesized that tomato plants might activate the lignin synthesis pathway to synthesize lignin as a physical barrier to prevent pathogen invasion.

## Conclusion

The plant pathogen *C. fulvum* causes a large amount of yield loss in global tomato production, which can cause more than 50% reduction in tomato production in severe cases. In the present study, we carried out RNA-seq experiments to identify DEGs induced in *C. fulvum* inoculated tomato leaves. We analyzed DEGs related to plant and pathogen interaction pathway, plant hormones signaling pathway and plant phenylpropanoid pathway. Our results discovered that a number of core defense genes against fungal invasion were induced. Moreover, we found SA and ABA accumulation in tomato leaves after *C. fulvum* invasion, while JA content decreased after *C. fulvum* invasion. Together, our results will broaden our understanding for investigating the mechanism of *C. fulvum* and tomato interaction in future.

## Data availability statement

The data presented in the study are deposited in the NCBI gene expression omnibus server (https://www.ncbi.nlm.nih.gov/geo/), accession number SRR21437044, SRR21437045, SRR21437046, SRR21437047, SRR21437048, SRR21437049.

## Author contributions

Conceptualization, RP and SS; Formal analysis, RP, SS and LK; Funding acquisition, SS, ZC and XG; Methodology, PW, ZC, NL, LX and XG; Supervision, SS and XG; Writing – original draft, SS, XG and RP; Writing review & editing, HW and XG. All authors contributed to the article and approved the submitted version.
