# Supplementary material for: Physiological and transcriptome profiling revealed defense networks during Cladosporium fulvum and tomato interaction at the early stage
Source: Front Plant Sci. 2022 Dec 6;13:1085395. doi: 10.3389/fpls.2022.1085395 (PMC9763619; doi:10.3389/fpls.2022.1085395)
Supplement: Supplementary file 1 [file DataSheet_1.docx]

**Table S1.** **Differentially expressed genes upregulates by** ***Cladosporium fulvum* treatment**

| **Gene ID** | **Gene Symbol** | **Log_2_**  **(*C.fulvum*/ Control)** | ***P*-value**  **(*C.fulvum*/ Control)** |
| --- | --- | --- | --- |
| 100037494 | PHOT2 | 1.768182326 | 4.87E-04 |
| 100037510 | SRK2C | 1.206668058 | 0.003101157 |
| 100037515 | DNAJ | 2.893575424 | 2.88E-04 |
| 100037731 | LOC100037731 | 1.181357322 | 0.007990219 |
| 100125906 | ODD | 3.069241709 | 1.05E-05 |
| 100125909 | ACO6 | 1.876872534 | 7.10E-06 |
| 100134889 | GA2ox4 | 3.794984072 | 5.19E-09 |
| 100134914 | HSP70 | 2.690315993 | 2.29E-06 |
| 100135703 | TPS1 | 3.320658667 | 1.64E-08 |
| 100147721 | LOC100147721 | 2.941297061 | 0.002618669 |
| 100147727 | Ca3 | 6.96322244 | 0.00774881 |
| 100147728 | TBP1 | 1.41896594 | 4.80E-06 |
| 100191111 | LOC100191111 | 6.508305302 | 2.22E-09 |
| 100301931 | GR | 1.101365686 | 0.001316412 |
| 100316872 | LOC100316872 | 3.415085634 | 8.79E-05 |
| 100316875 | MIOX | 4.936702831 | 0.007697612 |
| 100316880 | LOC100316880 | 4.718233993 | 6.38E-10 |
| 100316887 | LOC100316887 | 1.923389957 | 2.52E-06 |
| 100498663 | ARF19A | 1.670411771 | 3.19E-05 |
| 100529100 | TTS2 | 1.513902524 | 3.27E-04 |
| 100529106 | PIP2-4 | 7.164653126 | 3.85E-11 |
| 100529138 | WRKY72a | 3.489788942 | 2.16E-04 |
| 100620057 | AP2b | 1.084111955 | 0.001788765 |
| 100736245 | ACA10 | 1.239809386 | 7.00E-06 |
| 100736433 | Hsc70.1 | 3.420225861 | 0.00526887 |
| 100736441 | LOC100736441 | 3.519934883 | 2.22E-06 |
| 100736451 | BAM1 | 1.715528112 | 0.002200482 |
| 100736454 | TCP6 | 1.624392491 | 0.001149628 |
| 100736456 | TCP10 | 1.856094692 | 1.73E-05 |
| 100736458 | TCP16 | 1.756650611 | 0.00315492 |
| 100736498 | PSK1 | 2.074185992 | 2.24E-07 |
| 100736503 | TIL | 1.955751253 | 1.45E-04 |
| 100736509 | ARF1 | 1.171731864 | 0.00645183 |
| 100736518 | BRC1a | 2.016551759 | 0.001239894 |
| 100736519 | TCP12 | 2.367575477 | 0.005143405 |
| 100736526 | DMR6-1 | 1.421474638 | 3.01E-05 |
| 100736533 | LOC100736533 | 1.843088962 | 2.34E-04 |
| 100736538 | LOC100736538 | 1.386324969 | 4.03E-05 |
| 100736548 | MAPK14 | 1.042221805 | 3.33E-05 |
| 100736549 | TCP13 | 1.522252988 | 0.004628123 |
| 100750250 | LOC100750250 | 2.641010012 | 0.003140792 |
| 100750254 | XIP1-1-beta | 1.326808658 | 5.32E-04 |
| 100874535 | CKX5 | 2.031161857 | 2.35E-04 |
| 100874537 | CKX2 | 2.196911209 | 0.005695901 |
| 100874540 | LOG8 | 1.9733044 | 7.16E-04 |
| 101055500 | CIPK6 | 1.284182187 | 2.88E-04 |
| 101055503 | CIPK2 | 1.743083851 | 9.32E-05 |
| 101055519 | LOC101055519 | 1.728217368 | 9.56E-07 |
| 101055527 | LOC101055527 | 1.450204071 | 9.70E-04 |
| 101055534 | LOC101055534 | 2.24243643 | 0.009326309 |
| 101055555 | LOC101055555 | 1.799555133 | 0.009160947 |
| 101055558 | Arf/Xyl1 | 2.109557832 | 3.69E-05 |
| 101055572 | GGP1 | 3.617180797 | 2.68E-11 |
| 101055583 | LOC101055583 | 3.183568267 | 2.27E-12 |
| 101055587 | GLK1 | 2.084756824 | 2.33E-05 |
| 101055596 | Hsc70.3 | 4.865417656 | 3.43E-07 |
| 101055612 | CKX4 | 1.764778343 | 0.003679057 |
| 101055613 | GLK2 | 3.313136131 | 1.23E-04 |
| 101243626 | LOC101243626 | 2.577931643 | 3.42E-08 |
| 101243639 | LOC101243639 | 1.049820373 | 0.003886845 |
| 101243660 | LOC101243660 | 1.331912215 | 0.004668823 |
| 101243689 | LOC101243689 | 1.416648591 | 8.81E-04 |
| 101243692 | LOC101243692 | 1.337531931 | 0.004536656 |
| 101243702 | LOC101243702 | 1.890554595 | 0.00119484 |
| 101243709 | LOC101243709 | 2.602749931 | 0.006872732 |
| 101243720 | LOC101243720 | 3.025188021 | 3.08E-09 |
| 101243731 | LOC101243731 | 7.910245396 | 1.43E-19 |
| 101243744 | LOC101243744 | 1.215973638 | 0.001072471 |
| 101243814 | LOC101243814 | 2.873814603 | 2.37E-07 |
| 101243822 | FBA4 | 1.415223885 | 7.17E-04 |
| 101243840 | LOC101243840 | 1.197995422 | 4.11E-04 |
| 101243841 | LOC101243841 | 1.893240119 | 0.00333655 |
| 101243845 | LOC101243845 | 4.833340212 | 0.002863473 |
| 101243846 | TFT1 | 1.818419057 | 0.004844265 |
| 101243862 | LOC101243862 | 1.804999673 | 3.71E-05 |
| 101243876 | LOC101243876 | 1.661673551 | 0.003954811 |
| 101243889 | LOC101243889 | 3.000415433 | 1.73E-04 |
| 101243890 | LOC101243890 | 1.266352915 | 0.002420032 |
| 101243894 | LOC101243894 | 2.731502764 | 8.11E-17 |
| 101243919 | LOC101243919 | 1.8008134 | 0.008778683 |
| 101243932 | LOC101243932 | 2.239144507 | 6.01E-04 |
| 101243956 | LOC101243956 | 7.206711186 | 2.82E-15 |
| 101243961 | LOC101243961 | 6.175394508 | 8.82E-04 |
| 101243977 | LOC101243977 | 4.107787927 | 3.26E-04 |
| 101243985 | LOC101243985 | 2.328245016 | 0.002482602 |
| 101244029 | LOC101244029 | 2.03883392 | 8.74E-05 |
| 101244047 | LOC101244047 | 2.480787044 | 2.77E-06 |
| 101244074 | LOC101244074 | 1.269070682 | 0.005752121 |
| 101244075 | LOC101244075 | 2.372389169 | 2.34E-04 |
| 101244080 | LOC101244080 | 1.87251374 | 6.06E-06 |
| 101244106 | LOC101244106 | 1.112483944 | 0.00655195 |
| 101244132 | LOC101244132 | 1.133600488 | 8.90E-05 |
| 101244135 | LOC101244135 | 2.580367222 | 2.11E-09 |
| 101244136 | LOC101244136 | 4.131531138 | 8.35E-27 |
| 101244140 | LOC101244140 | 2.057792601 | 2.43E-04 |
| 101244161 | LOC101244161 | 1.72256875 | 0.003181389 |
| 101244170 | LOC101244170 | 1.01255176 | 0.001671306 |
| 101244175 | LOC101244175 | 5.371145828 | 8.29E-04 |
| 101244177 | LOC101244177 | 2.208292581 | 1.89E-04 |
| 101244185 | LOC101244185 | 1.884276229 | 5.24E-08 |
| 101244231 | LOC101244231 | 1.582200974 | 3.88E-07 |
| 101244237 | LOC101244237 | 1.138623326 | 0.001459083 |
| 101244253 | LOC101244253 | 3.371698274 | 2.91E-06 |
| 101244281 | LOC101244281 | 3.19377269 | 0.009086812 |
| 101244290 | LOC101244290 | 3.15164138 | 1.68E-09 |
| 101244297 | LOC101244297 | 1.248905941 | 0.005263914 |
| 101244300 | LOC101244300 | 7.184513234 | 6.52E-12 |
| 101244313 | LOC101244313 | 1.751423696 | 4.69E-04 |
| 101244324 | LOC101244324 | 1.778881197 | 1.53E-04 |
| 101244325 | LOC101244325 | 2.580423174 | 6.06E-06 |
| 101244339 | LOC101244339 | 3.907580819 | 5.35E-04 |
| 101244359 | LOC101244359 | 1.021809568 | 6.85E-04 |
| 101244390 | LOC101244390 | 5.416711703 | 3.37E-05 |
| 101244399 | LOC101244399 | 3.529228825 | 2.25E-17 |
| 101244417 | LOC101244417 | 1.392103678 | 4.23E-04 |
| 101244433 | LOC101244433 | 1.919916085 | 3.29E-08 |
| 101244441 | LOC101244441 | 2.089319301 | 0.008732875 |
| 101244457 | LOC101244457 | 1.213886258 | 0.007916072 |
| 101244460 | LOC101244460 | 1.433749775 | 0.001836012 |
| 101244466 | LOC101244466 | 1.100088757 | 0.001679609 |
| 101244471 | LOC101244471 | 1.577477268 | 0.008128765 |
| 101244480 | LOC101244480 | 1.366764178 | 0.00137059 |
| 101244496 | LOC101244496 | 1.379787866 | 0.001322034 |
| 101244499 | LOC101244499 | 1.460790525 | 5.60E-04 |
| 101244513 | LOC101244513 | 4.513849468 | 0.007753079 |
| 101244523 | LOC101244523 | 1.813132186 | 0.007603975 |
| 101244528 | LOC101244528 | 3.524237882 | 5.47E-09 |
| 101244540 | LOC101244540 | 1.655287981 | 0.002959488 |
| 101244562 | LOC101244562 | 7.708229824 | 5.22E-11 |
| 101244598 | LOC101244598 | 2.659376221 | 4.65E-09 |
| 101244627 | LOC101244627 | 5.751477004 | 0.002778331 |
| 101244637 | LOC101244637 | 2.079880766 | 1.63E-05 |
| 101244652 | LOC101244652 | 5.4808082 | 1.75E-09 |
| 101244665 | LOC101244665 | 2.802604693 | 0.005427964 |
| 101244669 | LOC101244669 | 4.72756144 | 1.11E-06 |
| 101244686 | LOC101244686 | 3.488193857 | 1.53E-04 |
| 101244692 | LOC101244692 | 1.534523665 | 1.29E-04 |
| 101244716 | LOC101244716 | 3.74457893 | 3.34E-09 |
| 101244735 | LOC101244735 | 2.139856698 | 0.002836565 |
| 101244746 | LOC101244746 | 5.024072075 | 0.00933853 |
| 101244778 | SEU3 | 1.040058995 | 0.003213798 |
| 101244789 | LOC101244789 | 3.436192022 | 1.42E-05 |
| 101244811 | LOC101244811 | 1.500412455 | 1.03E-07 |
| 101244867 | LOC101244867 | 3.363999532 | 0.001907363 |
| 101244886 | LOC101244886 | 2.181737601 | 7.73E-04 |
| 101244909 | LNK1 | 2.691004819 | 1.87E-05 |
| 101244918 | LOC101244918 | 2.387915929 | 5.84E-06 |
| 101244953 | PHO1-3 | 1.710233151 | 4.13E-04 |
| 101244961 | LOC101244961 | 5.356353215 | 4.94E-11 |
| 101244974 | LOC101244974 | 1.96400631 | 0.005259016 |
| 101244988 | LOC101244988 | 1.36976806 | 0.004257456 |
| 101244989 | LOC101244989 | 1.894859682 | 0.001880766 |
| 101244994 | LOC101244994 | 1.438782642 | 0.005266182 |
| 101244995 | LOC101244995 | 1.564302758 | 0.003473936 |
| 101244997 | LOC101244997 | 1.004438854 | 0.006848975 |
| 101245027 | LOC101245027 | 3.439387265 | 5.51E-04 |
| 101245032 | LOC101245032 | 1.18824649 | 0.002678119 |
| 101245047 | LOC101245047 | 1.812521756 | 4.77E-05 |
| 101245071 | LOC101245071 | 1.155015688 | 3.30E-04 |
| 101245092 | LOC101245092 | 1.572262372 | 0.0013477 |
| 101245101 | LOC101245101 | 4.626966554 | 0.005491605 |
| 101245159 | LOC101245159 | 2.584911958 | 1.74E-04 |
| 101245195 | LOC101245195 | 3.535614165 | 2.58E-04 |
| 101245208 | LOC101245208 | 4.947792391 | 3.70E-05 |
| 101245227 | LOC101245227 | 1.095765229 | 0.002316559 |
| 101245290 | LOC101245290 | 1.33549883 | 2.83E-04 |
| 101245298 | LOC101245298 | 4.775799173 | 4.67E-06 |
| 101245307 | LOC101245307 | 1.333557334 | 0.001037214 |
| 101245316 | TAP2 | 3.700131 | 5.34E-04 |
| 101245324 | LOC101245324 | 3.174050841 | 0.002375367 |
| 101245329 | LOC101245329 | 7.611666192 | 3.76E-15 |
| 101245341 | LOC101245341 | 1.134802303 | 6.15E-04 |
| 101245348 | LOC101245348 | 8.850636318 | 3.36E-07 |
| 101245387 | LOC101245387 | 1.731713058 | 0.001697937 |
| 101245390 | LOC101245390 | 1.233664008 | 3.01E-05 |
| 101245410 | LOC101245410 | 5.603131127 | 9.27E-05 |
| 101245411 | LOC101245411 | 1.163673703 | 3.19E-04 |
| 101245415 | CO3 | 1.38694109 | 0.005536433 |
| 101245425 | LOC101245425 | 1.576159605 | 0.004408068 |
| 101245475 | LOC101245475 | 1.273099079 | 9.73E-04 |
| 101245487 | LOC101245487 | 2.188579702 | 0.001547198 |
| 101245498 | LOC101245498 | 1.354761779 | 9.84E-06 |
| 101245518 | LOC101245518 | 3.306796684 | 4.15E-10 |
| 101245519 | LOC101245519 | 5.104326916 | 4.84E-20 |
| 101245539 | LOC101245539 | 1.242107407 | 0.00819727 |
| 101245588 | LOC101245588 | 1.901673397 | 1.08E-05 |
| 101245604 | LOC101245604 | 1.229935302 | 0.001283296 |
| 101245605 | LOC101245605 | 1.31159259 | 0.008202919 |
| 101245606 | LOC101245606 | 1.973757465 | 1.19E-04 |
| 101245612 | LOC101245612 | 1.574868431 | 0.003932468 |
| 101245621 | LOC101245621 | 1.708978122 | 0.005196235 |
| 101245623 | LOC101245623 | 4.808384921 | 0.001082848 |
| 101245626 | LOC101245626 | 2.615674685 | 0.002017391 |
| 101245647 | LOC101245647 | 1.939719047 | 9.57E-04 |
| 101245683 | LOC101245683 | 1.537829658 | 9.37E-04 |
| 101245689 | LOC101245689 | 1.936123206 | 0.00526269 |
| 101245695 | LOC101245695 | 1.620211362 | 7.61E-04 |
| 101245696 | LOC101245696 | 2.866004793 | 3.57E-06 |
| 101245699 | LOC101245699 | 10.50548922 | 2.65E-11 |
| 101245716 | LOC101245716 | 1.562595271 | 0.001847829 |
| 101245731 | ABCI10 | 2.588712542 | 8.74E-04 |
| 101245763 | LOC101245763 | 1.371232099 | 3.64E-05 |
| 101245795 | LOC101245795 | 3.996490827 | 2.40E-34 |
| 101245842 | LOC101245842 | 1.401692468 | 6.16E-04 |
| 101245854 | LOC101245854 | 2.405964719 | 4.04E-10 |
| 101245896 | LOC101245896 | 2.025523379 | 1.26E-07 |
| 101245903 | LOC101245903 | 1.150204725 | 8.73E-04 |
| 101245915 | LOC101245915 | 1.393918772 | 0.003209387 |
| 101245917 | LOC101245917 | 1.454170732 | 0.003295551 |
| 101245922 | LOC101245922 | 1.555639922 | 1.37E-05 |
| 101245930 | LOC101245930 | 4.057094124 | 5.46E-09 |
| 101245934 | LOC101245934 | 2.580503262 | 1.24E-05 |
| 101245940 | LOC101245940 | 2.967283562 | 2.86E-07 |
| 101245943 | LOC101245943 | 1.139255438 | 0.001107344 |
| 101245952 | LOC101245952 | 1.790423392 | 7.70E-04 |
| 101245986 | LOC101245986 | 1.042962648 | 0.004196917 |
| 101246004 | LOC101246004 | 2.108091613 | 1.26E-04 |
| 101246022 | LOC101246022 | 1.004825354 | 0.00281268 |
| 101246027 | LOC101246027 | 1.363508572 | 1.24E-04 |
| 101246032 | LOC101246032 | 1.5305368 | 2.18E-05 |
| 101246041 | LOC101246041 | 2.601444524 | 3.25E-06 |
| 101246079 | LOC101246079 | 1.73115907 | 4.67E-04 |
| 101246105 | LOC101246105 | 4.013078365 | 4.79E-08 |
| 101246106 | LOC101246106 | 7.38826189 | 1.25E-13 |
| 101246110 | LOC101246110 | 2.2017908 | 1.97E-04 |
| 101246122 | LOC101246122 | 5.544469706 | 0.001713517 |
| 101246136 | LOC101246136 | 1.066817017 | 0.002569246 |
| 101246149 | LOC101246149 | 3.294255623 | 9.97E-07 |
| 101246163 | LOC101246163 | 1.526807573 | 0.008834093 |
| 101246187 | LOC101246187 | 1.091203789 | 0.005673719 |
| 101246202 | LOC101246202 | 1.531051292 | 7.37E-05 |
| 101246226 | LOC101246226 | 2.018934002 | 2.33E-04 |
| 101246260 | LOC101246260 | 1.569636024 | 8.61E-04 |
| 101246264 | LOC101246264 | 2.4435133 | 0.005174941 |
| 101246280 | LOC101246280 | 3.58957915 | 1.73E-04 |
| 101246288 | LOC101246288 | 3.125685562 | 0.004572738 |
| 101246296 | LOC101246296 | 1.888472966 | 0.005133455 |
| 101246302 | LOC101246302 | 1.36880843 | 9.58E-04 |
| 101246306 | LOC101246306 | 2.32801261 | 8.42E-08 |
| 101246317 | LOC101246317 | 1.173779478 | 7.22E-04 |
| 101246326 | SFP6 | 1.047666223 | 6.58E-04 |
| 101246353 | LOC101246353 | 1.259860404 | 0.008149939 |
| 101246381 | LOC101246381 | 5.394935813 | 3.51E-19 |
| 101246393 | UGT76E1 | 7.524063775 | 7.18E-08 |
| 101246398 | LOC101246398 | 7.74161307 | 8.70E-05 |
| 101246404 | LOC101246404 | 3.404372038 | 0.008580293 |
| 101246450 | LOC101246450 | 1.070388589 | 3.98E-04 |
| 101246461 | LOC101246461 | 2.208722862 | 6.33E-04 |
| 101246473 | LOC101246473 | 2.477411191 | 0.005831412 |
| 101246519 | LOC101246519 | 2.086927338 | 8.39E-06 |
| 101246521 | LOC101246521 | 3.999746262 | 2.47E-04 |
| 101246548 | LOC101246548 | 2.810123858 | 0.008121622 |
| 101246586 | LOC101246586 | 3.192390072 | 5.85E-05 |
| 101246590 | LOC101246590 | 4.7316996 | 1.18E-19 |
| 101246666 | LOC101246666 | 8.775552607 | 3.04E-15 |
| 101246670 | ABCG3 | 4.217254547 | 2.47E-05 |
| 101246683 | LOC101246683 | 1.376348942 | 5.46E-05 |
| 101246686 | LOC101246686 | 2.054574689 | 0.00228529 |
| 101246690 | LOC101246690 | 3.230672551 | 0.001847796 |
| 101246694 | LOC101246694 | 1.988470617 | 1.42E-05 |
| 101246695 | LOC101246695 | 1.286104706 | 0.004520675 |
| 101246714 | LOC101246714 | 2.171054469 | 0.001308364 |
| 101246719 | LOC101246719 | 1.63249077 | 0.001367551 |
| 101246721 | LOC101246721 | 1.51728292 | 1.28E-04 |
| 101246747 | LOC101246747 | 1.295587834 | 1.14E-04 |
| 101246751 | LOC101246751 | 1.995520485 | 4.73E-06 |
| 101246755 | LOC101246755 | 4.508099554 | 4.01E-05 |
| 101246761 | LOC101246761 | 2.233094855 | 0.002941548 |
| 101246763 | LOC101246763 | 3.616165255 | 3.20E-06 |
| 101246767 | LOC101246767 | 1.746378712 | 4.36E-04 |
| 101246797 | LOC101246797 | 1.00258788 | 9.06E-04 |
| 101246806 | LOC101246806 | 3.268580249 | 3.77E-04 |
| 101246807 | LOC101246807 | 1.057093256 | 0.008230946 |
| 101246865 | LOC101246865 | 2.155354831 | 0.002325409 |
| 101246870 | FBA1 | 3.042825202 | 6.26E-09 |
| 101246876 | LOC101246876 | 2.749390179 | 0.003222039 |
| 101246942 | LOC101246942 | 1.87476746 | 5.18E-08 |
| 101246954 | LOC101246954 | 4.62126759 | 3.52E-17 |
| 101246977 | LOC101246977 | 1.434247189 | 2.73E-04 |
| 101246981 | LOC101246981 | 1.34734564 | 0.002221895 |
| 101246982 | LOC101246982 | 1.049957087 | 0.008735471 |
| 101246984 | LOC101246984 | 1.913975363 | 9.84E-04 |
| 101247013 | LOC101247013 | 5.611834818 | 7.01E-06 |
| 101247036 | LOC101247036 | 2.415942731 | 1.20E-05 |
| 101247047 | LOC101247047 | 1.468665176 | 0.009224773 |
| 101247071 | LOC101247071 | 1.078830398 | 0.004449304 |
| 101247075 | LOC101247075 | 2.53256297 | 1.37E-08 |
| 101247078 | LOC101247078 | 4.842101578 | 0.004565581 |
| 101247086 | LOC101247086 | 1.052150823 | 0.008059301 |
| 101247100 | LOC101247100 | 6.304130364 | 2.95E-09 |
| 101247147 | LOC101247147 | 1.249614759 | 0.00110212 |
| 101247149 | LOC101247149 | 3.091139721 | 2.97E-11 |
| 101247180 | LOC101247180 | 5.898564645 | 7.49E-06 |
| 101247183 | LOC101247183 | 1.737288184 | 0.001463382 |
| 101247212 | ASR3 | 7.596673588 | 2.77E-05 |
| 101247301 | LOC101247301 | 6.72274485 | 1.12E-05 |
| 101247308 | LOC101247308 | 2.584302423 | 0.007368753 |
| 101247353 | LOC101247353 | 6.075397123 | 1.69E-04 |
| 101247356 | LOC101247356 | 2.338663064 | 1.47E-04 |
| 101247385 | LOC101247385 | 1.859276284 | 4.05E-05 |
| 101247386 | LOC101247386 | 2.185318452 | 8.69E-07 |
| 101247393 | LOC101247393 | 1.193871492 | 8.36E-04 |
| 101247444 | LOC101247444 | 1.885681148 | 7.55E-04 |
| 101247497 | LOC101247497 | 1.677457775 | 2.80E-05 |
| 101247540 | LOC101247540 | 4.343125017 | 8.14E-05 |
| 101247541 | LOC101247541 | 1.756055831 | 0.001006186 |
| 101247545 | ABCG44 | 2.434097348 | 6.06E-04 |
| 101247561 | LOC101247561 | 1.376196661 | 0.007286644 |
| 101247622 | LOC101247622 | 2.387630488 | 1.75E-06 |
| 101247627 | LOC101247627 | 1.393888253 | 0.004727278 |
| 101247644 | LOC101247644 | 1.694237484 | 0.008260831 |
| 101247647 | LOC101247647 | 6.20856325 | 1.05E-13 |
| 101247650 | Hcr9-0 | 1.977563089 | 0.001381268 |
| 101247679 | DCL2c | 3.186915512 | 8.70E-05 |
| 101247686 | LOC101247686 | 2.689819991 | 8.16E-06 |
| 101247687 | LOC101247687 | 1.486168175 | 0.002073177 |
| 101247689 | LOC101247689 | 3.72373694 | 0.001464176 |
| 101247705 | LOC101247705 | 5.98521126 | 2.15E-16 |
| 101247706 | LOC101247706 | 1.887859882 | 8.89E-04 |
| 101247736 | LOC101247736 | 1.753047177 | 1.93E-04 |
| 101247747 | PIP2-1 | 2.656877272 | 4.53E-04 |
| 101247753 | LOC101247753 | 1.60994847 | 2.99E-04 |
| 101247755 | LOC101247755 | 1.537212783 | 0.001777445 |
| 101247766 | LOC101247766 | 1.332013455 | 0.003711104 |
| 101247772 | Hsc70.2 | 1.560099151 | 0.001866282 |
| 101247787 | LOC101247787 | 1.817727805 | 5.71E-05 |
| 101247790 | LOC101247790 | 1.619594641 | 3.21E-04 |
| 101247791 | LOC101247791 | 4.18839713 | 2.98E-05 |
| 101247803 | LOC101247803 | 1.342812873 | 0.005261545 |
| 101247804 | LOC101247804 | 6.102852604 | 1.22E-24 |
| 101247814 | LOC101247814 | 6.939961553 | 3.13E-07 |
| 101247819 | LOC101247819 | 2.005227647 | 0.002604084 |
| 101247825 | LOC101247825 | 4.317387565 | 2.80E-08 |
| 101247864 | LOC101247864 | 1.505118001 | 1.70E-04 |
| 101247873 | LOC101247873 | 5.053040252 | 0.001891874 |
| 101247877 | LOC101247877 | 2.83369452 | 5.00E-04 |
| 101247891 | LOC101247891 | 1.424082451 | 0.004537694 |
| 101247908 | LOC101247908 | 1.165310757 | 1.61E-04 |
| 101247909 | LOC101247909 | 1.721626015 | 7.06E-06 |
| 101247959 | LOC101247959 | 3.034110419 | 0.003557148 |
| 101247965 | LOC101247965 | 1.354601524 | 0.003321143 |
| 101247974 | LOC101247974 | 1.595442566 | 0.001116639 |
| 101247976 | DCL2d | 4.238086211 | 5.22E-10 |
| 101247994 | LOC101247994 | 4.077967308 | 1.88E-05 |
| 101248003 | LOC101248003 | 1.181595503 | 0.003427034 |
| 101248009 | LOC101248009 | 7.581937379 | 5.96E-34 |
| 101248052 | LOC101248052 | 1.24078447 | 0.00808098 |
| 101248072 | LOC101248072 | 7.152320181 | 0.001668361 |
| 101248073 | LOC101248073 | 1.732073898 | 2.50E-04 |
| 101248095 | LOC101248095 | 4.755256668 | 1.80E-08 |
| 101248108 | LOC101248108 | 1.718582335 | 3.79E-04 |
| 101248118 | LOC101248118 | 3.648893242 | 5.57E-04 |
| 101248153 | LOC101248153 | 1.039466341 | 0.001221722 |
| 101248154 | LOC101248154 | 1.286567892 | 0.004472352 |
| 101248161 | LOC101248161 | 2.550980379 | 1.35E-04 |
| 101248184 | LOC101248184 | 1.506598971 | 1.88E-05 |
| 101248189 | LOC101248189 | 2.453604435 | 7.44E-04 |
| 101248208 | LOC101248208 | 1.386833208 | 0.0023073 |
| 101248210 | LOC101248210 | 2.290156352 | 1.37E-06 |
| 101248219 | LOC101248219 | 5.850654563 | 0.002595551 |
| 101248237 | LOC101248237 | 6.340610335 | 1.03E-11 |
| 101248247 | LOC101248247 | 2.846442186 | 6.84E-04 |
| 101248252 | LOC101248252 | 1.463995316 | 6.88E-06 |
| 101248253 | LOC101248253 | 3.676383826 | 0.001337378 |
| 101248296 | LOC101248296 | 2.398870866 | 9.97E-09 |
| 101248314 | LOC101248314 | 1.110527595 | 0.002987469 |
| 101248324 | LOC101248324 | 6.246835852 | 4.63E-10 |
| 101248348 | LOC101248348 | 1.000847279 | 0.004264138 |
| 101248373 | LOC101248373 | 5.635145225 | 6.55E-17 |
| 101248374 | LOC101248374 | 1.807435157 | 0.002929572 |
| 101248378 | LOC101248378 | 3.012057151 | 3.64E-08 |
| 101248395 | LOC101248395 | 1.509719001 | 4.56E-04 |
| 101248432 | LOC101248432 | 3.919245556 | 2.92E-04 |
| 101248446 | LOC101248446 | 3.104066583 | 5.81E-14 |
| 101248465 | LOC101248465 | 1.405809911 | 0.005096842 |
| 101248483 | LOC101248483 | 1.745883967 | 0.001426332 |
| 101248484 | LOC101248484 | 1.721719763 | 0.002036541 |
| 101248506 | LOC101248506 | 1.189608189 | 2.49E-05 |
| 101248523 | LOC101248523 | 3.408462729 | 7.46E-04 |
| 101248552 | LOC101248552 | 1.540296346 | 4.19E-04 |
| 101248561 | LOC101248561 | 3.18146953 | 5.11E-04 |
| 101248575 | LOC101248575 | 1.659816123 | 4.27E-05 |
| 101248590 | LOC101248590 | 5.021724676 | 1.45E-05 |
| 101248595 | LOC101248595 | 2.077119463 | 1.01E-08 |
| 101248614 | LOC101248614 | 1.298000898 | 6.89E-06 |
| 101248623 | LOC101248623 | 1.021811053 | 0.001449721 |
| 101248631 | LOC101248631 | 2.595333539 | 8.53E-04 |
| 101248651 | LOC101248651 | 1.415503052 | 0.005570453 |
| 101248657 | LOC101248657 | 7.073311163 | 2.95E-08 |
| 101248665 | NAP2 | 7.684485148 | 6.36E-38 |
| 101248674 | LOC101248674 | 1.476265281 | 1.10E-05 |
| 101248692 | LOC101248692 | 3.113055055 | 2.52E-10 |
| 101248742 | LOC101248742 | 1.458069135 | 5.63E-05 |
| 101248757 | LOC101248757 | 1.534085075 | 0.002726046 |
| 101248769 | LOC101248769 | 2.020875906 | 1.74E-05 |
| 101248774 | LOC101248774 | 1.130145653 | 0.005517541 |
| 101248786 | LOC101248786 | 2.048068368 | 5.72E-06 |
| 101248790 | LOC101248790 | 1.99693429 | 3.46E-05 |
| 101248812 | LOC101248812 | 5.309614521 | 1.10E-07 |
| 101248835 | LOC101248835 | 1.493056047 | 9.27E-04 |
| 101248856 | LOC101248856 | 1.040151995 | 0.00697976 |
| 101248857 | LOC101248857 | 1.198272231 | 0.007679749 |
| 101248865 | ABCA2 | 1.605906049 | 0.005840554 |
| 101248920 | LOC101248920 | 1.486004634 | 5.46E-07 |
| 101248986 | LOC101248986 | 1.665762545 | 3.05E-05 |
| 101248987 | LOC101248987 | 1.496088803 | 2.15E-05 |
| 101249038 | LOC101249038 | 5.804924905 | 0.003691154 |
| 101249045 | LOC101249045 | 4.452361863 | 7.25E-07 |
| 101249060 | LOC101249060 | 2.138266109 | 0.001220702 |
| 101249062 | LOC101249062 | 3.358865412 | 6.37E-08 |
| 101249087 | LOC101249087 | 1.211071856 | 0.003312928 |
| 101249101 | LOC101249101 | 1.305682438 | 0.001270456 |
| 101249136 | LOC101249136 | 1.480345419 | 0.004485509 |
| 101249149 | LOC101249149 | 5.057169869 | 3.46E-05 |
| 101249158 | LOC101249158 | 1.734526452 | 0.001428562 |
| 101249199 | LOC101249199 | 1.418308381 | 0.004279281 |
| 101249201 | LOC101249201 | 3.478943175 | 0.002872682 |
| 101249208 | ABCC9 | 2.065818387 | 1.01E-07 |
| 101249209 | LOC101249209 | 1.255303886 | 0.003246597 |
| 101249223 | LOC101249223 | 1.042757338 | 0.001450284 |
| 101249263 | LOC101249263 | 1.695650691 | 3.56E-04 |
| 101249264 | LOC101249264 | 1.579218374 | 0.001407209 |
| 101249294 | LOC101249294 | 2.126178855 | 0.006915282 |
| 101249296 | LOC101249296 | 3.871740308 | 5.05E-04 |
| 101249315 | LOC101249315 | 1.164981622 | 4.56E-04 |
| 101249322 | LOC101249322 | 1.647221722 | 5.85E-05 |
| 101249325 | LOC101249325 | 2.732092285 | 8.04E-11 |
| 101249340 | LOC101249340 | 3.116439547 | 7.53E-04 |
| 101249363 | LOC101249363 | 1.02987936 | 0.005575248 |
| 101249387 | LOC101249387 | 3.725595489 | 2.86E-06 |
| 101249399 | LOC101249399 | 1.436749623 | 0.00153345 |
| 101249400 | LOC101249400 | 6.454365461 | 1.66E-08 |
| 101249433 | LOC101249433 | 1.371523426 | 0.003272207 |
| 101249445 | LOC101249445 | 1.867769363 | 7.98E-04 |
| 101249481 | LOC101249481 | 3.969570678 | 9.12E-06 |
| 101249483 | TRM31 | 1.840482621 | 0.006998275 |
| 101249527 | LOC101249527 | 1.936233376 | 0.002308398 |
| 101249528 | LOC101249528 | 3.773926942 | 0.004461778 |
| 101249534 | LOC101249534 | 1.53460813 | 3.40E-08 |
| 101249553 | LOC101249553 | 1.321820132 | 0.007005989 |
| 101249561 | LOC101249561 | 3.019681885 | 3.00E-05 |
| 101249569 | LOC101249569 | 3.016925409 | 6.74E-06 |
| 101249575 | LOC101249575 | 5.901773718 | 6.95E-05 |
| 101249595 | LOC101249595 | 2.834076128 | 1.47E-04 |
| 101249601 | LOC101249601 | 1.333707512 | 8.82E-04 |
| 101249625 | LOC101249625 | 4.156723186 | 1.17E-10 |
| 101249637 | LOC101249637 | 4.151913557 | 0.007309626 |
| 101249638 | LOC101249638 | 1.119106749 | 2.32E-04 |
| 101249643 | LOC101249643 | 7.973558788 | 1.87E-17 |
| 101249664 | LOC101249664 | 1.873217486 | 0.007333229 |
| 101249671 | LOC101249671 | 1.418889044 | 0.003799744 |
| 101249675 | LOC101249675 | 1.351882256 | 0.008348391 |
| 101249679 | LOC101249679 | 3.119591177 | 2.44E-07 |
| 101249700 | LOC101249700 | 1.597465358 | 4.38E-05 |
| 101249721 | LOC101249721 | 4.605522666 | 1.46E-11 |
| 101249726 | LOC101249726 | 6.220655052 | 6.88E-08 |
| 101249733 | LOC101249733 | 2.410428 | 0.008045041 |
| 101249743 | LOC101249743 | 3.127425553 | 5.78E-05 |
| 101249753 | LOC101249753 | 1.032385969 | 0.006724247 |
| 101249768 | LOC101249768 | 2.508647948 | 0.004785929 |
| 101249777 | LOC101249777 | 1.626827358 | 0.001063819 |
| 101249779 | LOC101249779 | 1.557093489 | 4.68E-04 |
| 101249787 | FBA2 | 2.536271936 | 1.51E-04 |
| 101249853 | LOC101249853 | 1.118654765 | 0.007930768 |
| 101249876 | LOC101249876 | 2.26571002 | 2.94E-05 |
| 101249935 | LOC101249935 | 1.629312332 | 9.96E-06 |
| 101249950 | LOC101249950 | 2.113651656 | 0.002604271 |
| 101249954 | LOC101249954 | 6.690872769 | 1.06E-06 |
| 101249959 | FKBP42 | 1.147533901 | 0.009068224 |
| 101249979 | LOC101249979 | 1.285570808 | 0.00913422 |
| 101249991 | LOC101249991 | 2.78139756 | 4.89E-09 |
| 101250003 | LOC101250003 | 1.279113977 | 4.73E-04 |
| 101250012 | SQO1 | 1.953186278 | 6.24E-06 |
| 101250037 | LOC101250037 | 1.942390137 | 0.002972002 |
| 101250050 | LOC101250050 | 1.344170624 | 0.003642614 |
| 101250055 | LOC101250055 | 4.058723011 | 2.13E-04 |
| 101250067 | LOC101250067 | 2.92415257 | 0.007508132 |
| 101250069 | LOC101250069 | 3.920790182 | 4.25E-04 |
| 101250094 | LOC101250094 | 1.333413438 | 0.003360784 |
| 101250106 | LOC101250106 | 1.951448233 | 0.002470479 |
| 101250128 | LOC101250128 | 3.017262566 | 2.12E-12 |
| 101250133 | LOC101250133 | 2.774609363 | 2.98E-07 |
| 101250147 | LOC101250147 | 1.321808072 | 0.006450355 |
| 101250162 | LOC101250162 | 2.833482116 | 0.001323781 |
| 101250165 | LOC101250165 | 1.211444777 | 7.18E-04 |
| 101250191 | LOC101250191 | 5.646757371 | 1.00E-07 |
| 101250199 | SAP10 | 1.934531203 | 8.94E-04 |
| 101250202 | LOC101250202 | 4.842851687 | 1.84E-06 |
| 101250213 | LOC101250213 | 3.425262575 | 2.76E-21 |
| 101250254 | LOC101250254 | 1.961702502 | 6.82E-05 |
| 101250259 | LOC101250259 | 2.556468336 | 5.20E-05 |
| 101250282 | LOC101250282 | 2.300558882 | 0.001619775 |
| 101250287 | LOC101250287 | 1.286840992 | 2.96E-04 |
| 101250289 | LOC101250289 | 2.315800113 | 0.003772423 |
| 101250295 | LOC101250295 | 2.335998675 | 1.37E-05 |
| 101250322 | ABCC5 | 1.797849888 | 2.27E-04 |
| 101250324 | LOC101250324 | 2.088526376 | 1.72E-04 |
| 101250353 | LOC101250353 | 1.425937036 | 0.003634615 |
| 101250354 | LOC101250354 | 1.08822289 | 7.27E-04 |
| 101250355 | LOC101250355 | 3.063049103 | 0.002422658 |
| 101250358 | LOC101250358 | 1.229495714 | 3.82E-05 |
| 101250380 | LOC101250380 | 3.478689106 | 3.10E-04 |
| 101250395 | LOC101250395 | 2.06327527 | 0.001039752 |
| 101250396 | LOC101250396 | 1.517925942 | 7.55E-04 |
| 101250401 | LOC101250401 | 3.805227143 | 1.99E-13 |
| 101250408 | LOC101250408 | 2.860836566 | 2.84E-05 |
| 101250433 | LOC101250433 | 1.229128218 | 0.004081382 |
| 101250450 | UGT75C1 | 3.842443926 | 3.87E-06 |
| 101250467 | LOC101250467 | 5.703800446 | 7.61E-05 |
| 101250472 | LOC101250472 | 2.101540647 | 2.21E-10 |
| 101250479 | LOC101250479 | 5.100349429 | 2.02E-14 |
| 101250489 | LOC101250489 | 1.357090788 | 1.08E-04 |
| 101250495 | LOC101250495 | 2.829438009 | 1.82E-05 |
| 101250512 | LOC101250512 | 2.310484034 | 1.26E-07 |
| 101250516 | LOC101250516 | 4.406463431 | 3.31E-05 |
| 101250521 | LOC101250521 | 8.300880832 | 7.04E-08 |
| 101250528 | LOC101250528 | 1.78662164 | 9.31E-06 |
| 101250534 | LOC101250534 | 3.499649173 | 0.001007046 |
| 101250536 | LOC101250536 | 1.678843989 | 2.91E-07 |
| 101250559 | LOC101250559 | 4.797233731 | 3.91E-08 |
| 101250560 | LOC101250560 | 1.473999761 | 0.005942547 |
| 101250562 | LOC101250562 | 2.852915498 | 3.51E-06 |
| 101250572 | LOC101250572 | 2.568373094 | 0.006742662 |
| 101250586 | LOC101250586 | 3.049454667 | 6.82E-05 |
| 101250588 | LOC101250588 | 2.903295091 | 2.68E-06 |
| 101250601 | LOC101250601 | 1.629623315 | 5.39E-04 |
| 101250624 | LOC101250624 | 2.100860013 | 2.43E-04 |
| 101250625 | LOC101250625 | 1.949465579 | 5.24E-07 |
| 101250635 | LOC101250635 | 2.476380771 | 7.33E-20 |
| 101250672 | LOC101250672 | 1.916680165 | 4.94E-04 |
| 101250692 | LOC101250692 | 3.684622984 | 0.001324867 |
| 101250708 | LOC101250708 | 5.857450007 | 3.13E-10 |
| 101250725 | LOC101250725 | 5.386929906 | 7.08E-24 |
| 101250739 | LOC101250739 | 1.239478848 | 2.25E-05 |
| 101250740 | LOC101250740 | 3.308147233 | 1.10E-04 |
| 101250745 | LOC101250745 | 2.568610946 | 6.73E-07 |
| 101250756 | LOC101250756 | 2.776010051 | 0.001456609 |
| 101250768 | LOC101250768 | 3.344628638 | 0.002126051 |
| 101250847 | LOC101250847 | 6.603001395 | 2.18E-07 |
| 101250851 | LOC101250851 | 1.856861525 | 0.001059468 |
| 101250853 | FBA3 | 2.464188003 | 6.37E-06 |
| 101250873 | LOC101250873 | 2.140176818 | 0.007453134 |
| 101250881 | LOC101250881 | 2.351599968 | 0.00363231 |
| 101250920 | LOC101250920 | 3.924006766 | 5.40E-08 |
| 101250924 | LOC101250924 | 8.064022984 | 3.59E-13 |
| 101250934 | LOC101250934 | 3.223291156 | 8.59E-09 |
| 101250945 | LOC101250945 | 1.98843914 | 0.001610427 |
| 101250955 | LOC101250955 | 1.957439617 | 0.004434174 |
| 101250974 | AKR4B | 3.217708399 | 0.004306261 |
| 101251005 | LOC101251005 | 4.929430907 | 9.59E-08 |
| 101251036 | LOC101251036 | 5.606957965 | 5.44E-08 |
| 101251042 | LOC101251042 | 3.176596527 | 1.58E-12 |
| 101251084 | ERF84 | 4.125972253 | 2.76E-06 |
| 101251116 | LOC101251116 | 2.914065112 | 4.08E-09 |
| 101251145 | Rcr3 | 4.054720779 | 4.63E-08 |
| 101251153 | LOC101251153 | 2.264953277 | 0.00106868 |
| 101251154 | LOC101251154 | 8.424166706 | 5.90E-05 |
| 101251162 | LOC101251162 | 6.278218816 | 6.08E-04 |
| 101251168 | LOC101251168 | 1.886910164 | 3.49E-05 |
| 101251175 | LOC101251175 | 2.720733423 | 0.003349495 |
| 101251213 | LOC101251213 | 2.448545271 | 1.85E-09 |
| 101251219 | LOC101251219 | 1.130599083 | 0.003762283 |
| 101251228 | LOC101251228 | 1.66098594 | 1.84E-07 |
| 101251239 | LOC101251239 | 1.74511672 | 6.71E-05 |
| 101251258 | LOC101251258 | 2.826565177 | 2.99E-04 |
| 101251259 | LOC101251259 | 6.353928593 | 3.18E-15 |
| 101251278 | LOC101251278 | 2.296340211 | 0.006727275 |
| 101251299 | LOC101251299 | 1.394828942 | 1.90E-04 |
| 101251327 | LOC101251327 | 2.92892202 | 5.97E-05 |
| 101251343 | LOC101251343 | 1.103156705 | 9.23E-04 |
| 101251368 | LOC101251368 | 2.010935615 | 0.008822646 |
| 101251402 | LOC101251402 | 2.796731458 | 2.99E-08 |
| 101251407 | LOC101251407 | 2.727334921 | 1.75E-04 |
| 101251418 | LOC101251418 | 1.542192086 | 5.17E-06 |
| 101251419 | LOC101251419 | 1.039229885 | 0.002189601 |
| 101251423 | LOC101251423 | 2.015599412 | 6.53E-04 |
| 101251439 | LOC101251439 | 3.219676554 | 0.00353412 |
| 101251444 | GLR1.2 | 4.314515949 | 0.003213534 |
| 101251449 | LOC101251449 | 6.814501738 | 7.32E-05 |
| 101251472 | LOC101251472 | 1.38876803 | 0.005934659 |
| 101251473 | LOC101251473 | 1.926473859 | 2.39E-04 |
| 101251482 | LOC101251482 | 7.308394288 | 3.31E-06 |
| 101251501 | GPAT6 | 1.631848185 | 0.005831639 |
| 101251503 | LOC101251503 | 5.82149725 | 1.94E-06 |
| 101251509 | LOC101251509 | 1.349006548 | 0.001163874 |
| 101251512 | LOC101251512 | 1.054932984 | 2.39E-05 |
| 101251538 | LOC101251538 | 3.117939222 | 8.40E-07 |
| 101251560 | LOC101251560 | 4.793481545 | 9.88E-06 |
| 101251576 | LOC101251576 | 7.953920193 | 9.99E-08 |
| 101251580 | LOC101251580 | 3.251322072 | 4.49E-05 |
| 101251600 | LOC101251600 | 2.240473767 | 0.006810868 |
| 101251620 | LOC101251620 | 1.269353331 | 0.005696486 |
| 101251622 | PT3 | 2.824601002 | 5.71E-04 |
| 101251623 | LOC101251623 | 6.34479092 | 3.81E-05 |
| 101251627 | LOC101251627 | 2.074435254 | 0.003823018 |
| 101251640 | LOC101251640 | 2.548361349 | 2.82E-05 |
| 101251641 | LOC101251641 | 1.348520982 | 4.67E-04 |
| 101251649 | LOC101251649 | 4.376282235 | 0.004298303 |
| 101251660 | LOC101251660 | 1.250788759 | 7.70E-04 |
| 101251666 | LOC101251666 | 1.453778437 | 4.34E-06 |
| 101251673 | LOC101251673 | 5.325769582 | 4.54E-04 |
| 101251682 | GH3-5 | 1.654164878 | 1.17E-05 |
| 101251693 | LOC101251693 | 4.260224599 | 0.004512237 |
| 101251695 | HKT1;2 | 2.145977853 | 5.75E-04 |
| 101251739 | LOC101251739 | 1.642029217 | 6.09E-05 |
| 101251740 | LOC101251740 | 6.791220075 | 0.001467555 |
| 101251778 | WRKY75 | 3.980445409 | 2.20E-04 |
| 101251789 | LOC101251789 | 1.509918449 | 0.002171824 |
| 101251812 | LOC101251812 | 3.173235236 | 1.90E-04 |
| 101251822 | LOC101251822 | 1.364640001 | 1.73E-04 |
| 101251856 | LOC101251856 | 4.020859035 | 1.51E-10 |
| 101251873 | LOC101251873 | 1.257892966 | 0.005086726 |
| 101251874 | LOC101251874 | 1.341269012 | 0.008377973 |
| 101251889 | LOC101251889 | 5.285560853 | 4.48E-07 |
| 101251891 | LOC101251891 | 2.402971124 | 2.10E-06 |
| 101251898 | LOC101251898 | 3.145715229 | 2.40E-08 |
| 101251905 | LOC101251905 | 2.461137747 | 1.58E-12 |
| 101251913 | LOC101251913 | 3.576245725 | 4.22E-10 |
| 101251943 | LOC101251943 | 2.164836966 | 1.12E-04 |
| 101251949 | LOC101251949 | 2.903726371 | 1.86E-06 |
| 101251956 | LOC101251956 | 1.083639137 | 0.001807778 |
| 101251972 | LOC101251972 | 3.413349413 | 3.90E-19 |
| 101251973 | LOC101251973 | 1.79699438 | 4.17E-04 |
| 101251991 | LOC101251991 | 6.882799865 | 2.63E-04 |
| 101252004 | LOC101252004 | 1.886248252 | 0.006394535 |
| 101252005 | LOC101252005 | 1.514307471 | 0.002345207 |
| 101252054 | LOC101252054 | 1.668072917 | 8.23E-04 |
| 101252078 | LOC101252078 | 1.05582154 | 0.006819819 |
| 101252097 | LOC101252097 | 2.121373508 | 5.29E-05 |
| 101252103 | LOC101252103 | 4.786227148 | 1.15E-14 |
| 101252121 | LOC101252121 | 1.84144968 | 1.11E-04 |
| 101252122 | LOC101252122 | 1.071952328 | 0.003408146 |
| 101252149 | LOC101252149 | 1.940979369 | 2.79E-07 |
| 101252162 | LOC101252162 | 1.468228251 | 6.38E-04 |
| 101252201 | LOC101252201 | 3.837125905 | 0.001308761 |
| 101252202 | LOC101252202 | 6.862157406 | 1.03E-15 |
| 101252243 | LOC101252243 | 3.471242096 | 6.98E-05 |
| 101252253 | SIZ1 | 1.006542522 | 0.00608407 |
| 101252258 | LOC101252258 | 1.017836715 | 1.16E-04 |
| 101252269 | LOC101252269 | 2.577679629 | 0.008545655 |
| 101252287 | LOC101252287 | 1.252998153 | 1.65E-04 |
| 101252323 | LOC101252323 | 1.11168067 | 0.004774829 |
| 101252342 | LOC101252342 | 3.083882444 | 1.68E-05 |
| 101252390 | LOC101252390 | 2.06288968 | 4.24E-05 |
| 101252411 | LOC101252411 | 1.33429377 | 1.91E-05 |
| 101252418 | LOC101252418 | 3.869808839 | 7.04E-06 |
| 101252420 | LOC101252420 | 1.927935796 | 6.57E-06 |
| 101252433 | LOC101252433 | 1.598740478 | 0.002480802 |
| 101252452 | LOC101252452 | 3.072747384 | 8.80E-11 |
| 101252453 | LOC101252453 | 6.904910563 | 2.72E-04 |
| 101252465 | LOC101252465 | 4.273969376 | 7.78E-06 |
| 101252467 | LOC101252467 | 5.58368619 | 5.34E-04 |
| 101252472 | LOC101252472 | 1.191358852 | 1.73E-04 |
| 101252479 | LOC101252479 | 2.71647311 | 0.003329492 |
| 101252483 | LOC101252483 | 2.805271862 | 3.32E-06 |
| 101252490 | LOC101252490 | 2.341707257 | 7.75E-05 |
| 101252550 | LOC101252550 | 3.956119955 | 4.20E-12 |
| 101252555 | LOC101252555 | 2.310125888 | 3.92E-05 |
| 101252559 | LOC101252559 | 1.081321336 | 0.004898527 |
| 101252563 | LOC101252563 | 1.468796889 | 6.26E-05 |
| 101252571 | LOC101252571 | 4.463681771 | 1.06E-05 |
| 101252576 | LOC101252576 | 1.182317659 | 8.69E-04 |
| 101252601 | LOC101252601 | 1.792415916 | 7.47E-04 |
| 101252617 | LOC101252617 | 1.25146768 | 0.007228654 |
| 101252618 | LOC101252618 | 1.608786008 | 1.60E-05 |
| 101252623 | LOC101252623 | 1.659306356 | 6.23E-08 |
| 101252626 | LOC101252626 | 2.840237809 | 0.001113614 |
| 101252652 | LOC101252652 | 1.431217751 | 0.0020922 |
| 101252660 | LOC101252660 | 3.536995166 | 1.68E-04 |
| 101252682 | LOC101252682 | 4.718031977 | 2.36E-06 |
| 101252683 | LOC101252683 | 1.451260815 | 0.002752916 |
| 101252695 | LOC101252695 | 1.543446544 | 1.04E-08 |
| 101252700 | LOC101252700 | 4.417152532 | 4.62E-04 |
| 101252701 | LOC101252701 | 3.666560943 | 2.78E-10 |
| 101252748 | LOC101252748 | 2.096052417 | 7.48E-05 |
| 101252749 | LOC101252749 | 2.555989485 | 2.65E-05 |
| 101252762 | LOC101252762 | 1.4720239 | 1.12E-04 |
| 101252767 | LOC101252767 | 2.047199009 | 8.93E-05 |
| 101252813 | LOC101252813 | 1.660733312 | 0.001754799 |
| 101252832 | LOC101252832 | 1.994247096 | 0.001878588 |
| 101252834 | LOC101252834 | 3.330800028 | 2.04E-14 |
| 101252838 | LOC101252838 | 2.289107565 | 0.001801833 |
| 101252842 | LOC101252842 | 4.361780375 | 0.00300141 |
| 101252844 | LOC101252844 | 1.054222333 | 0.006451195 |
| 101252846 | LOC101252846 | 2.032221386 | 7.06E-08 |
| 101252852 | LOC101252852 | 2.84728898 | 1.76E-09 |
| 101252861 | LOC101252861 | 3.87482622 | 7.80E-04 |
| 101252869 | LOC101252869 | 1.396999511 | 0.001369353 |
| 101252874 | LOC101252874 | 2.223180997 | 5.12E-05 |
| 101252913 | LOC101252913 | 3.122041684 | 1.09E-05 |
| 101252966 | LOC101252966 | 2.344135956 | 4.35E-06 |
| 101252968 | LOC101252968 | 1.847127216 | 2.31E-04 |
| 101252980 | LOC101252980 | 1.256757446 | 0.009103093 |
| 101253007 | SppS | 3.017003266 | 1.14E-11 |
| 101253030 | PMT5 | 1.764474271 | 8.82E-06 |
| 101253040 | LOC101253040 | 1.978041658 | 0.00158296 |
| 101253053 | LOC101253053 | 1.937998085 | 6.22E-04 |
| 101253098 | LOC101253098 | 1.536258463 | 1.79E-09 |
| 101253102 | LOC101253102 | 1.568898821 | 0.002038544 |
| 101253151 | LOC101253151 | 1.751248394 | 0.004577324 |
| 101253157 | LOC101253157 | 3.37368289 | 2.57E-05 |
| 101253200 | FKBP62a | 1.3853346 | 0.002820076 |
| 101253205 | LOC101253205 | 1.829222062 | 8.11E-05 |
| 101253219 | LOC101253219 | 1.732849142 | 1.31E-08 |
| 101253223 | LOC101253223 | 1.166888981 | 0.00525503 |
| 101253225 | LOC101253225 | 2.076808276 | 1.92E-06 |
| 101253234 | LOC101253234 | 2.231678737 | 0.00387351 |
| 101253239 | LOC101253239 | 2.847140177 | 7.93E-14 |
| 101253242 | LOC101253242 | 3.803453145 | 2.13E-11 |
| 101253254 | LOC101253254 | 3.239383197 | 3.76E-05 |
| 101253266 | LOC101253266 | 1.730430867 | 0.001021704 |
| 101253267 | LOC101253267 | 2.580401063 | 8.69E-05 |
| 101253320 | LOC101253320 | 5.708763325 | 1.86E-09 |
| 101253341 | LOC101253341 | 2.780193743 | 3.72E-07 |
| 101253414 | LOC101253414 | 1.556690836 | 9.03E-06 |
| 101253417 | LOC101253417 | 1.677718878 | 1.17E-04 |
| 101253418 | LOC101253418 | 1.73210079 | 2.16E-04 |
| 101253445 | LOC101253445 | 3.003322217 | 1.97E-04 |
| 101253454 | LOC101253454 | 1.673793278 | 0.006784022 |
| 101253482 | LOC101253482 | 2.27167002 | 1.82E-09 |
| 101253498 | LOC101253498 | 1.713626034 | 2.94E-05 |
| 101253503 | LOC101253503 | 4.530272513 | 1.22E-11 |
| 101253545 | LOC101253545 | 6.824548435 | 1.78E-14 |
| 101253559 | LOC101253559 | 1.089382751 | 2.22E-04 |
| 101253579 | LOC101253579 | 2.738831315 | 9.47E-05 |
| 101253592 | LOC101253592 | 4.973080618 | 1.57E-07 |
| 101253603 | LOC101253603 | 5.191523353 | 0.00323119 |
| 101253631 | LOC101253631 | 1.364054023 | 2.98E-04 |
| 101253647 | LOC101253647 | 1.038351075 | 0.007986436 |
| 101253650 | LOC101253650 | 1.209218497 | 2.63E-04 |
| 101253663 | LOC101253663 | 2.878063661 | 2.11E-06 |
| 101253674 | LOC101253674 | 5.854631085 | 2.95E-17 |
| 101253692 | LOC101253692 | 1.667321668 | 8.37E-05 |
| 101253697 | LOC101253697 | 1.116703618 | 0.003911831 |
| 101253719 | LOC101253719 | 1.519026364 | 0.001126907 |
| 101253754 | LOC101253754 | 1.341937007 | 0.007973899 |
| 101253782 | LOC101253782 | 1.462555821 | 9.09E-05 |
| 101253788 | LOC101253788 | 2.125424096 | 1.21E-04 |
| 101253789 | LOC101253789 | 1.255020245 | 0.007642109 |
| 101253806 | LOC101253806 | 1.607873222 | 4.32E-04 |
| 101253830 | LOC101253830 | 1.601161252 | 1.78E-04 |
| 101253845 | LOC101253845 | 2.173188724 | 2.37E-10 |
| 101253855 | LOC101253855 | 1.467271503 | 0.005894494 |
| 101253894 | LOC101253894 | 3.36345844 | 0.004423973 |
| 101253897 | LOC101253897 | 2.412739282 | 0.001956979 |
| 101253907 | LOC101253907 | 1.410821615 | 5.36E-05 |
| 101253958 | LOC101253958 | 1.941008545 | 7.92E-05 |
| 101253972 | LOC101253972 | 8.188463037 | 7.42E-37 |
| 101253973 | LOC101253973 | 1.690212613 | 5.64E-04 |
| 101253978 | LOC101253978 | 1.276320319 | 0.005706229 |
| 101253979 | LOC101253979 | 6.250853836 | 0.005524121 |
| 101254056 | LOC101254056 | 1.047954039 | 0.007763352 |
| 101254067 | LOC101254067 | 1.103597741 | 0.004091125 |
| 101254071 | LOC101254071 | 2.07982494 | 7.75E-06 |
| 101254072 | LOC101254072 | 3.051927892 | 0.001997699 |
| 101254080 | LOC101254080 | 1.690976358 | 3.32E-04 |
| 101254084 | LOC101254084 | 3.433607517 | 2.21E-09 |
| 101254089 | LOC101254089 | 1.734550343 | 0.006489942 |
| 101254096 | LOC101254096 | 1.301532716 | 0.004862721 |
| 101254112 | LOC101254112 | 2.240272974 | 1.59E-04 |
| 101254113 | LOC101254113 | 3.631247609 | 5.23E-08 |
| 101254118 | LOC101254118 | 1.442160743 | 0.005360386 |
| 101254119 | LOC101254119 | 1.684497447 | 1.48E-04 |
| 101254147 | LOC101254147 | 1.261604346 | 1.87E-04 |
| 101254155 | LOC101254155 | 4.567257676 | 5.33E-06 |
| 101254173 | LOC101254173 | 4.05164374 | 4.15E-04 |
| 101254175 | LOC101254175 | 1.411312844 | 1.57E-05 |
| 101254178 | LOC101254178 | 3.045105497 | 2.91E-09 |
| 101254182 | LOC101254182 | 1.52717697 | 3.80E-07 |
| 101254186 | LOC101254186 | 1.393461616 | 7.82E-04 |
| 101254211 | LOC101254211 | 1.693158527 | 4.23E-05 |
| 101254223 | LOC101254223 | 2.203676234 | 4.24E-05 |
| 101254229 | LOC101254229 | 3.901694039 | 5.20E-04 |
| 101254238 | LOC101254238 | 2.051771303 | 3.33E-04 |
| 101254240 | LOC101254240 | 1.64220977 | 1.11E-05 |
| 101254364 | LOC101254364 | 2.582952124 | 8.59E-25 |
| 101254370 | LOC101254370 | 2.812110907 | 1.78E-07 |
| 101254380 | LOC101254380 | 2.43488726 | 5.69E-06 |
| 101254407 | LOC101254407 | 1.654257483 | 0.004860093 |
| 101254424 | LOC101254424 | 1.195041841 | 0.003147724 |
| 101254428 | LOC101254428 | 6.171301159 | 4.62E-05 |
| 101254442 | LOC101254442 | 1.031914133 | 0.007823086 |
| 101254498 | LOC101254498 | 1.692332584 | 9.08E-04 |
| 101254502 | LOC101254502 | 1.700762015 | 0.005656265 |
| 101254517 | LOC101254517 | 1.738448251 | 0.00242859 |
| 101254550 | LOC101254550 | 2.645981355 | 1.44E-06 |
| 101254577 | LOC101254577 | 1.428506948 | 0.004450547 |
| 101254582 | LOC101254582 | 1.816589177 | 0.008113386 |
| 101254583 | LOC101254583 | 5.012413434 | 2.35E-06 |
| 101254600 | LOC101254600 | 2.901709184 | 0.006183988 |
| 101254608 | LOC101254608 | 3.03361299 | 7.04E-06 |
| 101254632 | LOC101254632 | 4.200897698 | 0.005327198 |
| 101254650 | LOC101254650 | 6.466013888 | 2.24E-26 |
| 101254665 | LOC101254665 | 1.326649441 | 2.57E-06 |
| 101254666 | LOC101254666 | 1.289064563 | 2.58E-04 |
| 101254668 | CYP736A4 | 2.615752121 | 3.37E-05 |
| 101254669 | LOC101254669 | 6.001662084 | 8.84E-14 |
| 101254707 | LOC101254707 | 4.041957503 | 0.003732272 |
| 101254711 | LOC101254711 | 1.527682346 | 9.95E-04 |
| 101254722 | LOC101254722 | 1.669462694 | 4.44E-04 |
| 101254738 | LOC101254738 | 1.329432259 | 0.008105474 |
| 101254776 | LOC101254776 | 1.989616429 | 2.01E-05 |
| 101254798 | ABCG13 | 4.986173222 | 1.51E-06 |
| 101254822 | LOC101254822 | 1.016725745 | 9.77E-04 |
| 101254832 | LOC101254832 | 1.939996267 | 0.00902869 |
| 101254845 | LOC101254845 | 1.176307482 | 0.004102894 |
| 101254846 | LOC101254846 | 1.386448499 | 0.001375064 |
| 101254847 | LOC101254847 | 6.490416549 | 6.96E-09 |
| 101254883 | LOC101254883 | 1.16673863 | 3.22E-04 |
| 101254887 | LOC101254887 | 1.711013734 | 6.69E-05 |
| 101254893 | LOC101254893 | 1.731127161 | 9.03E-05 |
| 101254895 | LOC101254895 | 1.566171627 | 9.14E-04 |
| 101254908 | LOC101254908 | 7.151164436 | 0.002244518 |
| 101254912 | LOC101254912 | 2.88006821 | 1.95E-05 |
| 101254927 | LOC101254927 | 2.221898827 | 0.001129226 |
| 101254946 | LOC101254946 | 6.287445859 | 1.96E-13 |
| 101254949 | LOC101254949 | 1.085418207 | 0.008985058 |
| 101254958 | LOC101254958 | 2.379881535 | 8.29E-06 |
| 101254974 | LOC101254974 | 1.883612507 | 4.62E-04 |
| 101255006 | LOC101255006 | 2.883236606 | 8.83E-08 |
| 101255020 | LOC101255020 | 1.658449212 | 9.70E-07 |
| 101255033 | LOC101255033 | 1.950907887 | 1.12E-05 |
| 101255038 | LOC101255038 | 2.893599019 | 2.81E-04 |
| 101255051 | LOC101255051 | 5.99124623 | 1.97E-18 |
| 101255065 | LOC101255065 | 1.089119033 | 0.004036471 |
| 101255086 | LOC101255086 | 1.111796749 | 0.00319256 |
| 101255090 | LOC101255090 | 2.013146158 | 2.99E-07 |
| 101255099 | LOC101255099 | 1.558481792 | 5.50E-04 |
| 101255100 | LOC101255100 | 2.331954366 | 0.006205971 |
| 101255117 | LOC101255117 | 1.468915966 | 6.89E-05 |
| 101255136 | LOC101255136 | 2.325195136 | 3.79E-09 |
| 101255139 | LOC101255139 | 1.457011766 | 0.001640654 |
| 101255145 | LOC101255145 | 2.383211345 | 3.65E-07 |
| 101255164 | LOC101255164 | 5.857538864 | 1.52E-08 |
| 101255178 | LOC101255178 | 2.608111018 | 0.006777569 |
| 101255215 | LOC101255215 | 1.166853894 | 0.001327339 |
| 101255217 | LOC101255217 | 8.465505655 | 4.99E-16 |
| 101255218 | LOC101255218 | 4.874220788 | 2.69E-06 |
| 101255243 | LOC101255243 | 2.909428624 | 0.004723047 |
| 101255261 | LOC101255261 | 1.806653882 | 3.35E-04 |
| 101255267 | LOC101255267 | 3.121821205 | 0.007891942 |
| 101255298 | LOC101255298 | 1.604636664 | 3.65E-04 |
| 101255300 | LOC101255300 | 2.466870783 | 5.18E-05 |
| 101255301 | LOC101255301 | 3.139817347 | 7.53E-05 |
| 101255303 | IAA4 | 3.222155437 | 1.28E-10 |
| 101255305 | LOC101255305 | 2.63917492 | 3.38E-06 |
| 101255316 | LOC101255316 | 5.266065022 | 1.27E-08 |
| 101255328 | LOC101255328 | 1.957339332 | 1.48E-09 |
| 101255341 | LOC101255341 | 5.215110997 | 0.00199943 |
| 101255345 | LOC101255345 | 1.52821661 | 0.002839729 |
| 101255353 | LOC101255353 | 4.258609116 | 3.21E-16 |
| 101255399 | LOC101255399 | 1.135961727 | 1.34E-04 |
| 101255401 | LOC101255401 | 1.973912733 | 2.09E-06 |
| 101255410 | Dof22 | 1.348562382 | 0.007216562 |
| 101255411 | LOC101255411 | 1.851233574 | 2.66E-04 |
| 101255416 | LOC101255416 | 4.991188866 | 1.15E-07 |
| 101255449 | LOC101255449 | 1.449667288 | 0.003668121 |
| 101255454 | LOC101255454 | 1.16311851 | 0.002268949 |
| 101255458 | LOC101255458 | 1.21445479 | 0.007711622 |
| 101255468 | Lemir | 7.832144883 | 3.11E-15 |
| 101255470 | LOC101255470 | 3.734538704 | 0.001123917 |
| 101255474 | LOC101255474 | 5.25665459 | 1.01E-07 |
| 101255491 | LOC101255491 | 8.209295964 | 5.28E-07 |
| 101255492 | LOC101255492 | 6.20844197 | 8.50E-09 |
| 101255495 | LOC101255495 | 3.281653362 | 5.64E-10 |
| 101255519 | LOC101255519 | 5.577745579 | 1.91E-05 |
| 101255525 | LOC101255525 | 3.505601523 | 0.001102784 |
| 101255550 | LOC101255550 | 1.303669639 | 0.001520123 |
| 101255551 | LOC101255551 | 2.248474343 | 3.24E-06 |
| 101255565 | LOC101255565 | 1.360381902 | 0.001144127 |
| 101255572 | LOC101255572 | 2.318282782 | 4.65E-04 |
| 101255579 | LOC101255579 | 1.15521163 | 0.001158921 |
| 101255583 | PTC52 | 2.903886096 | 5.79E-10 |
| 101255592 | LOC101255592 | 2.806108221 | 0.003253584 |
| 101255600 | LOC101255600 | 4.800607817 | 1.07E-07 |
| 101255604 | LOC101255604 | 1.709237203 | 9.82E-05 |
| 101255608 | LOC101255608 | 2.793623172 | 0.002194254 |
| 101255648 | LOC101255648 | 6.020509542 | 2.18E-06 |
| 101255657 | LOC101255657 | 1.938238286 | 3.63E-06 |
| 101255692 | LOC101255692 | 1.255056829 | 0.001029827 |
| 101255695 | LOC101255695 | 1.349767632 | 0.001390584 |
| 101255696 | LOC101255696 | 1.201973969 | 0.001121144 |
| 101255705 | LOC101255705 | 3.767511626 | 2.31E-04 |
| 101255717 | LOC101255717 | 4.964674883 | 3.64E-26 |
| 101255726 | LOC101255726 | 2.54032441 | 8.12E-06 |
| 101255738 | LOC101255738 | 1.455244711 | 8.45E-06 |
| 101255786 | LOC101255786 | 1.777288635 | 5.94E-04 |
| 101255805 | LOC101255805 | 1.094643864 | 7.71E-04 |
| 101255811 | LOC101255811 | 2.903193759 | 9.30E-04 |
| 101255823 | LOC101255823 | 2.442652801 | 6.24E-06 |
| 101255835 | LOC101255835 | 1.401657126 | 0.003842596 |
| 101255851 | LOC101255851 | 5.214949337 | 0.004820866 |
| 101255854 | LOC101255854 | 8.526665577 | 0.003663374 |
| 101255868 | LOC101255868 | 1.736755655 | 1.57E-06 |
| 101255884 | LOC101255884 | 1.118004939 | 8.18E-04 |
| 101255885 | LOC101255885 | 5.359943683 | 1.65E-11 |
| 101255902 | LOC101255902 | 1.051639146 | 0.002051618 |
| 101255924 | ACAT5 | 1.105113361 | 0.004064459 |
| 101255928 | LOC101255928 | 1.68648444 | 0.004480616 |
| 101255946 | LOC101255946 | 1.615546923 | 6.11E-04 |
| 101255951 | LOC101255951 | 1.334772813 | 0.002911359 |
| 101255971 | LOC101255971 | 2.667509357 | 8.55E-04 |
| 101255972 | LOC101255972 | 5.346804461 | 1.19E-11 |
| 101255977 | LOC101255977 | 3.440597341 | 0.0054013 |
| 101255979 | LOC101255979 | 1.718620405 | 0.003755882 |
| 101256001 | LOC101256001 | 1.010066265 | 0.008057231 |
| 101256018 | LOC101256018 | 1.083328192 | 0.005570357 |
| 101256019 | LOC101256019 | 1.710794288 | 5.37E-04 |
| 101256020 | LOC101256020 | 2.124064285 | 2.45E-06 |
| 101256074 | LOC101256074 | 1.238598164 | 9.54E-04 |
| 101256077 | ABCG35 | 1.534596916 | 2.51E-04 |
| 101256086 | LOC101256086 | 3.905906521 | 7.78E-04 |
| 101256097 | LOC101256097 | 1.906561304 | 1.56E-04 |
| 101256105 | LOC101256105 | 6.129347475 | 6.78E-16 |
| 101256107 | LOC101256107 | 5.750204029 | 2.65E-36 |
| 101256116 | LOC101256116 | 3.99875731 | 1.39E-06 |
| 101256127 | LOC101256127 | 2.197924389 | 0.001340529 |
| 101256151 | LOC101256151 | 3.115346356 | 0.001625714 |
| 101256167 | LOC101256167 | 2.243258151 | 0.001326852 |
| 101256169 | LOC101256169 | 1.365254646 | 0.002762869 |
| 101256176 | LOC101256176 | 4.316594418 | 1.67E-08 |
| 101256189 | LOC101256189 | 3.077365504 | 2.04E-16 |
| 101256205 | LOC101256205 | 1.821134622 | 0.001241713 |
| 101256232 | LOC101256232 | 2.461104067 | 0.001142891 |
| 101256243 | LOC101256243 | 1.531189959 | 0.007193363 |
| 101256248 | LOC101256248 | 2.132621492 | 0.002480411 |
| 101256262 | LOC101256262 | 1.84558318 | 0.008442716 |
| 101256268 | LOC101256268 | 1.838702703 | 1.83E-05 |
| 101256272 | LOC101256272 | 5.81333611 | 0.002453143 |
| 101256278 | LOC101256278 | 1.86020979 | 0.001740914 |
| 101256309 | LOC101256309 | 1.2325058 | 0.002927057 |
| 101256329 | LOC101256329 | 2.137629874 | 0.007922478 |
| 101256345 | LOC101256345 | 2.092204012 | 3.42E-05 |
| 101256361 | WDR57 | 1.306026607 | 0.001276447 |
| 101256375 | ABCF1 | 2.228614615 | 5.36E-06 |
| 101256384 | LOC101256384 | 2.206932579 | 6.69E-10 |
| 101256404 | LOC101256404 | 2.505233071 | 2.37E-08 |
| 101256409 | USP | 7.726128647 | 7.77E-13 |
| 101256411 | LOC101256411 | 2.433932682 | 2.93E-05 |
| 101256412 | LOC101256412 | 1.272534562 | 0.001663603 |
| 101256424 | MYB49 | 6.345750824 | 0.003562213 |
| 101256440 | LOC101256440 | 1.312394436 | 0.002326127 |
| 101256461 | LOC101256461 | 1.514345132 | 4.69E-05 |
| 101256463 | LOC101256463 | 1.864914523 | 0.003387173 |
| 101256483 | LOC101256483 | 3.37360067 | 1.18E-11 |
| 101256484 | LOC101256484 | 4.435438457 | 7.89E-07 |
| 101256499 | DDTFR5 | 1.04571905 | 0.003437197 |
| 101256569 | LOC101256569 | 1.330414373 | 0.005716395 |
| 101256573 | LOC101256573 | 1.384619301 | 2.85E-05 |
| 101256574 | LOC101256574 | 3.898734185 | 3.85E-04 |
| 101256649 | LOC101256649 | 3.138969399 | 0.003252227 |
| 101256661 | LOC101256661 | 1.583798918 | 2.36E-04 |
| 101256675 | LOC101256675 | 1.26149337 | 0.00115048 |
| 101256680 | LOC101256680 | 1.169415458 | 0.006018138 |
| 101256683 | LOC101256683 | 1.195462571 | 0.006898391 |
| 101256719 | LOC101256719 | 6.027774738 | 1.09E-16 |
| 101256758 | LOC101256758 | 2.518587937 | 0.003549549 |
| 101256765 | LOC101256765 | 5.227410706 | 2.38E-06 |
| 101256774 | LOC101256774 | 1.899934144 | 1.25E-09 |
| 101256782 | ABCA7 | 5.358115476 | 0.001391779 |
| 101256789 | LOC101256789 | 6.14922516 | 5.82E-05 |
| 101256805 | LOC101256805 | 1.241578053 | 0.005279778 |
| 101256831 | LOC101256831 | 1.528754078 | 1.38E-05 |
| 101256841 | LOC101256841 | 1.348565313 | 5.49E-04 |
| 101256859 | LOC101256859 | 1.368018629 | 2.57E-05 |
| 101256949 | LOC101256949 | 1.218840645 | 0.001495806 |
| 101256975 | LOC101256975 | 2.245260976 | 3.65E-14 |
| 101256986 | LOC101256986 | 5.921555627 | 1.72E-10 |
| 101256995 | LOC101256995 | 1.215643865 | 0.007966453 |
| 101257050 | LOC101257050 | 1.322237105 | 0.008882923 |
| 101257073 | LOC101257073 | 4.363006754 | 2.57E-04 |
| 101257082 | LOC101257082 | 1.014113407 | 0.003035176 |
| 101257085 | LOC101257085 | 1.543872411 | 8.99E-06 |
| 101257088 | LOC101257088 | 1.156146253 | 6.58E-04 |
| 101257097 | LOC101257097 | 2.081420611 | 9.50E-11 |
| 101257109 | ELIP | 3.291008554 | 3.74E-15 |
| 101257201 | CRF3 | 1.131204796 | 0.001174412 |
| 101257239 | LOC101257239 | 3.382347119 | 6.77E-09 |
| 101257246 | LOC101257246 | 1.267071029 | 0.007731649 |
| 101257248 | LOC101257248 | 1.74759547 | 1.70E-04 |
| 101257261 | LOC101257261 | 3.380838502 | 1.02E-07 |
| 101257273 | LOC101257273 | 3.710164121 | 0.003137538 |
| 101257277 | LOC101257277 | 1.532248775 | 0.002920765 |
| 101257284 | LOC101257284 | 2.57803429 | 1.40E-04 |
| 101257319 | LOC101257319 | 1.749340802 | 0.001990712 |
| 101257322 | LOC101257322 | 5.668867473 | 2.19E-05 |
| 101257330 | LOC101257330 | 3.580831961 | 8.08E-04 |
| 101257357 | LOC101257357 | 6.311177393 | 0.001249814 |
| 101257377 | LOC101257377 | 2.776327437 | 2.73E-09 |
| 101257407 | LOC101257407 | 2.141882459 | 1.19E-05 |
| 101257426 | LOC101257426 | 3.149620553 | 4.92E-12 |
| 101257456 | LOC101257456 | 2.828949666 | 0.006068846 |
| 101257460 | LOC101257460 | 1.079520064 | 1.73E-04 |
| 101257472 | LOC101257472 | 4.511713804 | 8.23E-05 |
| 101257476 | LOC101257476 | 4.157314352 | 2.38E-04 |
| 101257483 | LOC101257483 | 2.027292898 | 0.002391955 |
| 101257494 | LOC101257494 | 3.376647038 | 2.33E-08 |
| 101257513 | LOC101257513 | 2.916142909 | 0.001038218 |
| 101257524 | LOC101257524 | 2.684975297 | 0.004685912 |
| 101257533 | LOC101257533 | 1.887016502 | 0.003206481 |
| 101257542 | LOC101257542 | 3.127095208 | 6.60E-13 |
| 101257555 | LOC101257555 | 2.021992595 | 3.88E-04 |
| 101257564 | LOC101257564 | 4.413305354 | 1.17E-13 |
| 101257583 | LOC101257583 | 2.822088312 | 1.13E-05 |
| 101257588 | LOC101257588 | 3.113994988 | 3.34E-09 |
| 101257598 | LOC101257598 | 3.851517625 | 2.44E-05 |
| 101257604 | LOC101257604 | 2.009167975 | 8.65E-05 |
| 101257641 | LOC101257641 | 2.915219502 | 5.56E-04 |
| 101257656 | LOC101257656 | 1.336735238 | 3.52E-05 |
| 101257678 | LOC101257678 | 1.675173244 | 1.46E-04 |
| 101257694 | LOC101257694 | 2.007716818 | 0.001303281 |
| 101257705 | ARS1 | 3.368016679 | 7.77E-08 |
| 101257721 | LOC101257721 | 1.819020475 | 2.02E-04 |
| 101257730 | LOC101257730 | 3.272133577 | 5.71E-07 |
| 101257759 | LOC101257759 | 5.969644823 | 8.27E-17 |
| 101257764 | LOC101257764 | 3.068373597 | 3.28E-15 |
| 101257767 | LOC101257767 | 1.887586756 | 1.28E-07 |
| 101257775 | LOC101257775 | 1.564456957 | 0.001334859 |
| 101257782 | LOC101257782 | 1.017080129 | 0.003259029 |
| 101257783 | LOC101257783 | 3.155688659 | 4.55E-05 |
| 101257787 | LOC101257787 | 1.132361405 | 0.002802033 |
| 101257806 | LOC101257806 | 1.750717189 | 6.51E-05 |
| 101257837 | LOC101257837 | 1.288184261 | 0.003211824 |
| 101257866 | LOC101257866 | 4.748612853 | 1.48E-08 |
| 101257878 | LOC101257878 | 3.290010877 | 5.78E-05 |
| 101257895 | LOC101257895 | 6.262477534 | 1.65E-10 |
| 101257912 | LOC101257912 | 1.719777153 | 8.16E-04 |
| 101257914 | LOC101257914 | 1.36628223 | 0.006728084 |
| 101257929 | LOC101257929 | 4.448267023 | 3.03E-07 |
| 101257933 | LOC101257933 | 1.233504906 | 0.004101006 |
| 101257939 | FEY | 1.406918845 | 6.37E-04 |
| 101257948 | LOC101257948 | 1.419644072 | 3.70E-05 |
| 101257951 | LOC101257951 | 1.87719555 | 1.58E-05 |
| 101257958 | LOC101257958 | 1.235347701 | 0.00593781 |
| 101257964 | LOC101257964 | 1.432903055 | 8.64E-04 |
| 101257978 | LOC101257978 | 1.859595719 | 0.002757243 |
| 101257980 | LOC101257980 | 1.134625486 | 0.003888302 |
| 101257984 | LOC101257984 | 2.016387043 | 6.80E-04 |
| 101258039 | LOC101258039 | 3.425239025 | 0.001884627 |
| 101258053 | LOC101258053 | 1.401389315 | 6.07E-04 |
| 101258059 | LOC101258059 | 1.400348667 | 7.97E-05 |
| 101258061 | LOC101258061 | 1.111499269 | 0.005562082 |
| 101258068 | LOC101258068 | 1.478104693 | 0.008144494 |
| 101258069 | LOC101258069 | 1.305353706 | 0.005303489 |
| 101258082 | LOC101258082 | 4.179761742 | 7.38E-12 |
| 101258116 | LOC101258116 | 2.146460937 | 3.20E-05 |
| 101258159 | LOC101258159 | 6.68542438 | 7.06E-15 |
| 101258188 | LOC101258188 | 6.620076718 | 1.47E-04 |
| 101258189 | Sbt4e | 3.430054427 | 3.23E-04 |
| 101258197 | LOC101258197 | 1.308225637 | 0.006994354 |
| 101258305 | LOC101258305 | 1.479481918 | 0.008927618 |
| 101258347 | LOC101258347 | 2.575775508 | 1.03E-05 |
| 101258351 | ABCB21 | 1.96475367 | 2.94E-05 |
| 101258355 | LOC101258355 | 2.149570338 | 2.58E-04 |
| 101258361 | LOC101258361 | 2.678672434 | 6.26E-04 |
| 101258366 | LOC101258366 | 3.360835788 | 1.41E-04 |
| 101258399 | LOC101258399 | 1.760219994 | 0.003429634 |
| 101258401 | LOC101258401 | 1.109153127 | 0.001804931 |
| 101258424 | LOC101258424 | 1.520294816 | 0.004732994 |
| 101258448 | LOC101258448 | 2.689484358 | 8.12E-06 |
| 101258455 | LOC101258455 | 2.440209826 | 0.003132936 |
| 101258466 | LOC101258466 | 1.778609 | 0.003903823 |
| 101258488 | LOC101258488 | 1.130269691 | 0.002503125 |
| 101258504 | LOC101258504 | 1.720168842 | 0.0017195 |
| 101258518 | LOC101258518 | 1.499810167 | 7.06E-04 |
| 101258529 | LOC101258529 | 5.284596003 | 3.89E-09 |
| 101258554 | LOC101258554 | 2.062184747 | 3.29E-05 |
| 101258610 | LOC101258610 | 1.236819239 | 0.006944702 |
| 101258637 | LOC101258637 | 1.591333128 | 0.001329611 |
| 101258649 | LOC101258649 | 1.137388167 | 0.00704131 |
| 101258707 | LOC101258707 | 3.127512795 | 1.42E-04 |
| 101258710 | LOC101258710 | 2.292882312 | 1.01E-04 |
| 101258716 | LOC101258716 | 1.384451192 | 3.15E-05 |
| 101258720 | LOC101258720 | 6.17410092 | 4.68E-06 |
| 101258757 | LOC101258757 | 1.824312025 | 8.36E-06 |
| 101258773 | LOC101258773 | 1.424497563 | 0.001727286 |
| 101258787 | LOC101258787 | 1.434336577 | 0.003364957 |
| 101258806 | LOC101258806 | 2.237351027 | 0.002050368 |
| 101258808 | LOC101258808 | 1.833700704 | 0.00569313 |
| 101258820 | LOC101258820 | 1.637317488 | 7.15E-06 |
| 101258825 | LOC101258825 | 1.960222381 | 7.26E-07 |
| 101258845 | LOC101258845 | 1.095432677 | 0.006466 |
| 101258855 | TLOG1 | 3.732595452 | 8.15E-14 |
| 101258875 | LOC101258875 | 1.140140845 | 0.001628757 |
| 101258876 | LOC101258876 | 3.281960807 | 1.98E-05 |
| 101258886 | LOC101258886 | 1.06911526 | 0.003742465 |
| 101258887 | LOC101258887 | 3.785424794 | 3.33E-05 |
| 101258901 | LOC101258901 | 2.16609696 | 0.003605096 |
| 101258947 | LOC101258947 | 1.51754156 | 6.20E-06 |
| 101258970 | LOC101258970 | 2.327019424 | 1.58E-08 |
| 101258984 | LOC101258984 | 2.542899475 | 5.19E-06 |
| 101258987 | LOC101258987 | 3.23463715 | 1.29E-09 |
| 101259010 | LOC101259010 | 2.451725712 | 1.43E-05 |
| 101259033 | LOC101259033 | 2.522255493 | 7.01E-05 |
| 101259046 | LOC101259046 | 2.639051467 | 2.28E-04 |
| 101259059 | LOC101259059 | 2.729309216 | 0.008786002 |
| 101259089 | LOC101259089 | 1.855662205 | 8.28E-05 |
| 101259100 | LOC101259100 | 1.413472987 | 3.10E-04 |
| 101259104 | LOC101259104 | 1.861239915 | 1.44E-05 |
| 101259125 | LOC101259125 | 1.330598137 | 0.005108909 |
| 101259170 | LOC101259170 | 1.724842809 | 0.006281367 |
| 101259171 | LOC101259171 | 3.744062528 | 7.01E-07 |
| 101259175 | LOC101259175 | 6.921994907 | 4.91E-17 |
| 101259184 | LOC101259184 | 1.357616821 | 0.007173222 |
| 101259206 | LOC101259206 | 2.364288703 | 0.008423559 |
| 101259222 | LOC101259222 | 3.419653773 | 3.70E-05 |
| 101259235 | LOC101259235 | 4.116445569 | 3.35E-05 |
| 101259241 | LOC101259241 | 3.233562886 | 1.81E-13 |
| 101259285 | LOC101259285 | 1.248669805 | 0.003261265 |
| 101259288 | LOC101259288 | 1.112619027 | 0.006109312 |
| 101259293 | LOC101259293 | 2.373307463 | 7.14E-05 |
| 101259322 | LOC101259322 | 1.096309683 | 1.33E-04 |
| 101259333 | LOC101259333 | 2.152409521 | 2.81E-04 |
| 101259348 | LOC101259348 | 2.626354831 | 0.002769583 |
| 101259349 | PHYF | 1.266055732 | 0.001003527 |
| 101259361 | LOC101259361 | 5.142504842 | 2.81E-07 |
| 101259390 | LOC101259390 | 2.957182504 | 1.30E-05 |
| 101259391 | LOC101259391 | 3.663068754 | 1.24E-15 |
| 101259402 | LOC101259402 | 1.686778907 | 0.001491471 |
| 101259430 | LOC101259430 | 2.60357374 | 0.001822589 |
| 101259437 | LOC101259437 | 3.64569415 | 9.07E-07 |
| 101259438 | LOC101259438 | 2.065971262 | 2.14E-05 |
| 101259441 | LOC101259441 | 6.057937463 | 1.17E-05 |
| 101259456 | LOC101259456 | 3.591506386 | 3.82E-08 |
| 101259469 | LOC101259469 | 2.555027557 | 1.17E-05 |
| 101259504 | LOC101259504 | 1.74545779 | 0.005798729 |
| 101259518 | LOC101259518 | 1.50124153 | 0.006484472 |
| 101259536 | LOC101259536 | 2.110095851 | 2.14E-04 |
| 101259675 | LOC101259675 | 2.073171484 | 9.06E-05 |
| 101259682 | LOC101259682 | 2.961709686 | 4.90E-04 |
| 101259708 | LOC101259708 | 4.601530017 | 2.19E-05 |
| 101259744 | LOC101259744 | 3.995585576 | 3.23E-09 |
| 101259757 | LOC101259757 | 1.461017968 | 0.007066521 |
| 101259778 | LOC101259778 | 1.044624787 | 0.002816535 |
| 101259784 | LOC101259784 | 3.673425253 | 0.001062831 |
| 101259820 | LOC101259820 | 2.611993905 | 7.41E-07 |
| 101259835 | LOC101259835 | 2.258770778 | 8.17E-08 |
| 101259838 | LOC101259838 | 2.682188388 | 1.04E-08 |
| 101259839 | LOC101259839 | 2.078739341 | 4.91E-06 |
| 101259841 | LOC101259841 | 1.029218208 | 0.002573762 |
| 101259849 | LOC101259849 | 1.068294897 | 3.23E-04 |
| 101259860 | LOC101259860 | 1.133770764 | 0.002282523 |
| 101259887 | LOC101259887 | 1.241201934 | 7.68E-04 |
| 101259889 | LOC101259889 | 1.479797525 | 2.91E-04 |
| 101259898 | LOC101259898 | 3.32237439 | 0.00115131 |
| 101259903 | LOC101259903 | 5.028235728 | 6.01E-09 |
| 101259953 | LOC101259953 | 2.358210629 | 6.74E-04 |
| 101259958 | LOC101259958 | 1.78722589 | 0.001562472 |
| 101259973 | LOC101259973 | 1.77231046 | 7.75E-05 |
| 101259986 | LOC101259986 | 2.135353014 | 2.89E-06 |
| 101260001 | LOC101260001 | 1.200266715 | 0.00249253 |
| 101260003 | LOC101260003 | 1.406996267 | 4.66E-05 |
| 101260038 | LOC101260038 | 2.114372251 | 4.47E-10 |
| 101260051 | LOC101260051 | 4.003154257 | 0.002712674 |
| 101260068 | LOC101260068 | 1.421654484 | 9.28E-04 |
| 101260081 | LOC101260081 | 1.181915103 | 0.002814059 |
| 101260143 | HSP90 | 7.664504022 | 4.12E-11 |
| 101260153 | LOC101260153 | 2.70995081 | 6.19E-05 |
| 101260158 | G | 1.507222313 | 0.002476165 |
| 101260177 | ABCG47 | 6.583549101 | 2.20E-06 |
| 101260181 | LOC101260181 | 1.286173313 | 0.001752203 |
| 101260214 | LOC101260214 | 1.855100695 | 6.22E-07 |
| 101260227 | LOC101260227 | 1.206149728 | 0.00238893 |
| 101260261 | LOC101260261 | 3.308309394 | 2.54E-11 |
| 101260271 | LOC101260271 | 2.102571224 | 9.69E-05 |
| 101260278 | LOC101260278 | 1.547408732 | 0.002367959 |
| 101260285 | LOC101260285 | 1.021087742 | 7.03E-04 |
| 101260288 | LOC101260288 | 2.453170663 | 9.33E-06 |
| 101260293 | LOC101260293 | 1.22208289 | 8.59E-04 |
| 101260320 | LOC101260320 | 4.338573037 | 2.10E-06 |
| 101260326 | LOC101260326 | 2.219044271 | 3.09E-05 |
| 101260339 | LOC101260339 | 1.569357429 | 8.14E-04 |
| 101260344 | LOC101260344 | 3.796549586 | 4.49E-05 |
| 101260345 | ABCG40 | 3.778076388 | 0.002534399 |
| 101260372 | LOC101260372 | 1.208910763 | 0.002222054 |
| 101260375 | LOC101260375 | 1.114593628 | 7.01E-04 |
| 101260386 | SAP9 | 2.308554575 | 3.98E-10 |
| 101260391 | LOC101260391 | 2.095392074 | 0.008481838 |
| 101260393 | LOC101260393 | 2.147599479 | 5.65E-06 |
| 101260400 | LOC101260400 | 3.701271011 | 4.54E-06 |
| 101260415 | LOC101260415 | 3.892523538 | 7.46E-10 |
| 101260429 | LOC101260429 | 1.432647706 | 1.79E-04 |
| 101260431 | LOC101260431 | 3.17578261 | 3.20E-06 |
| 101260438 | LOC101260438 | 6.698877704 | 4.43E-04 |
| 101260448 | LOC101260448 | 9.672159002 | 8.98E-09 |
| 101260461 | LOC101260461 | 5.086768847 | 1.09E-06 |
| 101260468 | LOC101260468 | 2.58380817 | 1.99E-05 |
| 101260475 | LOC101260475 | 3.924393469 | 2.80E-06 |
| 101260481 | LOC101260481 | 4.349803221 | 8.22E-10 |
| 101260490 | LOC101260490 | 1.667059436 | 3.76E-04 |
| 101260499 | LOC101260499 | 1.289390678 | 0.007110729 |
| 101260528 | LOC101260528 | 1.192130311 | 5.38E-05 |
| 101260532 | LOC101260532 | 1.011268241 | 0.007433554 |
| 101260535 | LOC101260535 | 2.299826118 | 3.13E-07 |
| 101260537 | LOC101260537 | 3.094796995 | 3.29E-10 |
| 101260602 | LOC101260602 | 1.595926928 | 0.007840089 |
| 101260605 | LOC101260605 | 1.082323827 | 5.74E-04 |
| 101260631 | Actin | 1.847568524 | 0.001228265 |
| 101260639 | LOC101260639 | 3.875821404 | 0.001980472 |
| 101260643 | ABCG39 | 4.900808756 | 9.74E-12 |
| 101260685 | LOC101260685 | 2.716636659 | 1.91E-06 |
| 101260688 | LOC101260688 | 1.069616316 | 0.004777314 |
| 101260726 | LOC101260726 | 2.844894662 | 1.96E-08 |
| 101260745 | LOC101260745 | 1.37523107 | 0.004663089 |
| 101260764 | LOC101260764 | 2.063147155 | 4.87E-07 |
| 101260794 | LOC101260794 | 1.692236801 | 8.30E-06 |
| 101260795 | LOC101260795 | 2.930996049 | 5.93E-05 |
| 101260799 | LOC101260799 | 1.767459268 | 4.48E-05 |
| 101260816 | LOC101260816 | 2.408972196 | 3.35E-09 |
| 101260845 | LOC101260845 | 1.137187009 | 8.38E-04 |
| 101260851 | LOC101260851 | 3.85257052 | 2.49E-13 |
| 101260859 | LOC101260859 | 2.429026766 | 3.55E-08 |
| 101260863 | LOC101260863 | 1.963572493 | 1.01E-06 |
| 101260867 | LOC101260867 | 1.443747686 | 0.003207732 |
| 101260881 | LOC101260881 | 1.444873117 | 0.001676186 |
| 101260894 | LOC101260894 | 2.118852137 | 0.002018907 |
| 101260926 | LOC101260926 | 1.125001404 | 0.002313932 |
| 101260940 | FZY4 | 2.417418367 | 2.48E-06 |
| 101260951 | LOC101260951 | 2.622782945 | 1.78E-07 |
| 101260980 | LOC101260980 | 3.407334819 | 0.003189953 |
| 101260987 | LOC101260987 | 2.802952469 | 6.82E-06 |
| 101260988 | LOC101260988 | 2.808418445 | 2.52E-06 |
| 101260996 | LOC101260996 | 3.23139185 | 2.99E-07 |
| 101261000 | LOC101261000 | 2.093905197 | 4.34E-04 |
| 101261024 | LOC101261024 | 2.89616828 | 3.88E-04 |
| 101261040 | LOC101261040 | 1.595807324 | 9.70E-04 |
| 101261044 | LOC101261044 | 1.107562505 | 0.006623391 |
| 101261049 | LOC101261049 | 1.604484256 | 9.71E-05 |
| 101261079 | LOC101261079 | 5.580235987 | 2.51E-09 |
| 101261118 | LOC101261118 | 2.421429379 | 1.16E-04 |
| 101261120 | LOC101261120 | 3.984183998 | 5.59E-09 |
| 101261134 | LOC101261134 | 1.25527972 | 0.002929563 |
| 101261142 | LOC101261142 | 1.708272416 | 0.001501266 |
| 101261180 | LOC101261180 | 2.432823596 | 6.52E-06 |
| 101261181 | UGT73C4 | 3.581666273 | 1.30E-09 |
| 101261193 | LOC101261193 | 5.803658843 | 6.61E-09 |
| 101261220 | LOC101261220 | 3.909858271 | 1.43E-04 |
| 101261239 | STP11 | 4.282279873 | 1.33E-18 |
| 101261250 | LOC101261250 | 1.103653136 | 0.00194618 |
| 101261262 | LOC101261262 | 2.385433958 | 4.89E-04 |
| 101261287 | LOC101261287 | 2.72715961 | 4.09E-10 |
| 101261288 | LOC101261288 | 1.921733542 | 1.19E-07 |
| 101261292 | LOC101261292 | 3.180915562 | 0.002564622 |
| 101261295 | LOC101261295 | 1.479415955 | 2.71E-04 |
| 101261308 | LOC101261308 | 2.458130274 | 0.001115458 |
| 101261342 | LOC101261342 | 4.070270879 | 2.47E-04 |
| 101261376 | LOC101261376 | 1.185871477 | 0.001245407 |
| 101261380 | LOC101261380 | 8.417560835 | 2.94E-08 |
| 101261382 | LOC101261382 | 2.727141909 | 3.46E-08 |
| 101261398 | LOC101261398 | 9.531698661 | 2.04E-04 |
| 101261438 | LOC101261438 | 2.980459639 | 1.41E-04 |
| 101261450 | LOC101261450 | 2.398251198 | 0.001811876 |
| 101261471 | LOC101261471 | 1.959691989 | 0.001928691 |
| 101261478 | LOC101261478 | 5.196661857 | 2.46E-06 |
| 101261512 | LOC101261512 | 10.22938247 | 3.83E-05 |
| 101261529 | LOC101261529 | 2.695151461 | 8.03E-06 |
| 101261571 | LOC101261571 | 1.63929683 | 0.003118212 |
| 101261587 | LOC101261587 | 2.539885486 | 4.37E-04 |
| 101261589 | LOC101261589 | 1.103923056 | 1.21E-04 |
| 101261609 | LOC101261609 | 1.379616128 | 0.005849142 |
| 101261619 | LOC101261619 | 4.329632982 | 3.69E-05 |
| 101261626 | Endo-LE | 1.468948085 | 1.40E-04 |
| 101261650 | LOC101261650 | 6.833938875 | 1.15E-10 |
| 101261658 | FZY6 | 2.458175867 | 4.09E-04 |
| 101261662 | LOC101261662 | 7.552540682 | 4.82E-63 |
| 101261675 | LOC101261675 | 5.54987771 | 2.42E-09 |
| 101261676 | LOC101261676 | 3.103261636 | 7.09E-17 |
| 101261691 | LOC101261691 | 4.331776129 | 1.51E-06 |
| 101261722 | LOC101261722 | 4.548652287 | 1.29E-08 |
| 101261723 | LOC101261723 | 1.681196961 | 2.92E-05 |
| 101261742 | LOC101261742 | 5.029339809 | 7.68E-06 |
| 101261748 | LOC101261748 | 1.491695661 | 0.004160024 |
| 101261757 | LOC101261757 | 1.020806534 | 0.008085565 |
| 101261760 | LOC101261760 | 1.351038455 | 1.28E-05 |
| 101261765 | LOC101261765 | 5.72643958 | 1.85E-07 |
| 101261766 | LOC101261766 | 6.04385685 | 0.007474188 |
| 101261799 | LOC101261799 | 4.131395097 | 8.55E-07 |
| 101261803 | LOC101261803 | 6.761653223 | 4.63E-06 |
| 101261811 | LOC101261811 | 1.948674643 | 4.06E-08 |
| 101261815 | LOC101261815 | 1.073330111 | 8.01E-04 |
| 101261825 | LOC101261825 | 2.94693699 | 0.001390924 |
| 101261842 | LOC101261842 | 1.348668758 | 3.32E-07 |
| 101261850 | LOC101261850 | 1.524021227 | 0.002124981 |
| 101261863 | LOC101261863 | 1.900738383 | 2.61E-05 |
| 101261889 | LOC101261889 | 4.569891856 | 6.41E-05 |
| 101261922 | LOC101261922 | 1.626973701 | 0.00317991 |
| 101261924 | LOC101261924 | 4.089050937 | 3.38E-07 |
| 101262003 | LOC101262003 | 1.666335012 | 0.001454082 |
| 101262010 | LOC101262010 | 1.596429579 | 1.29E-07 |
| 101262012 | LOC101262012 | 5.73245506 | 8.84E-04 |
| 101262018 | LOC101262018 | 1.039455263 | 0.008472416 |
| 101262021 | LOC101262021 | 6.499075937 | 9.41E-11 |
| 101262071 | LOC101262071 | 2.601063913 | 2.61E-04 |
| 101262083 | LOC101262083 | 1.23217297 | 0.00268633 |
| 101262109 | LOC101262109 | 2.174231991 | 4.09E-05 |
| 101262113 | LOC101262113 | 2.25506719 | 3.05E-04 |
| 101262114 | LOC101262114 | 3.246838726 | 0.008403533 |
| 101262119 | LOC101262119 | 1.508009018 | 0.001009282 |
| 101262133 | LOC101262133 | 1.10958233 | 0.003335943 |
| 101262183 | LOC101262183 | 2.471445374 | 0.005950786 |
| 101262188 | LOC101262188 | 3.606345404 | 2.37E-06 |
| 101262201 | LOC101262201 | 3.365491825 | 0.00182561 |
| 101262205 | LOC101262205 | 2.483545444 | 7.32E-04 |
| 101262238 | LOC101262238 | 1.284566276 | 0.002509731 |
| 101262255 | LOC101262255 | 2.415287735 | 1.52E-05 |
| 101262274 | LOC101262274 | 1.600018591 | 0.001360486 |
| 101262285 | LOC101262285 | 2.036771675 | 0.008254475 |
| 101262304 | LOC101262304 | 1.517827 | 3.30E-04 |
| 101262308 | LOC101262308 | 1.229727788 | 3.69E-04 |
| 101262309 | LOC101262309 | 5.15446382 | 3.54E-11 |
| 101262319 | YABBY2a | 1.059400753 | 0.001162673 |
| 101262327 | LOC101262327 | 2.061693277 | 2.34E-07 |
| 101262338 | LOC101262338 | 2.957457128 | 0.009200769 |
| 101262343 | LOC101262343 | 7.366563944 | 1.35E-05 |
| 101262372 | LOC101262372 | 3.103412929 | 1.48E-05 |
| 101262373 | LOC101262373 | 4.317671808 | 3.30E-11 |
| 101262378 | LOC101262378 | 1.523837845 | 0.001721389 |
| 101262398 | LOC101262398 | 1.297622057 | 2.93E-05 |
| 101262431 | LOC101262431 | 3.383409379 | 0.004783195 |
| 101262446 | LOC101262446 | 2.256016054 | 1.45E-04 |
| 101262464 | LOC101262464 | 1.659309765 | 3.21E-05 |
| 101262486 | LOC101262486 | 3.459709396 | 5.96E-07 |
| 101262517 | LOC101262517 | 1.760954535 | 0.002092928 |
| 101262533 | LOC101262533 | 6.967456457 | 1.98E-10 |
| 101262591 | OSC2 | 3.037877988 | 0.002089676 |
| 101262601 | LOC101262601 | 1.863485928 | 0.008252027 |
| 101262618 | LOC101262618 | 3.559041192 | 3.46E-05 |
| 101262621 | LOC101262621 | 2.321868945 | 0.004511262 |
| 101262627 | LOC101262627 | 4.084646715 | 0.004855328 |
| 101262634 | LOC101262634 | 1.135812336 | 0.001929834 |
| 101262637 | LOC101262637 | 1.669366543 | 3.46E-07 |
| 101262648 | LOC101262648 | 2.81627516 | 9.42E-10 |
| 101262668 | LOC101262668 | 2.881977679 | 2.35E-04 |
| 101262697 | LOC101262697 | 3.827248407 | 2.17E-16 |
| 101262706 | LOC101262706 | 5.043065503 | 0.005610013 |
| 101262709 | LOC101262709 | 4.18820324 | 6.27E-09 |
| 101262710 | LOC101262710 | 1.412525695 | 0.005785986 |
| 101262730 | LOC101262730 | 1.17607187 | 2.03E-04 |
| 101262732 | ALDH11A3a | 1.958155638 | 7.51E-05 |
| 101262744 | LOC101262744 | 2.373650597 | 1.21E-05 |
| 101262760 | LOC101262760 | 1.032048708 | 0.002061573 |
| 101262772 | LOC101262772 | 1.355870897 | 3.85E-04 |
| 101262778 | LOC101262778 | 1.211601368 | 0.003102202 |
| 101262831 | LOC101262831 | 1.208629079 | 0.006160562 |
| 101262857 | LOC101262857 | 1.043378979 | 0.008845453 |
| 101262858 | LOC101262858 | 1.671493078 | 1.40E-05 |
| 101262866 | LOC101262866 | 2.170239377 | 0.00848465 |
| 101262875 | LOC101262875 | 2.236363213 | 9.71E-05 |
| 101262920 | LOC101262920 | 2.14746812 | 2.90E-05 |
| 101262924 | LOC101262924 | 1.282350036 | 6.46E-04 |
| 101262956 | LOC101262956 | 1.129924438 | 0.009170272 |
| 101262960 | LOC101262960 | 2.167122576 | 5.29E-08 |
| 101262976 | LOC101262976 | 2.987733983 | 3.16E-05 |
| 101262987 | LOC101262987 | 2.043083955 | 3.97E-07 |
| 101263011 | LOC101263011 | 3.258082704 | 1.05E-05 |
| 101263015 | LOC101263015 | 5.641159534 | 6.66E-05 |
| 101263038 | LOC101263038 | 1.409535444 | 0.008160237 |
| 101263046 | LOC101263046 | 2.293904084 | 1.87E-06 |
| 101263137 | LOC101263137 | 2.351277306 | 3.65E-05 |
| 101263156 | LOC101263156 | 1.558134589 | 3.18E-05 |
| 101263162 | LOC101263162 | 1.647930449 | 2.38E-04 |
| 101263166 | LOC101263166 | 1.884002525 | 5.85E-05 |
| 101263206 | LOC101263206 | 1.634437223 | 0.004233043 |
| 101263232 | LOC101263232 | 1.348239891 | 5.31E-04 |
| 101263236 | LOC101263236 | 1.726984274 | 3.05E-04 |
| 101263241 | LOC101263241 | 1.013393409 | 0.006694856 |
| 101263261 | Tom52 | 1.169386015 | 0.007793756 |
| 101263281 | LOC101263281 | 1.028190148 | 2.51E-04 |
| 101263285 | LOC101263285 | 1.964336808 | 4.95E-06 |
| 101263312 | LOC101263312 | 1.030494515 | 0.009025428 |
| 101263322 | LOC101263322 | 1.090780898 | 0.00438554 |
| 101263360 | LOC101263360 | 2.753146825 | 2.12E-05 |
| 101263372 | LOC101263372 | 6.590707211 | 0.001056556 |
| 101263379 | LOC101263379 | 2.585504563 | 1.92E-04 |
| 101263446 | LOC101263446 | 1.275545368 | 0.001412397 |
| 101263453 | LOC101263453 | 2.006643964 | 1.03E-06 |
| 101263487 | LOC101263487 | 1.865978429 | 0.001104257 |
| 101263500 | LOC101263500 | 1.769455807 | 0.002448306 |
| 101263535 | LOC101263535 | 7.880652763 | 2.03E-13 |
| 101263542 | LOC101263542 | 4.280667094 | 6.58E-15 |
| 101263633 | LOC101263633 | 1.689282405 | 0.003775284 |
| 101263667 | LOC101263667 | 2.619632938 | 1.82E-04 |
| 101263679 | LOC101263679 | 4.776152821 | 5.23E-08 |
| 101263683 | LOC101263683 | 1.264910327 | 0.005133135 |
| 101263689 | LOC101263689 | 1.637601137 | 2.05E-04 |
| 101263715 | LOC101263715 | 7.988589297 | 9.68E-15 |
| 101263717 | LOC101263717 | 1.685284234 | 1.43E-05 |
| 101263738 | LOC101263738 | 1.304958575 | 0.003670205 |
| 101263779 | LOC101263779 | 1.166405154 | 0.008827538 |
| 101263800 | Krp3 | 1.959052809 | 4.86E-04 |
| 101263832 | LOC101263832 | 4.178822528 | 1.68E-04 |
| 101263858 | LOC101263858 | 2.505890844 | 3.69E-08 |
| 101263871 | LOC101263871 | 2.426611113 | 8.65E-08 |
| 101263881 | LOC101263881 | 6.955404296 | 3.58E-04 |
| 101263891 | LOC101263891 | 2.375424877 | 0.001765635 |
| 101263899 | ERF-D5 | 2.946314002 | 0.002730423 |
| 101263919 | LOC101263919 | 2.116737088 | 9.42E-04 |
| 101263944 | LOC101263944 | 2.047539577 | 2.43E-04 |
| 101263951 | LOC101263951 | 2.193217509 | 4.49E-05 |
| 101263953 | LOC101263953 | 1.30075544 | 0.004789746 |
| 101263965 | LOC101263965 | 4.305180071 | 3.96E-07 |
| 101263972 | LOC101263972 | 10.16581286 | 8.70E-05 |
| 101263990 | LOC101263990 | 1.873453542 | 2.43E-09 |
| 101263993 | LOC101263993 | 2.090831845 | 0.001355919 |
| 101264030 | LOC101264030 | 2.209865123 | 1.90E-05 |
| 101264034 | LOC101264034 | 1.065069213 | 0.003321674 |
| 101264066 | LOC101264066 | 4.033142514 | 5.97E-07 |
| 101264085 | LOC101264085 | 5.355649733 | 1.85E-10 |
| 101264089 | PMT6 | 3.427805259 | 7.99E-04 |
| 101264098 | LOC101264098 | 4.634502596 | 1.64E-08 |
| 101264106 | LOC101264106 | 1.099304576 | 0.00121511 |
| 101264117 | LOC101264117 | 1.722811549 | 5.20E-05 |
| 101264128 | LOC101264128 | 1.617744279 | 0.008166146 |
| 101264141 | LOC101264141 | 2.14293913 | 2.09E-06 |
| 101264148 | LOC101264148 | 2.151647679 | 9.56E-04 |
| 101264161 | LOC101264161 | 5.024208242 | 0.004786694 |
| 101264176 | LOC101264176 | 1.443585948 | 0.001303471 |
| 101264183 | LOC101264183 | 4.646642749 | 4.23E-06 |
| 101264192 | LOC101264192 | 1.990982619 | 1.12E-04 |
| 101264200 | LOC101264200 | 2.97651125 | 5.80E-05 |
| 101264216 | LOC101264216 | 1.691109971 | 0.001189893 |
| 101264225 | LOC101264225 | 2.504027138 | 1.93E-06 |
| 101264273 | LOC101264273 | 1.349849361 | 0.002220321 |
| 101264292 | LOC101264292 | 3.993138032 | 3.67E-17 |
| 101264310 | LOC101264310 | 1.051341097 | 0.001114647 |
| 101264319 | LOC101264319 | 1.914734947 | 4.60E-04 |
| 101264330 | LOC101264330 | 1.929192605 | 2.21E-04 |
| 101264380 | LOC101264380 | 5.014120856 | 2.10E-20 |
| 101264390 | LOC101264390 | 2.473293137 | 7.96E-12 |
| 101264400 | LOC101264400 | 5.414848969 | 4.07E-05 |
| 101264419 | LOC101264419 | 3.340040406 | 9.31E-06 |
| 101264425 | LOC101264425 | 5.085405258 | 8.73E-09 |
| 101264467 | LOC101264467 | 1.449810257 | 5.08E-04 |
| 101264486 | LOC101264486 | 1.675361446 | 3.47E-04 |
| 101264502 | LOC101264502 | 1.306510559 | 0.005723889 |
| 101264518 | LOC101264518 | 1.994052348 | 5.18E-07 |
| 101264545 | LOC101264545 | 3.838555288 | 0.004321705 |
| 101264550 | LOC101264550 | 5.088804836 | 2.52E-09 |
| 101264571 | LOC101264571 | 1.096458903 | 0.00364261 |
| 101264574 | LOC101264574 | 7.571858316 | 1.12E-11 |
| 101264576 | LOC101264576 | 2.845420469 | 2.29E-11 |
| 101264605 | PIP1-7 | 2.395016752 | 8.23E-05 |
| 101264611 | LOC101264611 | 1.220396746 | 4.54E-04 |
| 101264614 | LOC101264614 | 2.095690551 | 4.46E-04 |
| 101264668 | LOC101264668 | 1.291462311 | 0.002360631 |
| 101264671 | LOC101264671 | 6.085067074 | 1.03E-18 |
| 101264700 | LOC101264700 | 1.948294547 | 5.75E-05 |
| 101264722 | LOC101264722 | 2.115525879 | 9.31E-09 |
| 101264738 | LOC101264738 | 1.255123523 | 1.12E-04 |
| 101264761 | LOC101264761 | 1.528233187 | 1.27E-04 |
| 101264776 | LOC101264776 | 1.834434724 | 4.74E-07 |
| 101264783 | LOC101264783 | 5.88010496 | 8.36E-11 |
| 101264787 | LOC101264787 | 3.024772577 | 1.09E-21 |
| 101264863 | LOC101264863 | 3.993839586 | 9.02E-16 |
| 101264876 | LOC101264876 | 3.666118884 | 6.21E-04 |
| 101264947 | LOC101264947 | 1.804458696 | 4.35E-06 |
| 101264951 | LOC101264951 | 1.691440316 | 7.57E-04 |
| 101265071 | LOC101265071 | 1.405749632 | 0.009222881 |
| 101265084 | LOC101265084 | 3.11067544 | 5.03E-09 |
| 101265088 | LOC101265088 | 1.852401632 | 3.75E-06 |
| 101265109 | LOC101265109 | 1.03088596 | 0.007592716 |
| 101265114 | LOC101265114 | 2.12752961 | 0.004726776 |
| 101265142 | LOC101265142 | 1.419875322 | 9.67E-05 |
| 101265187 | LOC101265187 | 1.66074557 | 0.006712243 |
| 101265192 | LOC101265192 | 1.140018489 | 0.007723255 |
| 101265227 | LOC101265227 | 2.719904448 | 1.40E-07 |
| 101265235 | LOC101265235 | 1.449650937 | 0.001234269 |
| 101265236 | LOC101265236 | 2.15280397 | 4.10E-06 |
| 101265243 | LOC101265243 | 3.243242939 | 8.21E-04 |
| 101265245 | LOC101265245 | 1.274217263 | 0.007126001 |
| 101265259 | LOC101265259 | 1.591646352 | 1.77E-04 |
| 101265260 | LOC101265260 | 2.29068941 | 9.05E-07 |
| 101265282 | AMT1-1 | 2.565940675 | 2.29E-04 |
| 101265293 | LOC101265293 | 4.136699097 | 5.63E-18 |
| 101265294 | LOC101265294 | 2.113566458 | 2.78E-04 |
| 101265303 | LOC101265303 | 1.705195101 | 2.81E-06 |
| 101265309 | LOC101265309 | 2.007186463 | 5.02E-04 |
| 101265329 | LOC101265329 | 1.716896639 | 0.005658269 |
| 101265334 | LOC101265334 | 1.450016573 | 7.43E-05 |
| 101265359 | LOC101265359 | 1.075829424 | 0.003197068 |
| 101265369 | LOC101265369 | 4.872438949 | 2.29E-04 |
| 101265388 | LOC101265388 | 4.292170137 | 1.00E-06 |
| 101265391 | LOC101265391 | 2.195861559 | 3.95E-08 |
| 101265423 | LOC101265423 | 3.329579365 | 9.20E-05 |
| 101265425 | LOC101265425 | 2.509266615 | 0.001176575 |
| 101265426 | LOC101265426 | 1.935882881 | 6.76E-04 |
| 101265453 | ERF-F3 | 1.214242093 | 4.62E-04 |
| 101265475 | LOC101265475 | 1.006341698 | 0.001174558 |
| 101265479 | LOC101265479 | 1.041018829 | 0.001301776 |
| 101265492 | OFP20 | 2.016912602 | 0.002658418 |
| 101265502 | LOC101265502 | 3.21154115 | 8.11E-05 |
| 101265511 | LOC101265511 | 6.224000683 | 5.29E-10 |
| 101265524 | LOC101265524 | 2.67573752 | 1.60E-09 |
| 101265525 | LOC101265525 | 1.55924321 | 8.08E-04 |
| 101265528 | LOC101265528 | 1.445907683 | 0.007534635 |
| 101265539 | LOC101265539 | 4.307550238 | 9.58E-11 |
| 101265545 | LOC101265545 | 1.231349391 | 7.73E-04 |
| 101265548 | LOC101265548 | 2.949184457 | 0.001068189 |
| 101265566 | LOC101265566 | 4.441277908 | 3.04E-07 |
| 101265571 | LOC101265571 | 2.815989458 | 2.54E-05 |
| 101265586 | LOC101265586 | 4.487173884 | 1.84E-12 |
| 101265601 | LOC101265601 | 1.072892365 | 0.005981842 |
| 101265652 | LOC101265652 | 3.259445079 | 8.45E-04 |
| 101265653 | LOC101265653 | 3.155382025 | 2.95E-06 |
| 101265702 | LOC101265702 | 3.05683742 | 4.96E-12 |
| 101265726 | LOC101265726 | 6.00104731 | 3.23E-05 |
| 101265748 | LOC101265748 | 4.005855453 | 0.006950655 |
| 101265772 | LOC101265772 | 6.705900188 | 4.59E-14 |
| 101265793 | LOC101265793 | 1.640918263 | 0.008167743 |
| 101265813 | LOC101265813 | 5.587394927 | 2.12E-09 |
| 101265816 | LOC101265816 | 1.058479867 | 0.007541617 |
| 101265824 | LOC101265824 | 3.655440444 | 8.67E-04 |
| 101265827 | LOC101265827 | 1.030523969 | 0.003407683 |
| 101265851 | LOC101265851 | 7.160844704 | 1.92E-09 |
| 101265872 | LOC101265872 | 1.081605045 | 0.006557333 |
| 101265879 | LOC101265879 | 5.325120726 | 0.004042883 |
| 101265882 | LOC101265882 | 1.498383665 | 0.002709131 |
| 101265888 | LOC101265888 | 2.554133682 | 3.61E-05 |
| 101265918 | LOC101265918 | 2.554420659 | 0.001438436 |
| 101265927 | LOC101265927 | 3.940295824 | 1.49E-09 |
| 101265936 | LOC101265936 | 2.526186743 | 2.17E-05 |
| 101265985 | LOC101265985 | 1.88167919 | 2.69E-06 |
| 101265986 | LOC101265986 | 4.638014513 | 5.37E-14 |
| 101266018 | LOC101266018 | 2.3305418 | 3.68E-07 |
| 101266046 | LOC101266046 | 2.559487214 | 0.0023137 |
| 101266080 | LOC101266080 | 2.729935346 | 9.33E-04 |
| 101266084 | LOC101266084 | 5.613886518 | 2.96E-05 |
| 101266119 | LOC101266119 | 3.83554399 | 2.63E-10 |
| 101266136 | LOC101266136 | 3.705822509 | 7.83E-04 |
| 101266149 | LOC101266149 | 3.223473184 | 0.002000699 |
| 101266150 | LOC101266150 | 2.562558055 | 6.39E-09 |
| 101266155 | LOC101266155 | 4.107487049 | 9.36E-04 |
| 101266188 | LOC101266188 | 1.630273425 | 1.06E-05 |
| 101266192 | LOC101266192 | 1.094916326 | 0.006595749 |
| 101266200 | LOC101266200 | 1.691392856 | 7.80E-04 |
| 101266208 | LOC101266208 | 1.055666981 | 0.001027012 |
| 101266219 | LOC101266219 | 1.066254438 | 0.00770581 |
| 101266223 | LOC101266223 | 1.677201441 | 0.007371891 |
| 101266262 | LOC101266262 | 1.023167856 | 0.004095512 |
| 101266273 | LOC101266273 | 1.01534706 | 0.003829808 |
| 101266274 | LOC101266274 | 2.746610106 | 1.36E-05 |
| 101266279 | LOC101266279 | 1.071276725 | 0.001978022 |
| 101266280 | LOC101266280 | 1.164499467 | 7.88E-04 |
| 101266283 | LOC101266283 | 1.305390271 | 2.54E-04 |
| 101266293 | LOC101266293 | 4.539336582 | 2.65E-11 |
| 101266325 | LOC101266325 | 5.554606472 | 1.56E-04 |
| 101266334 | LOC101266334 | 1.5220547 | 1.74E-06 |
| 101266356 | LOC101266356 | 2.197807949 | 1.49E-04 |
| 101266368 | LOC101266368 | 1.181605431 | 0.008497651 |
| 101266410 | LOC101266410 | 2.669876046 | 1.32E-06 |
| 101266462 | LOC101266462 | 1.633384973 | 0.003191984 |
| 101266473 | LOC101266473 | 1.309893601 | 0.00496056 |
| 101266505 | Suv | 1.475767679 | 0.001973906 |
| 101266516 | LOC101266516 | 1.73311821 | 6.88E-06 |
| 101266525 | HSP20-1 | 5.451376738 | 0.003462157 |
| 101266561 | LOC101266561 | 1.760999185 | 1.57E-04 |
| 101266592 | LOC101266592 | 2.04534436 | 9.72E-04 |
| 101266599 | LOC101266599 | 1.142431286 | 0.004924102 |
| 101266612 | ERF-D2 | 3.924416777 | 4.85E-06 |
| 101266615 | LOC101266615 | 1.172189052 | 0.001834519 |
| 101266621 | LOC101266621 | 1.736477327 | 0.002660368 |
| 101266638 | LOC101266638 | 6.15867365 | 6.76E-04 |
| 101266649 | LOC101266649 | 1.497215496 | 0.00348596 |
| 101266652 | LOC101266652 | 2.620359372 | 3.17E-05 |
| 101266656 | LOC101266656 | 1.450781041 | 0.00166327 |
| 101266657 | LOC101266657 | 5.721077442 | 5.45E-19 |
| 101266670 | Lyk9 | 1.526486452 | 2.09E-06 |
| 101266739 | LOC101266739 | 1.143792844 | 0.009028019 |
| 101266750 | LOC101266750 | 2.662758422 | 0.00488605 |
| 101266767 | LOC101266767 | 1.514225274 | 0.009068648 |
| 101266770 | LOC101266770 | 5.450532566 | 1.88E-05 |
| 101266813 | LOC101266813 | 1.481936135 | 6.38E-04 |
| 101266826 | LOC101266826 | 4.994045513 | 0.00798982 |
| 101266831 | LOC101266831 | 1.863757147 | 0.007204881 |
| 101266835 | LOC101266835 | 1.19212413 | 0.004332069 |
| 101266854 | LOC101266854 | 1.263785348 | 0.004663314 |
| 101266879 | LOC101266879 | 7.518113142 | 2.31E-12 |
| 101266885 | LOC101266885 | 2.684013378 | 1.56E-06 |
| 101266929 | LOC101266929 | 2.47747195 | 0.005219444 |
| 101266953 | LOC101266953 | 1.505032035 | 2.96E-04 |
| 101266961 | LOC101266961 | 1.361047902 | 0.002904125 |
| 101266963 | LOC101266963 | 3.184956054 | 1.09E-07 |
| 101266973 | LOC101266973 | 6.650146641 | 4.04E-10 |
| 101267036 | LOC101267036 | 2.540416445 | 2.73E-06 |
| 101267054 | LOC101267054 | 1.083328727 | 0.001193572 |
| 101267078 | LOC101267078 | 4.606474149 | 1.55E-05 |
| 101267080 | LOC101267080 | 1.200227938 | 0.004529592 |
| 101267124 | LOC101267124 | 1.433298261 | 6.91E-04 |
| 101267150 | ABCG1 | 1.949298156 | 2.61E-08 |
| 101267173 | LOC101267173 | 1.830699469 | 5.42E-04 |
| 101267231 | LOC101267231 | 2.092791371 | 3.38E-06 |
| 101267235 | LOC101267235 | 1.475757564 | 4.52E-04 |
| 101267254 | LOC101267254 | 4.473330744 | 3.09E-07 |
| 101267309 | LOC101267309 | 1.732351581 | 9.13E-04 |
| 101267310 | LOC101267310 | 4.973248952 | 6.54E-10 |
| 101267311 | LOC101267311 | 1.370054173 | 0.002379842 |
| 101267336 | LOC101267336 | 1.961091634 | 0.002536242 |
| 101267339 | LOC101267339 | 5.941348392 | 0.001159747 |
| 101267358 | LOC101267358 | 3.833074101 | 2.62E-05 |
| 101267366 | LOC101267366 | 2.470516227 | 1.43E-06 |
| 101267371 | LOC101267371 | 4.024829047 | 0.006387642 |
| 101267385 | LOC101267385 | 2.225531513 | 2.79E-08 |
| 101267393 | LOC101267393 | 1.856442514 | 1.55E-05 |
| 101267457 | LOC101267457 | 1.391261313 | 0.00102887 |
| 101267474 | LOC101267474 | 1.663123255 | 0.003982024 |
| 101267476 | LOC101267476 | 1.781829573 | 0.001330898 |
| 101267521 | LOC101267521 | 2.198734646 | 1.20E-07 |
| 101267526 | LOC101267526 | 1.255789269 | 9.70E-04 |
| 101267559 | LOC101267559 | 2.722521338 | 2.66E-06 |
| 101267605 | LOC101267605 | 2.438269518 | 1.83E-07 |
| 101267615 | LOC101267615 | 1.536919689 | 0.009039939 |
| 101267632 | LOC101267632 | 2.513654828 | 1.29E-05 |
| 101267635 | LOC101267635 | 2.264681368 | 6.22E-05 |
| 101267638 | LOC101267638 | 4.590505406 | 0.008825559 |
| 101267645 | LOC101267645 | 1.204951295 | 0.008883425 |
| 101267654 | LOC101267654 | 1.72822505 | 2.30E-07 |
| 101267670 | LOC101267670 | 1.972186469 | 2.56E-05 |
| 101267688 | LOC101267688 | 1.676095344 | 0.007747589 |
| 101267740 | LOC101267740 | 2.358601317 | 6.48E-07 |
| 101267754 | LOC101267754 | 6.229980575 | 1.46E-04 |
| 101267755 | LOC101267755 | 2.547369282 | 7.29E-04 |
| 101267768 | LOC101267768 | 2.072149961 | 0.002582262 |
| 101267777 | LOC101267777 | 1.032973346 | 0.005112253 |
| 101267819 | LOC101267819 | 2.881494061 | 0.006783495 |
| 101267821 | LOC101267821 | 2.698668397 | 0.00449891 |
| 101267868 | LOC101267868 | 1.083912232 | 0.004528668 |
| 101267920 | LOC101267920 | 4.385161028 | 1.15E-07 |
| 101267932 | LOC101267932 | 4.539749779 | 5.05E-07 |
| 101267949 | LOC101267949 | 5.575402442 | 5.76E-06 |
| 101267951 | PSK2 | 4.268747751 | 2.61E-04 |
| 101267958 | LOC101267958 | 1.177407448 | 9.90E-04 |
| 101267963 | LOC101267963 | 1.304936585 | 1.29E-05 |
| 101267965 | LOC101267965 | 1.631936194 | 1.42E-04 |
| 101267969 | LOC101267969 | 2.432981474 | 1.41E-07 |
| 101267987 | LOC101267987 | 3.964404304 | 2.15E-04 |
| 101267989 | LOC101267989 | 1.360415066 | 1.27E-04 |
| 101268004 | LOC101268004 | 2.287595891 | 4.09E-09 |
| 101268010 | LOC101268010 | 4.812571397 | 6.69E-07 |
| 101268013 | CYP72A14 | 3.108870664 | 3.18E-06 |
| 101268014 | LOC101268014 | 4.466081875 | 2.21E-18 |
| 101268031 | ACO3 | 2.718963003 | 0.007952604 |
| 101268052 | LOC101268052 | 2.781113474 | 0.004571313 |
| 101268104 | LOC101268104 | 2.042635016 | 1.14E-08 |
| 101268114 | LOC101268114 | 1.761885233 | 5.81E-04 |
| 101268116 | LOC101268116 | 1.096548971 | 0.009231863 |
| 101268119 | LOC101268119 | 2.186919158 | 9.55E-04 |
| 101268121 | LOC101268121 | 2.53678747 | 6.02E-06 |
| 101268207 | LOC101268207 | 1.933723192 | 0.00503548 |
| 101268226 | LOC101268226 | 1.445011461 | 0.007756386 |
| 101268230 | LOC101268230 | 2.667551607 | 1.06E-09 |
| 101268235 | LOC101268235 | 3.034276194 | 3.50E-04 |
| 101268257 | LOC101268257 | 4.881406707 | 0.004680944 |
| 101268271 | LOC101268271 | 2.182165999 | 3.04E-07 |
| 101268296 | GAME7 | 1.91053834 | 1.23E-05 |
| 101268306 | LOC101268306 | 1.524985546 | 0.007607833 |
| 101268307 | LOC101268307 | 2.661878896 | 3.03E-06 |
| 101268329 | LOC101268329 | 3.226613614 | 1.40E-08 |
| 101268338 | LOC101268338 | 1.909720684 | 0.001106243 |
| 101268354 | LOC101268354 | 2.93248386 | 1.53E-04 |
| 101268373 | LOC101268373 | 1.86331499 | 0.008882958 |
| 101268394 | LOC101268394 | 2.212599463 | 8.85E-08 |
| 101268396 | LOC101268396 | 2.30791991 | 2.82E-06 |
| 101268402 | LOC101268402 | 2.653247739 | 0.001246121 |
| 101268443 | PSK4 | 2.918240747 | 1.24E-06 |
| 101268451 | LOC101268451 | 1.682803887 | 0.001018787 |
| 101268485 | LOC101268485 | 2.577169169 | 1.02E-10 |
| 101268500 | LOC101268500 | 3.87049801 | 3.76E-13 |
| 101268519 | LOC101268519 | 3.221853967 | 0.006924531 |
| 101268569 | LOC101268569 | 3.158514019 | 4.60E-06 |
| 101268570 | LOC101268570 | 1.643056996 | 0.002070361 |
| 101268608 | LOC101268608 | 2.31549193 | 7.34E-05 |
| 101268612 | LOC101268612 | 1.978493205 | 1.84E-05 |
| 101268624 | GH3-1 | 1.338155146 | 7.05E-04 |
| 101268644 | LOC101268644 | 6.775014124 | 5.12E-06 |
| 101268660 | LOC101268660 | 4.005273964 | 3.72E-07 |
| 101268663 | LOC101268663 | 6.724280679 | 8.61E-07 |
| 101268741 | LOC101268741 | 2.979444699 | 0.001399516 |
| 101268769 | LOC101268769 | 1.089848388 | 0.002151077 |
| 101268779 | LOC101268779 | 3.937628183 | 0.001122617 |
| 101268780 | WRKY46 | 3.986222612 | 7.55E-07 |
| 101268798 | LOC101268798 | 1.02286563 | 0.009263762 |
| 101268836 | LOC101268836 | 1.485445344 | 1.84E-04 |
| 101268871 | LOC101268871 | 9.005471732 | 5.27E-12 |
| 101268888 | LOC101268888 | 2.647459716 | 0.002780161 |
| 101268894 | LOC101268894 | 3.170732095 | 0.004199148 |
| 104644282 | LOC104644282 | 1.859126921 | 0.006781746 |
| 104644301 | LOC104644301 | 2.189597824 | 1.77E-04 |
| 104644406 | LOC104644406 | 3.794495176 | 0.002913739 |
| 104644439 | LOC104644439 | 4.524089562 | 3.15E-14 |
| 104644452 | LOC104644452 | 1.822180918 | 7.70E-04 |
| 104644530 | LOC104644530 | 3.505100038 | 5.96E-06 |
| 104644876 | LOC104644876 | 1.284073344 | 0.007639759 |
| 104644893 | LOC104644893 | 3.219394451 | 0.00185318 |
| 104645115 | LOC104645115 | 6.397967272 | 2.66E-07 |
| 104645116 | LOC104645116 | 6.345416893 | 1.35E-04 |
| 104645134 | LOC104645134 | 4.264596219 | 1.40E-06 |
| 104645419 | LOC104645419 | 2.354373949 | 0.007379682 |
| 104645435 | LOC104645435 | 1.984310848 | 5.94E-04 |
| 104645436 | LOC104645436 | 1.812282229 | 0.001693518 |
| 104645444 | OFP3 | 1.330878386 | 0.00673891 |
| 104645713 | LOC104645713 | 1.885586429 | 0.004757362 |
| 104645726 | LOC104645726 | 1.917079449 | 0.002104115 |
| 104645740 | LOC104645740 | 2.681240187 | 6.05E-05 |
| 104645747 | LOC104645747 | 1.102709583 | 0.002023398 |
| 104645857 | LOC104645857 | 4.067015955 | 4.10E-06 |
| 104645867 | LOC104645867 | 3.24259903 | 0.003768632 |
| 104646018 | LOC104646018 | 7.030297612 | 1.12E-04 |
| 104646046 | LOC104646046 | 2.406983171 | 0.007737056 |
| 104646070 | LOC104646070 | 3.146556648 | 3.94E-05 |
| 104646389 | LOC104646389 | 1.71910799 | 0.002656733 |
| 104646390 | CB5-A | 1.33081074 | 0.00118015 |
| 104646537 | LOC104646537 | 3.784291808 | 1.79E-04 |
| 104646690 | LOC104646690 | 3.660265098 | 0.003799049 |
| 104646739 | LOC104646739 | 3.728406698 | 0.004338428 |
| 104646779 | LOC104646779 | 5.134697652 | 1.69E-05 |
| 104646781 | LOC104646781 | 2.86244864 | 4.26E-07 |
| 104646792 | LOC104646792 | 5.758910293 | 9.71E-04 |
| 104646795 | LOC104646795 | 2.602558554 | 0.002149989 |
| 104647229 | LOC104647229 | 1.872045595 | 0.002906662 |
| 104647281 | LOC104647281 | 3.172367991 | 9.26E-05 |
| 104647346 | LOC104647346 | 2.312253542 | 0.007745193 |
| 104647464 | LOC104647464 | 2.574715442 | 0.004642188 |
| 104647663 | LOC104647663 | 7.004391229 | 2.40E-06 |
| 104647908 | LOC104647908 | 7.628282266 | 3.43E-05 |
| 104647952 | LOC104647952 | 1.31063391 | 7.69E-04 |
| 104648015 | LOC104648015 | 5.7591835 | 6.13E-17 |
| 104648134 | LOC104648134 | 3.815553309 | 0.006487696 |
| 104648390 | LOC104648390 | 1.960302946 | 1.01E-05 |
| 104648396 | LOC104648396 | 2.228377906 | 3.52E-05 |
| 104648547 | LOC104648547 | 7.183980267 | 1.29E-06 |
| 104648558 | LOC104648558 | 3.223450303 | 2.13E-11 |
| 104648615 | LOC104648615 | 3.18458682 | 5.77E-04 |
| 104649042 | LOC104649042 | 4.669748263 | 0.006803256 |
| 104649056 | LOC104649056 | 4.88683205 | 4.63E-04 |
| 104649135 | LOC104649135 | 5.980029147 | 2.92E-04 |
| 104649376 | LOC104649376 | 4.968191991 | 6.46E-05 |
| 104649441 | LOC104649441 | 1.751503212 | 3.27E-05 |
| 104649457 | LOC104649457 | 2.207935411 | 5.74E-05 |
| 104649503 | LOC104649503 | 4.9504283 | 8.78E-24 |
| 104649549 | LOC104649549 | 3.401005751 | 0.005977021 |
| 104649655 | LOC104649655 | 2.148409265 | 5.95E-04 |
| 108281193 | LOC108281193 | 2.944157535 | 1.78E-04 |
| 109118701 | LOC109118701 | 4.511004627 | 4.71E-05 |
| 109118703 | LOC109118703 | 2.605955323 | 1.10E-09 |
| 109118704 | LOC109118704 | 2.30965673 | 4.33E-04 |
| 109119058 | LOC109119058 | 6.423861233 | 9.67E-08 |
| 109119181 | LOC109119181 | 4.751836307 | 2.28E-11 |
| 109119816 | LOC109119816 | 5.268843044 | 1.88E-05 |
| 109119849 | LOC109119849 | 4.049054123 | 3.81E-05 |
| 109120151 | LOC109120151 | 2.202931602 | 0.004047193 |
| 109120299 | LOC109120299 | 1.417008656 | 0.005574401 |
| 109120477 | LOC109120477 | 1.869671837 | 0.006140631 |
| 109120544 | LOC109120544 | 2.735539432 | 1.73E-07 |
| 109120859 | LOC109120859 | 3.729029051 | 1.75E-04 |
| 109121105 | LOC109121105 | 2.481470079 | 1.20E-04 |
| 109121373 | LOC109121373 | 3.389289305 | 6.80E-06 |
| 112940013 | LOC112940013 | 3.393946111 | 1.72E-08 |
| 112940015 | LOC112940015 | 1.809299921 | 4.66E-04 |
| 112940026 | LOC112940026 | 1.464638723 | 0.007313928 |
| 112940071 | LOC112940071 | 2.219105781 | 0.001633212 |
| 112940076 | LOC112940076 | 2.239565149 | 0.006097987 |
| 112940297 | LOC112940297 | 1.728609327 | 2.00E-06 |
| 112940356 | LOC112940356 | 2.467785109 | 4.08E-04 |
| 112940472 | LOC112940472 | 1.863426371 | 7.43E-04 |
| 112940619 | LOC112940619 | 2.288993942 | 0.005871037 |
| 112940639 | LOC112940639 | 4.69283863 | 0.003761263 |
| 112940685 | LOC112940685 | 6.169183283 | 0.004955275 |
| 112941069 | LOC112941069 | 2.49582732 | 4.41E-06 |
| 112941248 | LOC112941248 | 4.278417965 | 3.98E-04 |
| 112941338 | LOC112941338 | 4.5961812 | 2.63E-07 |
| 112941537 | LOC112941537 | 1.870136196 | 9.57E-04 |
| 112941565 | LOC112941565 | 4.424154584 | 9.77E-04 |
| 112941696 | LOC112941696 | 2.949625868 | 1.29E-04 |
| 112941801 | LOC112941801 | 4.097979391 | 3.76E-04 |
| 112941853 | LOC112941853 | 1.48897566 | 0.001322796 |
| 112942068 | LOC112942068 | 3.544883777 | 4.17E-04 |
| 112942144 | LOC112942144 | 4.551156778 | 1.22E-04 |
| 543502 | Wiv-1 | 4.406116639 | 9.99E-07 |
| 543506 | ACO4 | 2.118188286 | 4.18E-06 |
| 543507 | MTSHP | 7.529225315 | 1.03E-10 |
| 543511 | LOC543511 | 2.747938415 | 2.38E-06 |
| 543529 | AAP1 | 1.108223989 | 0.004296609 |
| 543530 | ProT1 | 1.685825382 | 1.39E-04 |
| 543542 | IAA7 | 1.883570659 | 3.94E-05 |
| 543571 | RBOH1 | 1.682282531 | 0.003047459 |
| 543572 | HSP17.4 | 6.193635509 | 4.60E-08 |
| 543581 | LOC543581 | 1.790714706 | 0.001360157 |
| 543582 | EXP2 | 2.637346838 | 0.005522209 |
| 543585 | CAT2 | 2.420344045 | 2.16E-06 |
| 543600 | LOC543600 | 1.150112436 | 0.001692675 |
| 543602 | LOC543602 | 6.395606605 | 1.27E-23 |
| 543605 | LOC543605 | 2.734541562 | 1.17E-06 |
| 543609 | TBG3 | 3.200978939 | 5.97E-07 |
| 543619 | XET2 | 1.843068466 | 0.008195491 |
| 543635 | DDTFR18 | 1.389517006 | 1.10E-04 |
| 543645 | LOC543645 | 3.601400633 | 0.00763008 |
| 543652 | TAO3 | 2.687767415 | 2.47E-04 |
| 543659 | Ve1 | 4.409232994 | 7.77E-08 |
| 543667 | EIF-5A1 | 1.934136646 | 1.45E-04 |
| 543675 | DES | 5.54234255 | 0.003886034 |
| 543677 | LOC543677 | 1.12962377 | 0.00193636 |
| 543688 | Cry1b | 1.863392763 | 7.35E-05 |
| 543693 | BL2 | 1.684274866 | 4.12E-10 |
| 543712 | EREB | 5.260008521 | 5.03E-05 |
| 543731 | Sus3 | 3.407604835 | 5.81E-06 |
| 543736 | TBG4 | 2.723254622 | 3.29E-13 |
| 543749 | Rab11a | 1.052075479 | 4.92E-04 |
| 543767 | T19-8 | 1.727541006 | 8.23E-04 |
| 543771 | NHX1 | 1.805621142 | 0.006454729 |
| 543802 | LOC543802 | 1.716192038 | 0.001742601 |
| 543804 | Bmy3 | 5.688621221 | 1.06E-17 |
| 543807 | Adc1 | 2.556388997 | 0.001684222 |
| 543809 | LOC543809 | 5.739041093 | 3.22E-36 |
| 543810 | LOC543810 | 1.240910772 | 0.007244374 |
| 543812 | LOC543812 | 2.256942051 | 0.002969592 |
| 543813 | LOC543813 | 3.5240289 | 0.00116646 |
| 543814 | Ctu1 | 4.062089423 | 8.73E-08 |
| 543816 | GST-T4 | 3.245536359 | 5.10E-05 |
| 543818 | TomloxE | 3.129109925 | 8.92E-08 |
| 543824 | AOX1a | 4.019834855 | 4.88E-07 |
| 543825 | LOC543825 | 7.068424848 | 3.71E-09 |
| 543831 | GCH1 | 2.061046042 | 2.34E-04 |
| 543837 | PR-5 | 3.010639098 | 1.22E-04 |
| 543846 | Vis1 | 6.304429943 | 1.39E-11 |
| 543848 | HSP17-6 | 2.279149726 | 7.44E-05 |
| 543853 | Xegip | 3.76198331 | 5.39E-04 |
| 543867 | ERF4 | 2.150970123 | 1.86E-04 |
| 543870 | LOC543870 | 1.521297579 | 0.003433338 |
| 543871 | FRO1 | 3.159615193 | 6.08E-04 |
| 543873 | GABA-TP1 | 1.956099069 | 8.76E-08 |
| 543875 | AS1 | 7.857997772 | 5.04E-06 |
| 543876 | GAL83 | 1.430234492 | 0.007706846 |
| 543892 | LOC543892 | 2.470919553 | 9.88E-04 |
| 543895 | LOC543895 | 4.879993969 | 0.005954156 |
| 543902 | Hsp90-1 | 1.562137514 | 0.005490198 |
| 543912 | LOC543912 | 2.010871945 | 2.43E-04 |
| 543913 | XTH5 | 2.587252195 | 1.92E-05 |
| 543939 | NPR1 | 1.392815501 | 2.07E-04 |
| 543951 | TIP | 4.765633754 | 2.10E-08 |
| 543953 | ER1 | 7.609919885 | 1.44E-04 |
| 543964 | PSY2 | 1.335913684 | 0.001195856 |
| 543979 | NP24 | 7.211835772 | 1.61E-05 |
| 543986 | LOC543986 | 4.476165675 | 8.55E-05 |
| 543988 | Psy1 | 3.732532077 | 9.24E-08 |
| 543992 | AI | 1.774430965 | 4.35E-14 |
| 543993 | LOC543993 | 2.502855368 | 0.001104785 |
| 544032 | Cel3 | 1.188992561 | 9.71E-04 |
| 544035 | EXP1 | 3.724403127 | 3.19E-04 |
| 544040 | TVPS41 | 1.29991285 | 0.004799887 |
| 544041 | PTI4 | 2.870439243 | 2.42E-04 |
| 544052 | ACO1 | 5.30842357 | 1.62E-22 |
| 544061 | LOC544061 | 3.945295083 | 3.88E-10 |
| 544069 | PR-P2 | 6.841470448 | 9.29E-15 |
| 544070 | LOC544070 | 2.428710319 | 2.87E-04 |
| 544075 | LOC544075 | 1.514521245 | 1.66E-04 |
| 544082 | TPM-1 | 5.760805613 | 4.28E-10 |
| 544084 | CEVI-1 | 2.580433509 | 3.33E-08 |
| 544086 | PIP1-5 | 1.906525523 | 2.08E-07 |
| 544097 | TLRP | 1.538105995 | 4.80E-05 |
| 544098 | RNALE | 6.005979805 | 7.04E-07 |
| 544103 | Twi1 | 1.928590393 | 0.007606809 |
| 544104 | LCY1 | 2.006319245 | 1.84E-05 |
| 544107 | Cevi19 | 2.760388761 | 6.23E-09 |
| 544110 | AMT1-2 | 5.396759155 | 2.44E-11 |
| 544123 | PR1b1 | 7.536379887 | 1.38E-14 |
| 544133 | CrtR-b1 | 5.466197276 | 5.59E-15 |
| 544134 | TSI-1 | 7.576638427 | 9.52E-10 |
| 544146 | CHI14 | 4.775671762 | 2.26E-07 |
| 544148 | CHI9 | 4.431403525 | 3.11E-06 |
| 544149 | CHI3 | 6.175492723 | 4.97E-11 |
| 544154 | CS1 | 1.086122149 | 0.004248578 |
| 544164 | ME1 | 2.079887386 | 1.27E-04 |
| 544168 | ER24 | 4.169639077 | 2.59E-07 |
| 544180 | Abz1 | 2.724225179 | 0.002690642 |
| 544181 | PLDb1 | 1.598004071 | 0.00382328 |
| 544185 | P4 | 8.691768384 | 2.47E-06 |
| 544219 | CRY1a | 1.263859116 | 1.45E-04 |
| 544222 | DDTFR8 | 2.415959628 | 4.17E-04 |
| 544236 | SIP | 2.833724937 | 5.16E-05 |
| 544254 | THT1-3 | 4.555561059 | 4.56E-06 |
| 544258 | GABA-TP3 | 1.900755292 | 0.007407187 |
| 544264 | LOC544264 | 1.905986531 | 0.004516017 |
| 544267 | LOC544267 | 3.813397869 | 1.09E-08 |
| 544269 | CCD1B | 2.114206018 | 3.32E-07 |
| 544270 | NML2 | 1.938139408 | 1.83E-07 |
| 544282 | LOC544282 | 8.760913682 | 9.13E-12 |
| 544285 | EFE | 2.332029515 | 3.11E-04 |
| 544296 | P69B | 4.103093108 | 3.19E-08 |
| 544312 | LOC544312 | 3.084979657 | 5.36E-07 |
| 606304 | ACS2 | 5.114464583 | 2.63E-05 |
| 606712 | LOC606712 | 3.192956809 | 3.68E-05 |
| 778202 | PDH | 4.40453257 | 2.77E-04 |
| 778219 | LOC778219 | 6.810251036 | 0.001035534 |
| 778220 | ACI112 | 4.171674447 | 6.57E-13 |
| 778253 | CO1 | 6.755293239 | 8.03E-21 |
| 778260 | Sfr2 | 1.05807921 | 0.001318896 |
| 778264 | LOC778264 | 1.60429367 | 0.006527788 |
| 778273 | Pip1 | 3.126244478 | 1.68E-06 |
| 778293 | 9612 | 7.60184895 | 1.70E-09 |
| 778308 | LOC778308 | 6.627194992 | 5.63E-13 |
| 778310 | GGPS1 | 1.929920037 | 0.005755667 |
| 778321 | LOC778321 | 8.51164609 | 8.44E-23 |
| 778334 | CIP1 | 2.905515525 | 6.86E-10 |
| 778336 | ChrC | 1.852336176 | 4.34E-04 |
| 778337 | FtsH6 | 3.651134671 | 2.50E-05 |
| 778357 | LysRS | 5.78929903 | 0.002140906 |
| 778359 | CCR2 | 2.03111537 | 2.74E-04 |
| 778362 | LOC778362 | 2.083716217 | 4.28E-04 |

**Table S2. Differentially expressed genes downregulates by *Cladosporium fulvum* treatment**

| **Gene ID** | **Gene Symbol** | **Log_2_**  **(*C.fulvum*/ Control)** | ***P*-value**  **(*C.fulvum*/ Control)** |
| --- | --- | --- | --- |
| 100037497 | GRAS1 | -1.719871577 | 0.002330423 |
| 100037506 | MBF1a | -2.073928631 | 3.79E-06 |
| 100037513 | TCP3 | -4.467663786 | 9.62E-20 |
| 100134884 | Tm-1 | -1.000631272 | 0.002738519 |
| 100134915 | LOC100134915 | -3.427716985 | 1.63E-09 |
| 100147723 | GAD3 | -1.792425058 | 0.008195493 |
| 100147725 | SSR2 | -1.75176695 | 1.27E-04 |
| 100191121 | WRKY3 | -1.709281947 | 0.004666834 |
| 100191129 | LOC100191129 | -6.230770599 | 5.78E-12 |
| 100271890 | ZIP | -2.270081112 | 3.22E-04 |
| 100301919 | LOC100301919 | -2.560077719 | 2.58E-08 |
| 100301920 | LOC100301920 | -1.704385634 | 7.40E-04 |
| 100301923 | LOC100301923 | -2.197131805 | 0.008258262 |
| 100301924 | MDR1 | -1.774245328 | 7.73E-07 |
| 100301932 | UBC3 | -2.360870676 | 5.22E-13 |
| 100301933 | LOC100301933 | -3.124509202 | 7.77E-05 |
| 100301938 | SQD1 | -1.633891418 | 2.40E-05 |
| 100301942 | Style2.1 | -3.322433883 | 2.57E-07 |
| 100301945 | ARF8b | -1.045355577 | 0.008485145 |
| 100316881 | LOC100316881 | -1.13614383 | 4.58E-05 |
| 100316883 | PHS1 | -4.492110288 | 8.06E-06 |
| 100316884 | NDPS1 | -5.552622074 | 4.36E-07 |
| 100527965 | PIN1 | -2.423452466 | 1.01E-06 |
| 100527969 | PIN5 | -6.088216217 | 1.20E-05 |
| 100527970 | PIN6 | -3.482315771 | 0.001445023 |
| 100527971 | PIN7 | -2.347898148 | 9.87E-04 |
| 100736448 | ARF5 | -3.499673427 | 3.37E-11 |
| 100736460 | TCP23 | -1.864646026 | 0.003420033 |
| 100736461 | TCP22 | -2.608023072 | 5.16E-04 |
| 100736472 | AP2d | -2.314638462 | 1.84E-05 |
| 100736477 | LAX2 | -3.380373732 | 4.80E-14 |
| 100736482 | F3H | -2.402932548 | 5.11E-06 |
| 100736500 | LOC100736500 | -1.287074001 | 0.007910955 |
| 100736504 | F35H | -4.549575169 | 7.31E-11 |
| 100736511 | ARF10B | -2.343448659 | 4.72E-04 |
| 100736515 | TCP1 | -1.754462373 | 9.52E-05 |
| 100736521 | TCP17 | -1.654224396 | 1.98E-05 |
| 100736524 | TCP24 | -1.643119759 | 3.62E-09 |
| 100736528 | GAME1 | -2.189228916 | 4.02E-05 |
| 100736530 | SERK1 | -2.964773389 | 1.14E-19 |
| 100736535 | AP2e | -2.1960325 | 4.59E-07 |
| 100736541 | LAX5 | -4.552750611 | 6.05E-04 |
| 100736543 | LYK10 | -1.896437867 | 2.67E-04 |
| 100736546 | FtsZ1 | -1.538075013 | 4.81E-07 |
| 100820704 | ABF4 | -1.095517324 | 0.006243886 |
| 100874538 | LOG7 | -2.541794819 | 0.006669072 |
| 100874539 | LOG6 | -2.056431574 | 6.31E-04 |
| 101055507 | LOC101055507 | -2.489096585 | 3.90E-07 |
| 101055512 | LOC101055512 | -2.65654325 | 2.78E-07 |
| 101055513 | LOC101055513 | -3.377181969 | 1.82E-05 |
| 101055514 | LOC101055514 | -1.702710686 | 6.44E-05 |
| 101055515 | LOC101055515 | -1.540481268 | 0.005896414 |
| 101055518 | LOC101055518 | -1.744006035 | 3.73E-05 |
| 101055525 | LOC101055525 | -1.47637669 | 1.48E-04 |
| 101055528 | LOC101055528 | -2.766270612 | 0.001447849 |
| 101055532 | LOC101055532 | -1.536247983 | 0.001056027 |
| 101055537 | LOC101055537 | -1.732803356 | 0.006629972 |
| 101055548 | IAA15 | -1.830516887 | 0.002256359 |
| 101055549 | IAA19 | -1.919170305 | 0.004643909 |
| 101055559 | Arf/Xyl2 | -2.928318596 | 7.60E-05 |
| 101055604 | LOC101055604 | -1.629145582 | 1.46E-05 |
| 101055606 | LOC101055606 | -1.298908122 | 0.001032428 |
| 101243651 | LOC101243651 | -5.797558308 | 4.68E-05 |
| 101243671 | LOC101243671 | -1.323564624 | 0.007268058 |
| 101243676 | LOC101243676 | -2.692665021 | 4.44E-05 |
| 101243699 | LOC101243699 | -3.171193079 | 3.18E-07 |
| 101243748 | LOC101243748 | -3.860274886 | 6.70E-07 |
| 101243774 | LOC101243774 | -2.687212612 | 1.12E-08 |
| 101243777 | LOC101243777 | -2.933175081 | 7.75E-04 |
| 101243788 | LOC101243788 | -1.290205 | 0.004247955 |
| 101243790 | LOC101243790 | -2.78277629 | 9.59E-04 |
| 101243804 | LOC101243804 | -1.747013689 | 1.30E-04 |
| 101243813 | LOC101243813 | -2.070264939 | 0.004524373 |
| 101243818 | LOC101243818 | -1.984699008 | 6.58E-04 |
| 101243821 | LOC101243821 | -2.700292504 | 1.98E-06 |
| 101243827 | LOC101243827 | -1.896781287 | 5.72E-05 |
| 101243834 | LOC101243834 | -2.791816971 | 3.17E-07 |
| 101243849 | LOC101243849 | -1.563757829 | 0.001349632 |
| 101243853 | LOC101243853 | -2.327113706 | 0.008232857 |
| 101243868 | LOC101243868 | -2.286992924 | 0.001125582 |
| 101243905 | LOC101243905 | -1.59463228 | 6.64E-04 |
| 101243908 | LOC101243908 | -1.461586621 | 0.001143829 |
| 101243910 | LOC101243910 | -1.856207371 | 0.006204469 |
| 101243913 | LOC101243913 | -2.138964667 | 8.77E-05 |
| 101243933 | LOC101243933 | -1.444985668 | 0.006769224 |
| 101243971 | ABCG54 | -1.45978775 | 0.007357851 |
| 101243975 | LOC101243975 | -3.966323292 | 9.98E-19 |
| 101244013 | LOC101244013 | -1.69842798 | 2.34E-07 |
| 101244014 | LOC101244014 | -1.570690384 | 7.00E-04 |
| 101244059 | LOC101244059 | -1.967975903 | 0.002278457 |
| 101244085 | CRF6 | -2.399725607 | 4.52E-04 |
| 101244092 | LOC101244092 | -2.762016047 | 2.59E-06 |
| 101244112 | LOC101244112 | -2.872501349 | 1.55E-04 |
| 101244113 | LOC101244113 | -1.252213529 | 4.43E-04 |
| 101244126 | LOC101244126 | -2.02601516 | 0.001092822 |
| 101244133 | LOC101244133 | -4.855072189 | 1.44E-11 |
| 101244147 | LOC101244147 | -2.079740194 | 9.95E-04 |
| 101244155 | LOC101244155 | -1.916987704 | 1.43E-05 |
| 101244167 | LOC101244167 | -3.879732865 | 4.19E-14 |
| 101244192 | LOC101244192 | -1.753585931 | 0.00755986 |
| 101244194 | LOC101244194 | -2.343638895 | 3.62E-04 |
| 101244208 | LOC101244208 | -3.43989513 | 2.55E-06 |
| 101244223 | LOC101244223 | -1.782450652 | 0.008064307 |
| 101244228 | LOC101244228 | -2.250235764 | 5.78E-04 |
| 101244240 | LOC101244240 | -1.977182982 | 3.86E-05 |
| 101244266 | ABCG53 | -2.32331374 | 4.68E-06 |
| 101244286 | LOC101244286 | -2.331120851 | 0.003182856 |
| 101244298 | LOC101244298 | -2.28808516 | 2.02E-07 |
| 101244316 | LOC101244316 | -5.469584333 | 1.25E-11 |
| 101244326 | LOC101244326 | -1.386241028 | 2.99E-04 |
| 101244333 | LOC101244333 | -1.027233985 | 0.001598515 |
| 101244365 | LOC101244365 | -3.079878227 | 1.06E-07 |
| 101244370 | LOC101244370 | -1.708329532 | 1.77E-04 |
| 101244392 | LOC101244392 | -4.20030008 | 1.63E-11 |
| 101244412 | LOC101244412 | -4.596644069 | 4.77E-26 |
| 101244415 | LOC101244415 | -1.822517018 | 2.67E-06 |
| 101244429 | LOC101244429 | -6.714303572 | 8.78E-05 |
| 101244454 | LOC101244454 | -1.541370143 | 0.006402968 |
| 101244455 | LOC101244455 | -4.893070218 | 1.11E-04 |
| 101244458 | LOC101244458 | -1.477809329 | 2.23E-04 |
| 101244469 | LOC101244469 | -1.577813275 | 1.70E-04 |
| 101244475 | LOC101244475 | -1.43355921 | 0.005355108 |
| 101244503 | LOC101244503 | -2.212265248 | 2.55E-05 |
| 101244505 | LOC101244505 | -1.422533187 | 0.002253824 |
| 101244530 | LOC101244530 | -2.404106935 | 0.005748667 |
| 101244535 | LOC101244535 | -1.257812159 | 0.004262546 |
| 101244555 | LOC101244555 | -1.5826081 | 1.10E-06 |
| 101244564 | LOC101244564 | -3.088679342 | 2.76E-06 |
| 101244570 | LOC101244570 | -2.176181741 | 0.008794017 |
| 101244590 | LOC101244590 | -1.058114974 | 7.82E-04 |
| 101244593 | LOC101244593 | -2.242002431 | 0.001309264 |
| 101244597 | LOC101244597 | -3.572959575 | 9.74E-04 |
| 101244612 | LOC101244612 | -1.701850174 | 2.47E-05 |
| 101244632 | LOC101244632 | -1.598588028 | 0.005194634 |
| 101244640 | LOC101244640 | -3.950171363 | 6.22E-17 |
| 101244651 | LOC101244651 | -1.249514425 | 5.73E-05 |
| 101244658 | LOC101244658 | -1.207183495 | 0.003081338 |
| 101244659 | LOC101244659 | -4.970432532 | 0.001432668 |
| 101244695 | LOC101244695 | -2.041280883 | 5.08E-04 |
| 101244702 | LOC101244702 | -4.131695003 | 4.17E-12 |
| 101244706 | LOC101244706 | -1.360457536 | 0.007839258 |
| 101244708 | LOC101244708 | -1.28785469 | 9.90E-05 |
| 101244709 | LOC101244709 | -1.813602911 | 1.01E-06 |
| 101244717 | POR | -2.863653189 | 1.63E-05 |
| 101244726 | LOC101244726 | -2.21091706 | 8.93E-07 |
| 101244728 | LOC101244728 | -3.977694224 | 4.44E-06 |
| 101244747 | LOC101244747 | -6.751717139 | 1.62E-18 |
| 101244762 | LOC101244762 | -1.118981544 | 0.001086613 |
| 101244766 | LOC101244766 | -1.934590726 | 2.62E-05 |
| 101244780 | LOC101244780 | -1.453221342 | 0.003734088 |
| 101244786 | LOC101244786 | -2.309699394 | 7.53E-06 |
| 101244800 | LOC101244800 | -3.051129079 | 0.001029917 |
| 101244813 | LOC101244813 | -5.488143416 | 2.02E-14 |
| 101244833 | LOC101244833 | -2.092438367 | 1.53E-09 |
| 101244841 | LOC101244841 | -1.263697358 | 4.68E-04 |
| 101244864 | LOC101244864 | -2.037864376 | 2.51E-09 |
| 101244868 | LOC101244868 | -1.164045025 | 0.001495738 |
| 101244876 | LOC101244876 | -2.137636233 | 1.06E-10 |
| 101244879 | PCL1 | -2.522481693 | 0.006831099 |
| 101244887 | LOC101244887 | -2.968738431 | 2.46E-08 |
| 101244915 | LOC101244915 | -4.948183618 | 7.76E-14 |
| 101244916 | LOC101244916 | -1.202033431 | 0.006569195 |
| 101244926 | LOC101244926 | -2.684752868 | 4.91E-05 |
| 101244960 | LOC101244960 | -1.239909789 | 0.007306358 |
| 101244969 | LOC101244969 | -3.612924174 | 0.001194582 |
| 101244979 | LOC101244979 | -1.314860774 | 0.00460601 |
| 101244998 | LOC101244998 | -1.548057956 | 0.003275554 |
| 101245017 | LOC101245017 | -1.713124062 | 2.04E-04 |
| 101245044 | LOC101245044 | -2.8787951 | 2.81E-04 |
| 101245049 | LOC101245049 | -5.800493529 | 1.26E-23 |
| 101245111 | LOC101245111 | -2.191391567 | 0.003651241 |
| 101245116 | LOC101245116 | -1.809242177 | 1.85E-05 |
| 101245143 | LOC101245143 | -2.383966185 | 0.004598101 |
| 101245147 | LOC101245147 | -2.074658487 | 1.78E-06 |
| 101245153 | CYP736A1 | -3.587136761 | 3.11E-09 |
| 101245163 | LOC101245163 | -1.143824333 | 0.001435816 |
| 101245177 | LOC101245177 | -1.43346797 | 0.00106294 |
| 101245189 | LOC101245189 | -1.516878892 | 0.007450541 |
| 101245207 | LOC101245207 | -5.54336048 | 8.12E-10 |
| 101245251 | LOC101245251 | -6.261129427 | 5.37E-09 |
| 101245252 | LOC101245252 | -1.162983875 | 0.007450763 |
| 101245253 | LOC101245253 | -2.145823298 | 3.56E-04 |
| 101245262 | LOC101245262 | -1.129352727 | 0.00384598 |
| 101245263 | LOC101245263 | -3.335526491 | 2.11E-08 |
| 101245265 | LOC101245265 | -3.185447263 | 2.51E-04 |
| 101245272 | LOC101245272 | -2.414350121 | 9.23E-07 |
| 101245279 | LOC101245279 | -1.189226369 | 0.008206614 |
| 101245314 | LOC101245314 | -2.740297542 | 4.65E-06 |
| 101245315 | LOC101245315 | -1.472885613 | 1.19E-04 |
| 101245334 | LOC101245334 | -3.912089201 | 1.33E-06 |
| 101245335 | LOC101245335 | -1.845711038 | 8.73E-06 |
| 101245350 | LOC101245350 | -1.943896889 | 0.003149933 |
| 101245371 | LOC101245371 | -3.381349665 | 5.94E-06 |
| 101245408 | LOC101245408 | -1.63084031 | 1.48E-04 |
| 101245429 | LOC101245429 | -1.956893921 | 0.002151173 |
| 101245455 | LOC101245455 | -1.282844245 | 0.004471098 |
| 101245495 | LOC101245495 | -2.675179579 | 4.18E-04 |
| 101245496 | LOC101245496 | -1.52254939 | 0.009291191 |
| 101245497 | LOC101245497 | -1.947051044 | 2.32E-05 |
| 101245505 | LOC101245505 | -2.670994251 | 4.01E-10 |
| 101245517 | LOC101245517 | -5.754313592 | 8.03E-53 |
| 101245554 | LOC101245554 | -1.959259275 | 4.61E-04 |
| 101245555 | LOC101245555 | -2.736628732 | 1.47E-12 |
| 101245569 | LOC101245569 | -3.920579355 | 2.53E-04 |
| 101245573 | NAOAT | -1.119569389 | 2.90E-06 |
| 101245575 | LOC101245575 | -1.332648759 | 6.76E-04 |
| 101245600 | LOC101245600 | -2.013301835 | 2.30E-04 |
| 101245602 | LOC101245602 | -2.903275143 | 2.23E-04 |
| 101245618 | LOC101245618 | -1.62430792 | 0.005690752 |
| 101245628 | LOC101245628 | -1.114856527 | 0.003173419 |
| 101245662 | LOC101245662 | -2.489698476 | 2.35E-10 |
| 101245664 | LOC101245664 | -2.654359801 | 1.03E-04 |
| 101245680 | LOC101245680 | -1.382897178 | 0.008748801 |
| 101245692 | LOC101245692 | -1.56468767 | 0.00128286 |
| 101245724 | LOC101245724 | -2.730472199 | 0.001709984 |
| 101245741 | LOC101245741 | -1.497283233 | 2.48E-06 |
| 101245743 | LOC101245743 | -2.608059869 | 0.002619374 |
| 101245748 | LOC101245748 | -2.720597577 | 0.001481405 |
| 101245785 | LOC101245785 | -1.281242387 | 3.43E-06 |
| 101245790 | LOC101245790 | -2.034798917 | 6.13E-08 |
| 101245793 | LOC101245793 | -3.202106207 | 6.11E-05 |
| 101245805 | LOC101245805 | -2.64346265 | 2.40E-05 |
| 101245814 | LOC101245814 | -1.378821489 | 0.003220931 |
| 101245858 | LOC101245858 | -2.328972616 | 1.84E-10 |
| 101245901 | LOC101245901 | -1.053114394 | 3.09E-04 |
| 101245909 | LOC101245909 | -1.222348857 | 0.004371009 |
| 101245913 | LOC101245913 | -1.375023416 | 0.003202271 |
| 101245927 | LOC101245927 | -1.232260408 | 1.59E-04 |
| 101245933 | LOC101245933 | -2.418600088 | 1.09E-06 |
| 101245965 | LOC101245965 | -6.864902685 | 1.00E-06 |
| 101245996 | LOC101245996 | -1.335519899 | 0.002263674 |
| 101245999 | LOC101245999 | -4.069535002 | 4.69E-09 |
| 101246001 | BOP2 | -3.212511035 | 0.006220984 |
| 101246013 | LOC101246013 | -1.824254126 | 4.73E-10 |
| 101246021 | LOC101246021 | -1.355543062 | 0.006258217 |
| 101246034 | LOC101246034 | -1.529032249 | 1.27E-04 |
| 101246039 | LOC101246039 | -1.514301645 | 0.004936411 |
| 101246050 | LOC101246050 | -2.153173423 | 8.91E-06 |
| 101246051 | LOC101246051 | -1.455723869 | 0.004458667 |
| 101246055 | LOC101246055 | -1.305593103 | 3.09E-04 |
| 101246062 | LOC101246062 | -1.30659592 | 2.12E-04 |
| 101246107 | LOC101246107 | -1.04468093 | 0.002830289 |
| 101246125 | LOC101246125 | -2.94429741 | 1.76E-06 |
| 101246132 | LOC101246132 | -1.116880087 | 0.00228261 |
| 101246133 | LOC101246133 | -2.36759171 | 1.23E-08 |
| 101246185 | LOC101246185 | -5.298813412 | 0.005425549 |
| 101246207 | LOC101246207 | -2.287711855 | 1.03E-05 |
| 101246223 | LOC101246223 | -6.324745261 | 2.28E-05 |
| 101246265 | LOC101246265 | -2.41075924 | 0.001005736 |
| 101246270 | LOC101246270 | -2.093825088 | 6.36E-04 |
| 101246303 | LOC101246303 | -1.390053083 | 3.98E-06 |
| 101246319 | LOC101246319 | -1.25766047 | 0.001143625 |
| 101246361 | LOC101246361 | -1.584885686 | 3.80E-04 |
| 101246365 | ISPF | -1.713065673 | 2.17E-04 |
| 101246371 | GGPS | -1.124131877 | 8.55E-05 |
| 101246394 | LOC101246394 | -3.059588236 | 1.41E-08 |
| 101246403 | LOC101246403 | -1.248537336 | 0.001997022 |
| 101246415 | LOC101246415 | -1.500227312 | 0.001131462 |
| 101246418 | LOC101246418 | -1.731979609 | 0.006994894 |
| 101246423 | LOC101246423 | -2.80454529 | 1.09E-04 |
| 101246434 | LOC101246434 | -2.496650413 | 3.15E-04 |
| 101246467 | LOC101246467 | -1.689547831 | 0.001234294 |
| 101246535 | LOC101246535 | -1.66199224 | 3.75E-05 |
| 101246541 | LOC101246541 | -1.373534394 | 0.004703758 |
| 101246603 | LOC101246603 | -4.121802593 | 1.51E-05 |
| 101246610 | LOC101246610 | -2.23494189 | 0.002326031 |
| 101246642 | LOC101246642 | -2.574463238 | 4.65E-04 |
| 101246712 | LOC101246712 | -1.669246523 | 3.05E-04 |
| 101246723 | LOC101246723 | -1.089007623 | 3.99E-04 |
| 101246730 | LOC101246730 | -1.889579893 | 1.50E-08 |
| 101246732 | LOC101246732 | -2.234720293 | 0.001401844 |
| 101246754 | LOC101246754 | -1.480717069 | 5.95E-04 |
| 101246762 | LOC101246762 | -1.76781964 | 0.00301442 |
| 101246771 | LOC101246771 | -1.824787233 | 5.44E-05 |
| 101246778 | LOC101246778 | -2.783848173 | 1.53E-04 |
| 101246802 | LOC101246802 | -1.309833737 | 4.34E-05 |
| 101246815 | LOC101246815 | -1.470487917 | 0.001693163 |
| 101246829 | LOC101246829 | -4.691258626 | 2.83E-15 |
| 101246864 | LOC101246864 | -2.898341285 | 6.23E-06 |
| 101246877 | LOC101246877 | -2.24911461 | 1.16E-08 |
| 101246884 | LOC101246884 | -5.832827032 | 4.99E-25 |
| 101246937 | LOC101246937 | -2.789231428 | 8.16E-06 |
| 101246952 | LOC101246952 | -1.319698187 | 0.001101299 |
| 101246986 | LOC101246986 | -2.230907676 | 3.78E-08 |
| 101246993 | LOC101246993 | -3.452475985 | 1.52E-05 |
| 101246996 | LOC101246996 | -2.48540482 | 8.98E-10 |
| 101247008 | LOC101247008 | -3.583173081 | 3.00E-06 |
| 101247043 | LOC101247043 | -2.662614053 | 0.002246352 |
| 101247045 | LOC101247045 | -2.31838609 | 0.003155957 |
| 101247048 | LOC101247048 | -3.276555047 | 4.83E-04 |
| 101247050 | LOC101247050 | -2.954067832 | 0.003121259 |
| 101247077 | LOC101247077 | -1.414459178 | 9.42E-04 |
| 101247080 | LOC101247080 | -2.908034189 | 0.005194997 |
| 101247088 | LOC101247088 | -1.708607598 | 0.002133111 |
| 101247110 | LOC101247110 | -3.515536698 | 0.001592528 |
| 101247128 | LOC101247128 | -3.374861303 | 1.58E-10 |
| 101247137 | LOC101247137 | -2.09868263 | 2.10E-04 |
| 101247177 | LOC101247177 | -1.945146073 | 4.40E-04 |
| 101247196 | LOC101247196 | -2.285421323 | 6.31E-04 |
| 101247216 | LOC101247216 | -3.667993353 | 0.003744774 |
| 101247223 | LOC101247223 | -1.475506241 | 4.67E-04 |
| 101247232 | LOC101247232 | -7.082880286 | 3.83E-18 |
| 101247242 | LOC101247242 | -2.748440238 | 1.38E-04 |
| 101247249 | LOC101247249 | -1.715789922 | 0.002007012 |
| 101247257 | LOC101247257 | -4.911352411 | 1.52E-04 |
| 101247267 | LOC101247267 | -1.02502705 | 0.004146694 |
| 101247271 | LOC101247271 | -1.653867193 | 0.004021071 |
| 101247272 | LOC101247272 | -1.931449411 | 0.002356147 |
| 101247280 | LOC101247280 | -2.948714051 | 2.37E-10 |
| 101247284 | LOC101247284 | -2.81046201 | 2.62E-08 |
| 101247286 | LOC101247286 | -1.373621518 | 0.001014193 |
| 101247292 | LOC101247292 | -1.484294621 | 2.66E-05 |
| 101247302 | LOC101247302 | -1.291337715 | 0.001352809 |
| 101247315 | LOC101247315 | -4.120765438 | 1.67E-07 |
| 101247367 | LOC101247367 | -2.007373352 | 0.002900818 |
| 101247374 | LOC101247374 | -1.446513191 | 0.00181597 |
| 101247399 | LOC101247399 | -2.927331918 | 1.24E-04 |
| 101247446 | LOC101247446 | -4.78620601 | 1.85E-12 |
| 101247490 | LOC101247490 | -2.045623865 | 1.23E-04 |
| 101247496 | LOC101247496 | -3.613828816 | 6.62E-11 |
| 101247504 | LOC101247504 | -1.754740855 | 0.004004901 |
| 101247513 | LOC101247513 | -4.082296736 | 3.25E-17 |
| 101247526 | LOC101247526 | -1.471962633 | 1.58E-04 |
| 101247532 | LOC101247532 | -1.230345462 | 0.006414672 |
| 101247539 | LOC101247539 | -5.967974042 | 1.90E-04 |
| 101247546 | LOC101247546 | -1.266578794 | 0.005978403 |
| 101247604 | LOC101247604 | -2.736894766 | 5.15E-04 |
| 101247638 | LOC101247638 | -1.693868043 | 0.001728442 |
| 101247639 | LOC101247639 | -2.810900804 | 1.59E-04 |
| 101247640 | LOC101247640 | -1.785466769 | 0.003689179 |
| 101247645 | LOC101247645 | -1.660303435 | 0.004137085 |
| 101247653 | LOC101247653 | -2.103817274 | 5.58E-04 |
| 101247655 | LOC101247655 | -2.754101124 | 5.25E-06 |
| 101247661 | LOC101247661 | -3.035672622 | 5.49E-06 |
| 101247670 | LOC101247670 | -3.041724248 | 6.99E-10 |
| 101247684 | LOC101247684 | -1.476461386 | 9.35E-05 |
| 101247693 | LOC101247693 | -2.625737097 | 2.62E-05 |
| 101247699 | LOC101247699 | -1.226272655 | 0.008856172 |
| 101247714 | LOC101247714 | -2.418282092 | 1.60E-04 |
| 101247737 | LOC101247737 | -6.598937128 | 7.77E-05 |
| 101247741 | LOC101247741 | -1.404433719 | 2.75E-04 |
| 101247788 | LOC101247788 | -1.03741855 | 1.42E-04 |
| 101247820 | LOC101247820 | -1.789411049 | 0.003734374 |
| 101247833 | LOC101247833 | -2.992352638 | 1.93E-13 |
| 101247848 | LOC101247848 | -2.215284323 | 4.93E-04 |
| 101247855 | LOC101247855 | -2.108261973 | 3.34E-06 |
| 101247860 | LOC101247860 | -2.822796301 | 3.86E-05 |
| 101247865 | TPS7 | -1.888823734 | 7.21E-05 |
| 101247885 | LOC101247885 | -2.169315604 | 4.66E-07 |
| 101247921 | LOC101247921 | -2.687850165 | 2.13E-04 |
| 101247922 | LOC101247922 | -1.760314513 | 0.005972683 |
| 101247925 | LOC101247925 | -2.369525527 | 0.00193018 |
| 101247927 | LOC101247927 | -1.250733221 | 1.15E-04 |
| 101247932 | LOC101247932 | -3.436145428 | 8.59E-05 |
| 101247960 | LOC101247960 | -2.463921957 | 3.55E-05 |
| 101247978 | LOC101247978 | -1.674466894 | 2.06E-04 |
| 101247980 | LOC101247980 | -5.214369368 | 6.98E-23 |
| 101248000 | LOC101248000 | -1.878751252 | 0.003026164 |
| 101248013 | LOC101248013 | -4.529056067 | 1.62E-04 |
| 101248014 | LOC101248014 | -1.733374542 | 0.004973541 |
| 101248016 | LOC101248016 | -5.505933829 | 0.001227209 |
| 101248033 | LOC101248033 | -1.02047488 | 0.009138601 |
| 101248047 | LOC101248047 | -1.391100608 | 1.18E-04 |
| 101248065 | LOC101248065 | -5.445983005 | 0.002862421 |
| 101248071 | LOC101248071 | -1.099690673 | 0.004418179 |
| 101248078 | LOC101248078 | -1.650216984 | 1.32E-04 |
| 101248079 | LOC101248079 | -2.958453105 | 8.80E-04 |
| 101248086 | LOC101248086 | -1.449134751 | 0.003289084 |
| 101248093 | LOC101248093 | -1.629942084 | 9.30E-05 |
| 101248098 | LOC101248098 | -1.38102681 | 0.002893236 |
| 101248135 | LOC101248135 | -3.150307913 | 6.24E-05 |
| 101248202 | LOC101248202 | -3.195475367 | 0.002256067 |
| 101248209 | LOC101248209 | -3.280189706 | 0.004654411 |
| 101248216 | LOC101248216 | -2.569258158 | 9.19E-05 |
| 101248254 | LOC101248254 | -3.554241538 | 8.18E-08 |
| 101248260 | LOC101248260 | -1.00966971 | 0.003071007 |
| 101248277 | LOC101248277 | -2.495128748 | 4.50E-05 |
| 101248295 | LOC101248295 | -3.432809654 | 5.52E-26 |
| 101248318 | LOC101248318 | -4.828740152 | 3.54E-05 |
| 101248337 | LOC101248337 | -3.100015604 | 2.31E-09 |
| 101248358 | LOC101248358 | -1.455825745 | 0.0072071 |
| 101248362 | LOC101248362 | -1.096113573 | 0.002624058 |
| 101248367 | LOC101248367 | -7.402974839 | 3.00E-13 |
| 101248370 | LOC101248370 | -1.928794268 | 0.002765232 |
| 101248375 | LOC101248375 | -1.947979441 | 0.002638586 |
| 101248387 | LOC101248387 | -2.892050815 | 2.34E-05 |
| 101248444 | LOC101248444 | -2.503324066 | 0.003269741 |
| 101248448 | LOC101248448 | -1.389469697 | 0.001750613 |
| 101248451 | LOC101248451 | -2.440806537 | 0.007738565 |
| 101248488 | LOC101248488 | -2.392398246 | 0.008441733 |
| 101248495 | LOC101248495 | -3.471430851 | 2.91E-09 |
| 101248518 | LOC101248518 | -1.225656925 | 5.19E-04 |
| 101248535 | LOC101248535 | -1.634217446 | 3.46E-05 |
| 101248537 | LOC101248537 | -3.356920204 | 0.001087101 |
| 101248539 | LOC101248539 | -1.237013976 | 0.005393558 |
| 101248557 | LOC101248557 | -7.24661659 | 4.06E-13 |
| 101248567 | ABCA3 | -4.694325753 | 7.64E-10 |
| 101248582 | LOC101248582 | -6.038236845 | 0.001473115 |
| 101248599 | LOC101248599 | -4.442364876 | 9.89E-04 |
| 101248610 | LOC101248610 | -2.824728219 | 3.49E-05 |
| 101248630 | LOC101248630 | -2.487234003 | 3.45E-08 |
| 101248633 | LOC101248633 | -2.073450844 | 3.68E-04 |
| 101248645 | LOC101248645 | -1.777171067 | 4.69E-04 |
| 101248655 | LOC101248655 | -4.649921958 | 2.48E-12 |
| 101248671 | LOC101248671 | -1.541832678 | 1.00E-04 |
| 101248693 | LOC101248693 | -5.693382533 | 2.17E-63 |
| 101248703 | LOC101248703 | -6.903509858 | 3.67E-06 |
| 101248727 | LOC101248727 | -4.769640456 | 0.004494083 |
| 101248729 | LOC101248729 | -2.168788604 | 0.003331818 |
| 101248732 | LOC101248732 | -1.700389106 | 1.33E-06 |
| 101248771 | LOC101248771 | -1.183225532 | 0.002722647 |
| 101248773 | LOC101248773 | -4.698745263 | 4.49E-20 |
| 101248811 | LOC101248811 | -1.31937549 | 0.001040086 |
| 101248844 | LOC101248844 | -5.52533476 | 8.48E-04 |
| 101248851 | LOC101248851 | -2.913536384 | 0.003420093 |
| 101248854 | LOC101248854 | -3.154235443 | 4.17E-04 |
| 101248892 | LOC101248892 | -2.526102016 | 3.89E-05 |
| 101248895 | LOC101248895 | -2.554446221 | 1.11E-07 |
| 101248913 | LOC101248913 | -1.912970263 | 1.18E-04 |
| 101248919 | LOC101248919 | -4.899826603 | 2.68E-13 |
| 101248926 | CYCB1-2 | -3.483920355 | 5.87E-05 |
| 101248946 | LOC101248946 | -3.207994785 | 1.97E-04 |
| 101248960 | LOC101248960 | -1.720405497 | 8.71E-05 |
| 101248971 | LOC101248971 | -3.427378344 | 2.18E-08 |
| 101248985 | LOC101248985 | -1.912644 | 0.001176699 |
| 101249010 | LOC101249010 | -2.040132817 | 0.001170387 |
| 101249032 | LOC101249032 | -3.485648572 | 8.88E-04 |
| 101249056 | LOC101249056 | -2.15394075 | 5.56E-06 |
| 101249065 | LOC101249065 | -2.623250782 | 9.12E-04 |
| 101249066 | LOC101249066 | -2.430665968 | 0.007095815 |
| 101249076 | LOC101249076 | -1.482623738 | 6.17E-05 |
| 101249103 | LOC101249103 | -1.214561879 | 0.003416303 |
| 101249106 | LOC101249106 | -1.802054435 | 5.63E-05 |
| 101249113 | LOC101249113 | -1.231194971 | 3.48E-04 |
| 101249114 | LOC101249114 | -2.746603277 | 6.36E-04 |
| 101249132 | LOC101249132 | -2.684861381 | 3.13E-11 |
| 101249135 | LOC101249135 | -2.532469234 | 7.61E-07 |
| 101249148 | LOC101249148 | -3.962667166 | 0.001955904 |
| 101249169 | LOC101249169 | -2.168208716 | 5.26E-07 |
| 101249174 | LOC101249174 | -1.67818655 | 0.004007375 |
| 101249178 | LOC101249178 | -1.135596441 | 0.001951095 |
| 101249184 | LOC101249184 | -1.997625095 | 6.09E-05 |
| 101249193 | LOC101249193 | -1.995346397 | 8.44E-06 |
| 101249194 | LOC101249194 | -2.267402938 | 0.003236989 |
| 101249210 | LOC101249210 | -4.230075433 | 3.03E-05 |
| 101249224 | LOC101249224 | -1.372446497 | 1.83E-06 |
| 101249245 | LOC101249245 | -3.955589898 | 2.93E-11 |
| 101249247 | LOC101249247 | -1.369383454 | 0.001949037 |
| 101249258 | LOC101249258 | -2.354144807 | 8.30E-08 |
| 101249265 | CHI1 | -3.056765479 | 2.63E-05 |
| 101249277 | LOC101249277 | -1.869416252 | 0.00461966 |
| 101249297 | LOC101249297 | -2.560212825 | 5.38E-06 |
| 101249307 | LOC101249307 | -1.278935632 | 0.008971337 |
| 101249324 | LOC101249324 | -1.099084644 | 5.67E-04 |
| 101249329 | LOC101249329 | -3.177568553 | 1.90E-06 |
| 101249348 | LOC101249348 | -3.056486361 | 3.19E-05 |
| 101249352 | LOC101249352 | -1.074427623 | 0.00201019 |
| 101249357 | LOC101249357 | -1.760431558 | 4.84E-07 |
| 101249360 | LOC101249360 | -1.892481474 | 0.001196359 |
| 101249390 | LOC101249390 | -1.27820414 | 1.50E-04 |
| 101249396 | LOC101249396 | -3.341947259 | 1.35E-12 |
| 101249415 | LOC101249415 | -3.436893211 | 1.39E-07 |
| 101249457 | LOC101249457 | -2.029308076 | 7.72E-04 |
| 101249465 | LOC101249465 | -2.264262553 | 0.002479101 |
| 101249482 | LOC101249482 | -2.334499205 | 0.005144185 |
| 101249502 | LOC101249502 | -2.886088303 | 0.004127867 |
| 101249505 | LOC101249505 | -1.929060155 | 4.20E-06 |
| 101249506 | LOC101249506 | -1.689080859 | 1.44E-04 |
| 101249532 | LOC101249532 | -2.164257031 | 5.54E-05 |
| 101249535 | LOC101249535 | -6.143847265 | 4.05E-07 |
| 101249542 | LOC101249542 | -3.767749269 | 4.46E-15 |
| 101249543 | LOC101249543 | -2.07531831 | 3.37E-05 |
| 101249548 | LOC101249548 | -3.728968779 | 6.18E-05 |
| 101249557 | LOC101249557 | -2.66623924 | 2.94E-04 |
| 101249574 | LOC101249574 | -1.603700967 | 0.007160282 |
| 101249584 | LOC101249584 | -2.240869099 | 2.64E-10 |
| 101249597 | LOC101249597 | -2.106624604 | 6.04E-05 |
| 101249602 | LOC101249602 | -1.22582725 | 9.67E-04 |
| 101249605 | ORRM4 | -1.377573892 | 1.28E-04 |
| 101249609 | LOC101249609 | -6.850023785 | 1.68E-10 |
| 101249627 | LOC101249627 | -2.541537237 | 3.19E-06 |
| 101249681 | LOC101249681 | -1.758923473 | 9.12E-06 |
| 101249688 | LOC101249688 | -3.45082944 | 2.79E-05 |
| 101249698 | LOC101249698 | -1.4557077 | 0.00124652 |
| 101249701 | LOC101249701 | -1.524449628 | 0.002564965 |
| 101249727 | LOC101249727 | -2.265704695 | 1.10E-04 |
| 101249732 | LOC101249732 | -2.13306069 | 0.001814099 |
| 101249737 | LOC101249737 | -1.195864913 | 0.003385675 |
| 101249741 | LOC101249741 | -2.648155634 | 6.62E-05 |
| 101249770 | LOC101249770 | -2.162235709 | 2.28E-07 |
| 101249794 | LOC101249794 | -2.187652519 | 0.004197722 |
| 101249814 | LOC101249814 | -4.790475656 | 3.15E-21 |
| 101249840 | LOC101249840 | -1.87124814 | 2.54E-04 |
| 101249849 | LOC101249849 | -1.655761363 | 0.003478377 |
| 101249877 | LOC101249877 | -1.6965347 | 0.001165902 |
| 101249884 | LOC101249884 | -1.846667859 | 0.007839995 |
| 101249886 | LOC101249886 | -2.078294111 | 2.21E-04 |
| 101249913 | LOC101249913 | -1.840487912 | 5.28E-04 |
| 101249938 | LOC101249938 | -2.352874244 | 0.005110218 |
| 101249944 | LOC101249944 | -3.6423553 | 6.32E-25 |
| 101249980 | LOC101249980 | -2.041672531 | 1.90E-04 |
| 101249996 | LOC101249996 | -1.535173062 | 5.87E-06 |
| 101250006 | LOC101250006 | -6.042970412 | 0.001069566 |
| 101250010 | LOC101250010 | -1.196378822 | 3.28E-04 |
| 101250021 | LOC101250021 | -3.059854623 | 3.63E-05 |
| 101250049 | LOC101250049 | -2.086481473 | 8.63E-04 |
| 101250068 | LOC101250068 | -3.104190581 | 5.08E-05 |
| 101250072 | LOC101250072 | -3.242254975 | 1.37E-05 |
| 101250087 | LOC101250087 | -2.694956091 | 7.21E-06 |
| 101250110 | LOC101250110 | -2.560118097 | 1.67E-06 |
| 101250138 | LOC101250138 | -5.857475897 | 0.003017672 |
| 101250163 | HTA6 | -2.236785049 | 2.49E-04 |
| 101250180 | LOC101250180 | -2.452580971 | 7.87E-05 |
| 101250184 | LOC101250184 | -1.86486111 | 4.14E-05 |
| 101250197 | Cdc20-1 | -2.552373728 | 8.66E-04 |
| 101250277 | LOC101250277 | -1.355406061 | 7.98E-04 |
| 101250283 | LOC101250283 | -5.740654322 | 5.52E-30 |
| 101250315 | LOC101250315 | -2.849579248 | 0.001382517 |
| 101250330 | LOC101250330 | -1.531694576 | 0.005061784 |
| 101250346 | LOC101250346 | -1.934708786 | 4.85E-08 |
| 101250347 | LOC101250347 | -6.98150401 | 5.28E-06 |
| 101250350 | LOC101250350 | -2.447521606 | 0.003464806 |
| 101250352 | LOC101250352 | -3.200316443 | 3.65E-07 |
| 101250356 | LOC101250356 | -5.444732763 | 0.003253117 |
| 101250373 | LOC101250373 | -1.255122958 | 0.003277447 |
| 101250390 | LOC101250390 | -1.756678784 | 2.04E-09 |
| 101250399 | LOC101250399 | -1.785969619 | 0.003658642 |
| 101250403 | LOC101250403 | -1.419643089 | 8.83E-05 |
| 101250405 | LOC101250405 | -6.717158743 | 1.50E-53 |
| 101250416 | LOC101250416 | -4.297467832 | 6.48E-04 |
| 101250418 | LOC101250418 | -1.811004595 | 0.006829089 |
| 101250428 | LOC101250428 | -2.330046027 | 9.22E-04 |
| 101250432 | LOC101250432 | -1.809541751 | 0.001877509 |
| 101250437 | LOC101250437 | -1.660349177 | 0.002862865 |
| 101250447 | LOC101250447 | -2.448284508 | 0.005300978 |
| 101250457 | LOC101250457 | -1.221667211 | 0.001734304 |
| 101250460 | LOC101250460 | -2.641199121 | 5.97E-06 |
| 101250468 | LOC101250468 | -2.399773773 | 2.59E-04 |
| 101250473 | LOC101250473 | -3.090398228 | 7.21E-15 |
| 101250476 | LOC101250476 | -1.294619763 | 3.40E-05 |
| 101250517 | LOC101250517 | -5.01452351 | 2.73E-05 |
| 101250523 | LOC101250523 | -6.385724547 | 1.84E-05 |
| 101250561 | LOC101250561 | -2.183232136 | 5.55E-04 |
| 101250563 | LOC101250563 | -2.058685105 | 0.00135576 |
| 101250582 | LOC101250582 | -2.946714991 | 3.15E-07 |
| 101250611 | MKS1e | -2.68433599 | 0.004896825 |
| 101250615 | LOC101250615 | -2.568535656 | 0.001675491 |
| 101250621 | LOC101250621 | -2.53742479 | 5.87E-04 |
| 101250666 | LOC101250666 | -3.87288612 | 0.003352707 |
| 101250679 | LOC101250679 | -4.01010169 | 1.60E-15 |
| 101250680 | LOC101250680 | -2.232527049 | 7.01E-04 |
| 101250702 | LOC101250702 | -1.33864559 | 0.008982756 |
| 101250703 | LOC101250703 | -1.664716818 | 0.001433853 |
| 101250715 | LOC101250715 | -4.144435802 | 1.16E-14 |
| 101250719 | LOC101250719 | -5.473053893 | 2.46E-25 |
| 101250733 | LOC101250733 | -3.196700929 | 2.32E-04 |
| 101250736 | LOC101250736 | -4.539647124 | 1.47E-13 |
| 101250751 | LOC101250751 | -3.078692463 | 4.46E-04 |
| 101250753 | LOC101250753 | -2.294235234 | 9.31E-04 |
| 101250769 | LOC101250769 | -2.276522201 | 0.002850605 |
| 101250773 | LOC101250773 | -5.17686912 | 1.51E-19 |
| 101250784 | LOC101250784 | -1.360620344 | 0.002145872 |
| 101250787 | LOC101250787 | -2.710160272 | 1.36E-07 |
| 101250791 | LOC101250791 | -3.262600435 | 6.67E-04 |
| 101250812 | LOC101250812 | -1.484191346 | 4.13E-04 |
| 101250825 | LOC101250825 | -2.119828541 | 0.002178484 |
| 101250843 | LOC101250843 | -2.628281914 | 3.81E-06 |
| 101250852 | LOC101250852 | -3.154940945 | 4.25E-04 |
| 101250896 | LOC101250896 | -1.001580967 | 0.00712556 |
| 101250897 | LOC101250897 | -2.268756696 | 1.97E-06 |
| 101250905 | MKS1d | -2.432235099 | 0.002169071 |
| 101250910 | LOC101250910 | -1.631281706 | 3.01E-07 |
| 101250917 | LOC101250917 | -1.569904265 | 0.003627406 |
| 101250923 | LOC101250923 | -4.796518915 | 2.78E-07 |
| 101250929 | LOC101250929 | -2.914220224 | 2.82E-04 |
| 101250937 | LOC101250937 | -3.476483121 | 1.71E-04 |
| 101250943 | LOC101250943 | -1.847597466 | 0.001288476 |
| 101250946 | LOC101250946 | -1.442408687 | 0.001105339 |
| 101250963 | LOC101250963 | -1.520392064 | 0.008713505 |
| 101250983 | LOC101250983 | -1.895689201 | 4.35E-05 |
| 101250985 | LOC101250985 | -2.982251689 | 4.88E-05 |
| 101250995 | LOC101250995 | -1.531152131 | 0.00377681 |
| 101251011 | LOC101251011 | -7.473405657 | 2.03E-12 |
| 101251012 | LOC101251012 | -1.98605387 | 7.12E-14 |
| 101251021 | LOC101251021 | -2.618887668 | 1.31E-07 |
| 101251027 | LOC101251027 | -2.311046445 | 0.005163068 |
| 101251033 | LOC101251033 | -1.694414578 | 0.00406507 |
| 101251043 | LOC101251043 | -2.415278489 | 0.008135954 |
| 101251064 | LOC101251064 | -1.54304867 | 0.006205876 |
| 101251075 | FKBP17-3 | -1.95383841 | 2.30E-05 |
| 101251077 | LOC101251077 | -3.841523034 | 3.76E-05 |
| 101251103 | LOC101251103 | -2.095647306 | 5.67E-06 |
| 101251119 | JMJ524 | -5.367243531 | 1.01E-14 |
| 101251148 | LOC101251148 | -3.231471807 | 4.93E-04 |
| 101251174 | LOC101251174 | -1.837939069 | 0.002068835 |
| 101251179 | LOC101251179 | -2.260633752 | 6.45E-06 |
| 101251195 | LOC101251195 | -1.308469336 | 0.009139886 |
| 101251209 | LOC101251209 | -4.599268462 | 2.44E-10 |
| 101251221 | LOC101251221 | -2.664304046 | 5.99E-04 |
| 101251225 | LOC101251225 | -1.225650987 | 0.001249236 |
| 101251231 | LOC101251231 | -2.537766605 | 4.11E-04 |
| 101251240 | LOC101251240 | -6.980749931 | 3.23E-11 |
| 101251248 | LOC101251248 | -3.014740999 | 5.20E-04 |
| 101251249 | LOC101251249 | -1.916201365 | 3.08E-05 |
| 101251260 | LOC101251260 | -1.75557439 | 0.006284064 |
| 101251266 | LOC101251266 | -1.646897082 | 2.16E-05 |
| 101251285 | LOC101251285 | -2.004512225 | 0.001175341 |
| 101251297 | LOC101251297 | -3.018781998 | 8.40E-04 |
| 101251301 | LOC101251301 | -1.161599689 | 0.005002356 |
| 101251307 | LOC101251307 | -3.633752479 | 3.96E-05 |
| 101251308 | LOC101251308 | -1.770948292 | 0.003254428 |
| 101251384 | LOC101251384 | -2.87048179 | 4.76E-04 |
| 101251428 | LOC101251428 | -1.417604169 | 0.001464916 |
| 101251432 | LOC101251432 | -2.839902752 | 3.23E-12 |
| 101251433 | LOC101251433 | -1.376937451 | 0.00109901 |
| 101251442 | LOC101251442 | -1.9050956 | 9.26E-06 |
| 101251454 | LOC101251454 | -1.2398518 | 0.002772855 |
| 101251460 | LOC101251460 | -1.757384033 | 0.007274594 |
| 101251462 | LOC101251462 | -1.537782678 | 0.004591353 |
| 101251468 | LOC101251468 | -1.663954056 | 0.002234826 |
| 101251493 | LOC101251493 | -3.216415376 | 1.27E-04 |
| 101251513 | LOC101251513 | -1.409796472 | 0.002004337 |
| 101251531 | LOC101251531 | -1.46056086 | 0.004024958 |
| 101251532 | LOC101251532 | -1.491751684 | 0.001754546 |
| 101251542 | LOC101251542 | -1.459540495 | 2.30E-04 |
| 101251549 | LOC101251549 | -4.19946554 | 3.21E-04 |
| 101251550 | LOC101251550 | -1.975977774 | 1.83E-10 |
| 101251553 | LOC101251553 | -2.188570579 | 0.007228798 |
| 101251577 | LOC101251577 | -6.186338395 | 1.32E-15 |
| 101251582 | LOC101251582 | -1.215330228 | 0.00402015 |
| 101251596 | LOC101251596 | -3.004728328 | 1.16E-04 |
| 101251607 | ANS | -3.110819061 | 1.29E-07 |
| 101251616 | LOC101251616 | -1.4716326 | 8.55E-04 |
| 101251624 | LOC101251624 | -2.075871141 | 1.39E-06 |
| 101251636 | LOC101251636 | -2.219015787 | 0.006056513 |
| 101251642 | LOC101251642 | -2.0296855 | 2.80E-04 |
| 101251669 | LOC101251669 | -3.037645478 | 0.009132728 |
| 101251698 | LOC101251698 | -1.271009623 | 7.31E-05 |
| 101251748 | LOC101251748 | -6.568899666 | 3.44E-04 |
| 101251765 | LOC101251765 | -3.597178848 | 3.03E-07 |
| 101251767 | LOC101251767 | -1.187510152 | 0.008165236 |
| 101251772 | ANT | -2.399629892 | 6.27E-08 |
| 101251775 | AROGP3 | -4.066978131 | 1.93E-08 |
| 101251798 | LOC101251798 | -1.152673715 | 0.002106815 |
| 101251838 | LOC101251838 | -1.352949899 | 0.003687589 |
| 101251846 | LOC101251846 | -1.27193046 | 0.006977438 |
| 101251847 | LOC101251847 | -3.19062618 | 1.02E-04 |
| 101251855 | LOC101251855 | -1.729453985 | 0.001805406 |
| 101251862 | LOC101251862 | -3.107034411 | 2.01E-12 |
| 101251878 | CYP92B7 | -3.115709901 | 0.00101675 |
| 101251883 | LOC101251883 | -1.94659452 | 0.002094993 |
| 101251907 | IPMS2 | -2.21112745 | 4.53E-09 |
| 101251921 | LOC101251921 | -2.049615647 | 7.37E-04 |
| 101251967 | LOC101251967 | -1.884608214 | 0.006922939 |
| 101251978 | LOC101251978 | -1.57209682 | 1.26E-04 |
| 101251995 | LOC101251995 | -2.207520667 | 9.99E-04 |
| 101252021 | LOC101252021 | -2.253278141 | 0.005014397 |
| 101252035 | LOC101252035 | -2.114834115 | 9.16E-04 |
| 101252066 | LOC101252066 | -6.610569893 | 4.91E-16 |
| 101252067 | LOC101252067 | -1.90406243 | 4.80E-06 |
| 101252080 | AROGP2 | -5.474764108 | 1.01E-08 |
| 101252082 | LOC101252082 | -3.805582283 | 2.24E-21 |
| 101252093 | LOC101252093 | -1.357798429 | 0.00185268 |
| 101252154 | LOC101252154 | -2.661898841 | 1.05E-20 |
| 101252165 | LOC101252165 | -1.434250666 | 1.06E-06 |
| 101252173 | LOC101252173 | -4.785172286 | 8.51E-07 |
| 101252186 | LOC101252186 | -1.714508268 | 0.002054865 |
| 101252194 | ERF-H7 | -5.469402618 | 2.42E-06 |
| 101252203 | LOC101252203 | -2.420946187 | 4.68E-05 |
| 101252238 | LOC101252238 | -3.183842985 | 0.006514238 |
| 101252244 | LOC101252244 | -2.648123867 | 6.00E-07 |
| 101252251 | LOC101252251 | -1.603615533 | 4.10E-06 |
| 101252259 | LOC101252259 | -1.18895472 | 0.004093974 |
| 101252264 | LOC101252264 | -3.946856894 | 3.28E-06 |
| 101252273 | LOC101252273 | -2.548012687 | 2.93E-04 |
| 101252274 | LOC101252274 | -1.965175502 | 6.12E-05 |
| 101252275 | LOC101252275 | -4.507704524 | 3.39E-15 |
| 101252290 | LOC101252290 | -2.801360004 | 0.001256585 |
| 101252294 | LOC101252294 | -3.452837808 | 9.92E-12 |
| 101252295 | LOC101252295 | -1.138598017 | 8.27E-04 |
| 101252310 | LOC101252310 | -1.546148021 | 4.22E-04 |
| 101252319 | LOC101252319 | -1.874521323 | 0.001809058 |
| 101252331 | LOC101252331 | -1.052155613 | 9.85E-04 |
| 101252338 | LOC101252338 | -2.386046509 | 0.003665494 |
| 101252343 | LOC101252343 | -1.772378691 | 1.20E-06 |
| 101252349 | LOC101252349 | -2.21313695 | 1.12E-04 |
| 101252372 | LOC101252372 | -5.152117551 | 0.008674456 |
| 101252377 | LOC101252377 | -1.761393092 | 0.001032432 |
| 101252385 | LOC101252385 | -2.048567432 | 0.002700199 |
| 101252389 | LOC101252389 | -2.111634746 | 0.003078514 |
| 101252392 | LOC101252392 | -10.1515011 | 3.33E-13 |
| 101252410 | LOC101252410 | -2.766671679 | 8.09E-04 |
| 101252430 | LOC101252430 | -1.940721535 | 7.55E-04 |
| 101252431 | LOC101252431 | -2.478311084 | 0.005172345 |
| 101252437 | LOC101252437 | -2.111140815 | 0.00102891 |
| 101252447 | LOC101252447 | -3.979581256 | 5.10E-05 |
| 101252458 | LOC101252458 | -2.6422273 | 7.92E-04 |
| 101252501 | LOC101252501 | -2.310449735 | 3.68E-06 |
| 101252505 | Cyp-3 | -4.290320462 | 1.19E-11 |
| 101252509 | LOC101252509 | -1.993036878 | 0.002056425 |
| 101252515 | LOC101252515 | -1.712311348 | 7.81E-05 |
| 101252526 | LOC101252526 | -1.956655883 | 0.002864267 |
| 101252528 | LOC101252528 | -2.521313291 | 0.005403938 |
| 101252534 | LOC101252534 | -2.745520355 | 0.009068213 |
| 101252539 | LOC101252539 | -1.202166332 | 1.02E-04 |
| 101252561 | LOC101252561 | -1.776369506 | 9.32E-04 |
| 101252574 | LOC101252574 | -5.322926025 | 1.70E-24 |
| 101252583 | LOC101252583 | -3.36888671 | 1.06E-04 |
| 101252637 | LOC101252637 | -1.71896709 | 0.001125624 |
| 101252647 | LOC101252647 | -2.054857924 | 0.002237957 |
| 101252653 | LOC101252653 | -4.78674264 | 1.21E-06 |
| 101252673 | LOC101252673 | -3.286461545 | 0.001416722 |
| 101252677 | LOC101252677 | -3.125850733 | 2.87E-04 |
| 101252681 | LOC101252681 | -1.941671062 | 4.61E-04 |
| 101252685 | LOC101252685 | -3.341068488 | 1.30E-04 |
| 101252698 | LOC101252698 | -2.953024498 | 2.31E-05 |
| 101252717 | LOC101252717 | -2.89322549 | 4.41E-04 |
| 101252750 | LOC101252750 | -1.842354873 | 3.12E-04 |
| 101252751 | LOC101252751 | -1.559861959 | 0.005063903 |
| 101252785 | LOC101252785 | -2.773009012 | 2.61E-04 |
| 101252788 | LOC101252788 | -8.571365512 | 3.36E-19 |
| 101252815 | LOC101252815 | -2.853996342 | 0.001094572 |
| 101252818 | LOC101252818 | -2.318945473 | 4.34E-04 |
| 101252837 | LOC101252837 | -1.660574475 | 0.002510699 |
| 101252847 | LOC101252847 | -2.758140167 | 6.85E-05 |
| 101252851 | PHO1a | -1.288731296 | 0.007434663 |
| 101252854 | LOC101252854 | -3.169155219 | 3.74E-05 |
| 101252884 | LOC101252884 | -3.968667525 | 0.001796702 |
| 101252889 | LOC101252889 | -1.703123134 | 3.92E-04 |
| 101252890 | LOC101252890 | -1.832025891 | 4.39E-04 |
| 101252896 | LOC101252896 | -3.071604613 | 1.88E-07 |
| 101252905 | LOC101252905 | -3.520160461 | 8.96E-11 |
| 101252907 | LOC101252907 | -3.829056567 | 0.004579319 |
| 101252922 | LOC101252922 | -1.572893925 | 9.28E-05 |
| 101252931 | LOC101252931 | -1.151487587 | 0.002510905 |
| 101252947 | LOC101252947 | -1.536418857 | 3.50E-04 |
| 101252975 | LOC101252975 | -5.734961808 | 8.90E-04 |
| 101252987 | LOC101252987 | -2.612529404 | 0.001233721 |
| 101253010 | LOC101253010 | -1.509317227 | 3.49E-05 |
| 101253016 | LOC101253016 | -1.011798284 | 0.002900973 |
| 101253024 | LOC101253024 | -1.89804018 | 1.57E-05 |
| 101253028 | LOC101253028 | -1.053534066 | 0.005840603 |
| 101253049 | LOC101253049 | -3.136221813 | 6.96E-07 |
| 101253055 | LOC101253055 | -1.785641351 | 1.79E-04 |
| 101253061 | LOC101253061 | -1.808596854 | 3.67E-05 |
| 101253076 | LOC101253076 | -1.431717164 | 0.002944348 |
| 101253145 | LOC101253145 | -3.177368069 | 1.04E-08 |
| 101253159 | LOC101253159 | -2.188751037 | 2.38E-05 |
| 101253176 | LOC101253176 | -5.6448067 | 1.17E-12 |
| 101253180 | LOC101253180 | -3.231169948 | 7.68E-13 |
| 101253191 | LOC101253191 | -2.350684983 | 3.02E-04 |
| 101253208 | LOC101253208 | -5.609855904 | 0.004261674 |
| 101253214 | LOC101253214 | -1.598418485 | 0.00140842 |
| 101253224 | LOC101253224 | -3.263096446 | 7.95E-05 |
| 101253235 | LOC101253235 | -1.375772604 | 0.002785646 |
| 101253281 | LOC101253281 | -2.620930918 | 1.39E-06 |
| 101253302 | LOC101253302 | -1.048722122 | 8.04E-05 |
| 101253308 | LOC101253308 | -3.257809286 | 0.001757445 |
| 101253310 | LOC101253310 | -1.417795615 | 0.003180125 |
| 101253321 | LOC101253321 | -1.60932075 | 0.001510898 |
| 101253330 | LOC101253330 | -1.543677623 | 0.007897636 |
| 101253340 | LOC101253340 | -1.830881752 | 2.12E-04 |
| 101253374 | LOC101253374 | -1.72826378 | 0.004516644 |
| 101253377 | LOC101253377 | -1.452004333 | 8.97E-04 |
| 101253378 | LOC101253378 | -1.607706011 | 0.005969126 |
| 101253388 | LOC101253388 | -2.356943932 | 0.001245907 |
| 101253399 | LOC101253399 | -1.616724812 | 0.002114639 |
| 101253412 | LOC101253412 | -1.55480819 | 0.005093913 |
| 101253425 | LOC101253425 | -1.595847647 | 8.36E-04 |
| 101253436 | LOC101253436 | -2.927378739 | 1.93E-04 |
| 101253472 | LOC101253472 | -2.50203708 | 1.30E-08 |
| 101253493 | LOC101253493 | -1.089647594 | 0.001982499 |
| 101253500 | LOC101253500 | -4.096174246 | 0.00351355 |
| 101253552 | LOC101253552 | -2.173492587 | 2.43E-04 |
| 101253557 | LOC101253557 | -1.805847159 | 2.10E-04 |
| 101253581 | LOC101253581 | -1.818463582 | 0.003401969 |
| 101253582 | LOC101253582 | -1.904681234 | 9.19E-08 |
| 101253609 | LOC101253609 | -1.427599701 | 0.004328144 |
| 101253637 | LOC101253637 | -2.195073894 | 0.001457479 |
| 101253646 | LOC101253646 | -2.722289096 | 0.001277823 |
| 101253678 | LOC101253678 | -1.403348193 | 5.42E-04 |
| 101253684 | LOC101253684 | -1.769959952 | 0.002549304 |
| 101253693 | LOC101253693 | -1.146315357 | 9.71E-04 |
| 101253704 | LOC101253704 | -1.251879175 | 6.76E-04 |
| 101253723 | LOC101253723 | -4.884219627 | 9.66E-05 |
| 101253732 | LOC101253732 | -1.403904805 | 1.99E-04 |
| 101253738 | LOC101253738 | -3.24494864 | 5.28E-05 |
| 101253741 | LOC101253741 | -1.749555717 | 0.005743175 |
| 101253746 | LOC101253746 | -1.158396845 | 0.001264072 |
| 101253763 | LOC101253763 | -2.312584643 | 7.46E-04 |
| 101253780 | LOC101253780 | -5.74300119 | 6.33E-20 |
| 101253781 | LOC101253781 | -5.267645215 | 6.56E-13 |
| 101253787 | LOC101253787 | -1.24117636 | 2.57E-06 |
| 101253792 | LOC101253792 | -1.595443546 | 0.00339676 |
| 101253847 | LOC101253847 | -1.926755444 | 0.001281042 |
| 101253853 | LOC101253853 | -2.058289778 | 4.57E-07 |
| 101253856 | LOC101253856 | -3.398704632 | 4.20E-08 |
| 101253863 | LOC101253863 | -2.288245993 | 4.15E-04 |
| 101253882 | LOC101253882 | -1.324929085 | 0.007125897 |
| 101253887 | LOC101253887 | -1.747515827 | 0.001500777 |
| 101253889 | LOC101253889 | -6.901116878 | 3.17E-19 |
| 101253895 | LOC101253895 | -2.482691124 | 2.32E-06 |
| 101253917 | LOC101253917 | -1.663389661 | 0.004004432 |
| 101253920 | LOC101253920 | -1.81319353 | 0.004055658 |
| 101253936 | LOC101253936 | -2.154776832 | 4.25E-05 |
| 101253954 | LOC101253954 | -5.7419187 | 0.004015456 |
| 101253967 | LOC101253967 | -2.901481762 | 1.28E-04 |
| 101253985 | LOC101253985 | -2.375222753 | 2.86E-04 |
| 101253993 | LOC101253993 | -1.865862181 | 4.28E-04 |
| 101254008 | LOC101254008 | -1.519615709 | 1.28E-04 |
| 101254009 | LOC101254009 | -2.111494127 | 0.008641243 |
| 101254020 | LOC101254020 | -1.407917285 | 0.005688984 |
| 101254024 | LOC101254024 | -1.006462126 | 0.003894743 |
| 101254031 | LOC101254031 | -1.571961134 | 0.004551336 |
| 101254035 | LOC101254035 | -3.93457601 | 1.66E-04 |
| 101254053 | LOC101254053 | -1.379935743 | 6.80E-04 |
| 101254060 | LOC101254060 | -2.700301089 | 4.91E-04 |
| 101254073 | LOC101254073 | -3.566123817 | 0.007696473 |
| 101254074 | LOC101254074 | -2.059487694 | 0.004125418 |
| 101254075 | LOC101254075 | -2.613044636 | 6.76E-04 |
| 101254087 | LOC101254087 | -2.094167498 | 5.29E-08 |
| 101254090 | LOC101254090 | -4.797301679 | 1.13E-22 |
| 101254135 | LOC101254135 | -1.342499 | 1.54E-05 |
| 101254137 | LOC101254137 | -2.004212666 | 8.79E-05 |
| 101254153 | CD1 | -2.486010455 | 0.001651496 |
| 101254174 | LOC101254174 | -2.645739456 | 1.60E-08 |
| 101254224 | LOC101254224 | -1.49636023 | 0.004361495 |
| 101254232 | LOC101254232 | -2.972585418 | 2.85E-09 |
| 101254239 | LOC101254239 | -5.128394415 | 5.09E-16 |
| 101254244 | LOC101254244 | -1.461567639 | 1.77E-04 |
| 101254246 | LOC101254246 | -2.201352566 | 2.72E-05 |
| 101254249 | LOC101254249 | -3.444771127 | 1.48E-05 |
| 101254267 | LOC101254267 | -3.487369573 | 1.19E-06 |
| 101254285 | LOC101254285 | -1.45120859 | 0.006072007 |
| 101254304 | DCL2a | -1.143449398 | 0.008074441 |
| 101254313 | LOC101254313 | -1.193972127 | 1.42E-04 |
| 101254329 | LOC101254329 | -1.250651304 | 2.62E-06 |
| 101254332 | LOC101254332 | -3.471335109 | 3.37E-08 |
| 101254359 | ABCI9 | -1.062115184 | 0.003547621 |
| 101254372 | LOC101254372 | -2.150708507 | 0.001368303 |
| 101254383 | LOC101254383 | -2.131765982 | 0.003722821 |
| 101254385 | LOC101254385 | -2.096630674 | 6.69E-08 |
| 101254392 | LOC101254392 | -1.98318327 | 3.77E-05 |
| 101254395 | LOC101254395 | -2.872142068 | 3.93E-04 |
| 101254402 | GAME17 | -4.153191774 | 2.52E-06 |
| 101254425 | LOC101254425 | -4.577808945 | 1.51E-25 |
| 101254427 | LOC101254427 | -3.439143541 | 5.44E-07 |
| 101254465 | LOC101254465 | -1.32379474 | 0.004745071 |
| 101254500 | LOC101254500 | -2.636919197 | 7.09E-04 |
| 101254501 | LOC101254501 | -3.63348924 | 5.50E-06 |
| 101254521 | LOC101254521 | -3.829640949 | 1.74E-04 |
| 101254527 | LOC101254527 | -3.719630785 | 3.90E-07 |
| 101254539 | LOC101254539 | -2.089378492 | 1.43E-07 |
| 101254541 | LOC101254541 | -1.72972057 | 3.25E-06 |
| 101254547 | LOC101254547 | -2.683382613 | 0.008140906 |
| 101254581 | LOC101254581 | -2.291440554 | 7.17E-10 |
| 101254591 | LOC101254591 | -2.703126718 | 5.21E-08 |
| 101254596 | LOC101254596 | -1.311116724 | 0.004397772 |
| 101254619 | LOC101254619 | -2.501009131 | 9.81E-05 |
| 101254638 | LOC101254638 | -4.1688826 | 2.89E-12 |
| 101254639 | LOC101254639 | -1.776610691 | 0.003058945 |
| 101254642 | LOC101254642 | -2.116546677 | 6.45E-11 |
| 101254646 | LOC101254646 | -1.60679927 | 0.004016439 |
| 101254657 | LOC101254657 | -3.074457697 | 2.61E-05 |
| 101254658 | LOC101254658 | -1.568125658 | 1.56E-06 |
| 101254663 | LOC101254663 | -3.339380513 | 2.97E-06 |
| 101254692 | LOC101254692 | -1.251584518 | 0.006330839 |
| 101254693 | LOC101254693 | -2.367221395 | 1.27E-04 |
| 101254713 | LOC101254713 | -4.180583255 | 2.80E-06 |
| 101254717 | LOC101254717 | -2.024546559 | 1.59E-04 |
| 101254764 | LOC101254764 | -1.749234438 | 9.93E-06 |
| 101254841 | LOC101254841 | -2.224714312 | 1.51E-05 |
| 101254886 | LOC101254886 | -2.402075077 | 9.21E-07 |
| 101254913 | LOC101254913 | -2.988216219 | 1.73E-04 |
| 101254918 | LOC101254918 | -4.225380216 | 3.44E-06 |
| 101254924 | LOC101254924 | -2.247265113 | 4.52E-04 |
| 101254964 | LOC101254964 | -3.501525317 | 8.76E-04 |
| 101254968 | LOC101254968 | -3.431907076 | 0.005719775 |
| 101254971 | LOC101254971 | -1.971502242 | 2.37E-07 |
| 101254989 | ABCG7 | -1.922607848 | 0.001111377 |
| 101254992 | LOC101254992 | -1.690721671 | 1.85E-04 |
| 101254999 | LOC101254999 | -1.239276221 | 0.003305119 |
| 101255031 | LOC101255031 | -2.112235006 | 0.002110944 |
| 101255032 | LOC101255032 | -2.059138793 | 2.05E-05 |
| 101255070 | LOC101255070 | -1.920400829 | 0.0031805 |
| 101255077 | LOC101255077 | -1.240614064 | 0.002222592 |
| 101255089 | AS | -1.59958741 | 1.43E-04 |
| 101255122 | LOC101255122 | -2.571498005 | 0.007075215 |
| 101255128 | LOC101255128 | -1.246830118 | 6.55E-04 |
| 101255154 | LOC101255154 | -3.226414782 | 7.74E-05 |
| 101255162 | LOC101255162 | -1.445204678 | 3.55E-07 |
| 101255169 | LOC101255169 | -2.239164167 | 1.87E-07 |
| 101255211 | LOC101255211 | -1.573288592 | 0.006831934 |
| 101255223 | LOC101255223 | -3.613669074 | 2.10E-05 |
| 101255226 | P69d | -1.966151177 | 1.49E-05 |
| 101255236 | LOC101255236 | -1.221886587 | 2.28E-05 |
| 101255242 | LOC101255242 | -2.991066082 | 0.001098742 |
| 101255248 | LOC101255248 | -4.094540623 | 6.12E-11 |
| 101255263 | LOC101255263 | -1.974373928 | 2.14E-05 |
| 101255270 | LOC101255270 | -1.899109352 | 0.00340971 |
| 101255272 | LOC101255272 | -7.50339483 | 1.21E-07 |
| 101255274 | LOC101255274 | -3.484647816 | 0.001023 |
| 101255313 | LOC101255313 | -1.738819781 | 0.006481308 |
| 101255326 | LOC101255326 | -1.854462139 | 2.55E-09 |
| 101255332 | LOC101255332 | -5.208349434 | 0.001013036 |
| 101255334 | LOC101255334 | -2.487992108 | 0.001070487 |
| 101255335 | LOC101255335 | -2.734557622 | 3.64E-05 |
| 101255361 | LOC101255361 | -1.750525722 | 0.004084982 |
| 101255377 | LOC101255377 | -1.496372361 | 0.008790986 |
| 101255379 | LOC101255379 | -3.60022509 | 4.70E-08 |
| 101255382 | LOC101255382 | -1.335627076 | 0.005340718 |
| 101255394 | LOC101255394 | -1.144347762 | 0.006592277 |
| 101255413 | LOC101255413 | -1.556853245 | 0.001945397 |
| 101255427 | LOC101255427 | -2.437719231 | 2.91E-04 |
| 101255428 | LOC101255428 | -1.386756974 | 0.006539907 |
| 101255436 | LOC101255436 | -1.83933488 | 0.004150682 |
| 101255440 | LOC101255440 | -2.3603849 | 7.22E-04 |
| 101255463 | LOC101255463 | -3.177436127 | 3.03E-06 |
| 101255487 | LOC101255487 | -2.280432623 | 0.002883358 |
| 101255511 | LOC101255511 | -4.331441839 | 0.001806923 |
| 101255533 | LOC101255533 | -1.775070405 | 7.62E-05 |
| 101255543 | LOC101255543 | -1.837680545 | 2.86E-04 |
| 101255590 | LOC101255590 | -21.50165801 | 3.80E-08 |
| 101255593 | LOC101255593 | -1.804639769 | 8.14E-05 |
| 101255594 | LOC101255594 | -1.561870028 | 0.004010609 |
| 101255597 | LOC101255597 | -1.524431436 | 0.001438529 |
| 101255616 | LOC101255616 | -4.64954918 | 2.26E-04 |
| 101255627 | LOC101255627 | -2.976665086 | 3.87E-05 |
| 101255645 | LOC101255645 | -2.83618463 | 4.84E-09 |
| 101255673 | LOC101255673 | -8.01672355 | 1.20E-08 |
| 101255674 | LOC101255674 | -2.756172655 | 1.35E-11 |
| 101255675 | LOC101255675 | -2.115406629 | 0.001711256 |
| 101255712 | LOC101255712 | -6.733822525 | 2.28E-13 |
| 101255725 | LOC101255725 | -2.422165334 | 0.006078493 |
| 101255730 | LOC101255730 | -4.358592751 | 4.43E-06 |
| 101255733 | LOC101255733 | -5.343527665 | 0.004839351 |
| 101255734 | AnthOMT | -4.864240457 | 6.02E-07 |
| 101255743 | LOC101255743 | -2.101474405 | 0.00131567 |
| 101255756 | LOC101255756 | -1.695402105 | 1.44E-08 |
| 101255769 | LOC101255769 | -1.131055147 | 0.004647309 |
| 101255776 | LOC101255776 | -3.577750829 | 2.34E-04 |
| 101255779 | LOC101255779 | -3.074283661 | 3.58E-06 |
| 101255788 | LOC101255788 | -3.637187619 | 3.49E-19 |
| 101255791 | LOC101255791 | -4.650587848 | 4.29E-06 |
| 101255806 | LOC101255806 | -4.396990549 | 1.94E-10 |
| 101255815 | LOC101255815 | -2.142186025 | 4.74E-04 |
| 101255817 | LOC101255817 | -4.232713773 | 1.30E-05 |
| 101255826 | LOC101255826 | -3.11296074 | 4.55E-11 |
| 101255843 | LOC101255843 | -2.049342801 | 0.006015121 |
| 101255858 | LOC101255858 | -1.377391166 | 2.35E-04 |
| 101255867 | LOC101255867 | -2.001656843 | 0.00117555 |
| 101255874 | LOC101255874 | -2.211788206 | 0.00587039 |
| 101255881 | LOC101255881 | -2.236208889 | 3.62E-05 |
| 101255907 | LOC101255907 | -3.186310118 | 2.16E-09 |
| 101255915 | LOC101255915 | -2.31147796 | 4.47E-09 |
| 101255922 | LOC101255922 | -2.440512689 | 0.004153742 |
| 101255936 | LOC101255936 | -3.717035007 | 1.19E-12 |
| 101255948 | LOC101255948 | -1.498439412 | 2.08E-08 |
| 101255983 | LOC101255983 | -1.935456241 | 3.10E-04 |
| 101255990 | LOC101255990 | -1.848157295 | 3.13E-04 |
| 101255991 | LOC101255991 | -1.387066953 | 0.007551076 |
| 101256028 | LOC101256028 | -1.502296402 | 1.47E-04 |
| 101256052 | LOC101256052 | -2.554480416 | 2.06E-06 |
| 101256056 | LOC101256056 | -1.67949248 | 7.02E-05 |
| 101256057 | LOC101256057 | -1.486013669 | 0.008259986 |
| 101256061 | LOC101256061 | -2.211476249 | 0.001641185 |
| 101256081 | LOC101256081 | -1.390365638 | 0.008957235 |
| 101256134 | LOC101256134 | -3.710670818 | 4.95E-05 |
| 101256148 | LOC101256148 | -2.441076649 | 9.34E-05 |
| 101256174 | LOC101256174 | -1.747011661 | 8.04E-06 |
| 101256200 | LOC101256200 | -2.296879189 | 2.84E-12 |
| 101256208 | LOC101256208 | -1.68608638 | 1.80E-06 |
| 101256216 | LOC101256216 | -2.438662756 | 1.30E-04 |
| 101256221 | LOC101256221 | -2.99840257 | 1.75E-04 |
| 101256225 | LOC101256225 | -2.751716036 | 1.80E-05 |
| 101256257 | LOC101256257 | -3.016178886 | 2.65E-04 |
| 101256279 | LOC101256279 | -2.319557205 | 0.001332323 |
| 101256280 | LOC101256280 | -2.392133229 | 6.95E-04 |
| 101256306 | LOC101256306 | -1.726707845 | 0.002598089 |
| 101256351 | LOC101256351 | -3.63692324 | 2.78E-05 |
| 101256378 | LOC101256378 | -1.837806477 | 0.002014811 |
| 101256391 | LOC101256391 | -4.295551314 | 1.81E-09 |
| 101256426 | FKBP20-2 | -2.730974902 | 5.81E-13 |
| 101256443 | HSD2 | -2.006581923 | 5.25E-04 |
| 101256444 | LOC101256444 | -1.419116723 | 7.62E-04 |
| 101256451 | LOC101256451 | -1.985176131 | 6.06E-06 |
| 101256453 | LOC101256453 | -1.732849311 | 2.75E-04 |
| 101256517 | LOC101256517 | -3.849863853 | 1.94E-13 |
| 101256520 | LOC101256520 | -1.639005525 | 0.007236102 |
| 101256521 | LOC101256521 | -1.793922468 | 6.46E-06 |
| 101256522 | LOC101256522 | -1.21613345 | 3.85E-04 |
| 101256541 | LOC101256541 | -1.059697704 | 0.006200878 |
| 101256542 | LOC101256542 | -2.406647102 | 0.004789165 |
| 101256554 | LOC101256554 | -1.66672405 | 7.03E-05 |
| 101256593 | LOC101256593 | -1.893469336 | 4.72E-07 |
| 101256601 | LOC101256601 | -3.558586682 | 1.15E-10 |
| 101256607 | LOC101256607 | -3.140746866 | 3.27E-04 |
| 101256626 | LOC101256626 | -4.47641814 | 1.96E-04 |
| 101256651 | LOC101256651 | -1.215123438 | 0.007129034 |
| 101256653 | LOC101256653 | -2.134399074 | 0.005014496 |
| 101256663 | LOC101256663 | -3.082015596 | 1.59E-04 |
| 101256690 | LOC101256690 | -1.894811858 | 1.17E-08 |
| 101256694 | LOC101256694 | -2.032661192 | 0.003775498 |
| 101256708 | LOC101256708 | -2.771350331 | 4.21E-05 |
| 101256734 | LOC101256734 | -2.037860207 | 0.002031522 |
| 101256738 | LOC101256738 | -3.643942817 | 6.74E-05 |
| 101256762 | LOC101256762 | -1.951045944 | 1.79E-04 |
| 101256783 | LOC101256783 | -2.643258808 | 0.001474481 |
| 101256806 | LOC101256806 | -1.946921845 | 1.17E-05 |
| 101256812 | LOC101256812 | -3.48924008 | 4.78E-05 |
| 101256821 | LOC101256821 | -2.303338585 | 9.92E-05 |
| 101256822 | LOC101256822 | -1.594970603 | 1.50E-04 |
| 101256838 | LOC101256838 | -1.135304267 | 0.004737872 |
| 101256886 | CPT7 | -2.451611041 | 8.58E-05 |
| 101256887 | LOC101256887 | -2.067239751 | 4.57E-04 |
| 101256908 | LOC101256908 | -6.079498068 | 1.68E-35 |
| 101256920 | LOC101256920 | -1.970658029 | 0.00528115 |
| 101256933 | LOC101256933 | -1.624947439 | 1.27E-05 |
| 101256940 | LOC101256940 | -2.45971597 | 1.43E-04 |
| 101256941 | LOC101256941 | -2.84636137 | 0.001057058 |
| 101256944 | LOC101256944 | -5.068705161 | 1.43E-11 |
| 101256952 | LOC101256952 | -3.4381792 | 9.47E-06 |
| 101256964 | LOC101256964 | -1.208035563 | 0.005443248 |
| 101256971 | LOC101256971 | -3.626707365 | 6.85E-07 |
| 101256992 | LOC101256992 | -3.034081128 | 2.82E-05 |
| 101256999 | LOC101256999 | -1.495386731 | 1.27E-04 |
| 101257007 | LOC101257007 | -1.902022615 | 5.50E-06 |
| 101257011 | LOC101257011 | -1.941276498 | 0.001330767 |
| 101257019 | LOC101257019 | -1.793145808 | 0.007885906 |
| 101257020 | LOC101257020 | -1.661303046 | 0.004577844 |
| 101257029 | LOC101257029 | -2.588108006 | 0.001553644 |
| 101257038 | LOC101257038 | -2.839524468 | 0.004166942 |
| 101257044 | LOC101257044 | -1.58683121 | 1.33E-05 |
| 101257062 | LOC101257062 | -2.714100387 | 3.02E-06 |
| 101257068 | LOC101257068 | -2.877046448 | 6.07E-06 |
| 101257089 | LOC101257089 | -3.314886799 | 2.38E-05 |
| 101257102 | LOC101257102 | -1.359466193 | 0.007413189 |
| 101257105 | LOC101257105 | -3.498773658 | 1.71E-04 |
| 101257114 | LOC101257114 | -1.179862714 | 0.006176756 |
| 101257131 | LOC101257131 | -1.658952909 | 4.37E-07 |
| 101257137 | LOC101257137 | -3.233991903 | 1.51E-04 |
| 101257144 | LOC101257144 | -1.151561592 | 0.004039591 |
| 101257156 | LOC101257156 | -7.693920029 | 4.83E-20 |
| 101257158 | LOC101257158 | -1.112697555 | 0.006365161 |
| 101257192 | LOC101257192 | -4.813629922 | 1.17E-07 |
| 101257203 | LOC101257203 | -2.896092155 | 1.79E-06 |
| 101257214 | LOC101257214 | -1.445657143 | 0.009186074 |
| 101257216 | LOC101257216 | -1.653293729 | 1.31E-04 |
| 101257222 | LOC101257222 | -4.543862057 | 0.001820388 |
| 101257241 | LOC101257241 | -1.314753407 | 0.001445155 |
| 101257275 | LOC101257275 | -1.464427751 | 0.001969054 |
| 101257289 | LOC101257289 | -4.737632447 | 0.002382314 |
| 101257308 | LOC101257308 | -1.674737793 | 4.54E-07 |
| 101257321 | LOC101257321 | -3.849445213 | 4.31E-05 |
| 101257326 | LOC101257326 | -1.893018767 | 4.77E-04 |
| 101257328 | LOC101257328 | -2.416566919 | 4.25E-05 |
| 101257335 | LOC101257335 | -1.796293779 | 0.003228859 |
| 101257347 | LOC101257347 | -1.050136751 | 0.004686589 |
| 101257349 | LOC101257349 | -2.413424649 | 1.51E-06 |
| 101257365 | LOC101257365 | -3.048619177 | 3.91E-04 |
| 101257393 | LOC101257393 | -1.38267557 | 0.003080208 |
| 101257400 | LOC101257400 | -1.329608015 | 0.002070977 |
| 101257440 | LOC101257440 | -1.254183608 | 3.81E-06 |
| 101257448 | LOC101257448 | -1.365785633 | 0.005207657 |
| 101257452 | LOC101257452 | -2.15759166 | 6.87E-04 |
| 101257480 | LOC101257480 | -1.220146892 | 4.65E-04 |
| 101257507 | LOC101257507 | -1.782975099 | 0.003154205 |
| 101257523 | LOC101257523 | -1.671025904 | 1.52E-04 |
| 101257532 | CYP77B11 | -4.002373867 | 3.74E-06 |
| 101257536 | LOC101257536 | -1.176956265 | 6.37E-04 |
| 101257602 | LOC101257602 | -2.226264484 | 1.22E-05 |
| 101257603 | LOC101257603 | -2.31305012 | 9.93E-05 |
| 101257609 | LOC101257609 | -1.537024616 | 0.007439397 |
| 101257630 | LOC101257630 | -1.344452392 | 0.007133655 |
| 101257635 | LOC101257635 | -2.751526761 | 8.66E-05 |
| 101257654 | LOC101257654 | -1.192929146 | 0.003430666 |
| 101257661 | LOC101257661 | -1.433909605 | 3.23E-04 |
| 101257662 | LOC101257662 | -1.11381488 | 0.008236345 |
| 101257682 | LOC101257682 | -1.309745844 | 0.003142473 |
| 101257693 | LOC101257693 | -2.75802445 | 3.28E-06 |
| 101257697 | LOC101257697 | -2.473826313 | 9.80E-04 |
| 101257699 | LOC101257699 | -2.44671134 | 5.77E-05 |
| 101257739 | LOC101257739 | -3.236059026 | 4.12E-08 |
| 101257748 | LOC101257748 | -2.717835094 | 0.001172758 |
| 101257754 | LOC101257754 | -3.158776348 | 7.03E-11 |
| 101257774 | LOC101257774 | -2.990864364 | 0.005034451 |
| 101257795 | LOC101257795 | -5.880930272 | 6.79E-12 |
| 101257813 | LOC101257813 | -1.961084404 | 2.11E-05 |
| 101257827 | LOC101257827 | -1.699524695 | 0.004477306 |
| 101257849 | LOC101257849 | -4.833580704 | 5.45E-23 |
| 101257851 | LOC101257851 | -1.807281999 | 0.004153048 |
| 101257855 | LOC101257855 | -1.535916136 | 1.65E-04 |
| 101257882 | LOC101257882 | -5.530935129 | 7.35E-07 |
| 101257899 | LOC101257899 | -1.656277539 | 0.001198923 |
| 101257904 | LOC101257904 | -2.087112213 | 0.001593299 |
| 101257924 | LOC101257924 | -1.993577158 | 1.30E-10 |
| 101257930 | LOC101257930 | -5.765963594 | 3.25E-15 |
| 101257931 | LOC101257931 | -1.039821546 | 2.09E-04 |
| 101257957 | LOC101257957 | -2.0071669 | 0.001376548 |
| 101257962 | LOC101257962 | -1.463096087 | 1.41E-05 |
| 101257963 | LOC101257963 | -1.095956893 | 0.005453828 |
| 101257972 | LOC101257972 | -1.574784432 | 0.00726874 |
| 101258020 | LOC101258020 | -1.884406965 | 2.20E-05 |
| 101258022 | LOC101258022 | -1.841085869 | 0.001274382 |
| 101258029 | LOC101258029 | -5.325459417 | 2.96E-12 |
| 101258031 | LOC101258031 | -1.478238699 | 0.005989111 |
| 101258035 | LOC101258035 | -5.275572253 | 1.41E-37 |
| 101258043 | LOC101258043 | -8.69394199 | 1.45E-09 |
| 101258070 | LOC101258070 | -1.898426261 | 5.81E-04 |
| 101258080 | LOC101258080 | -2.453149615 | 1.02E-07 |
| 101258089 | LOC101258089 | -3.276639939 | 5.61E-04 |
| 101258100 | LOC101258100 | -6.265094664 | 2.57E-09 |
| 101258110 | LOC101258110 | -2.560722911 | 1.27E-04 |
| 101258137 | LOC101258137 | -3.873895311 | 0.005252284 |
| 101258138 | LOC101258138 | -2.307468843 | 1.36E-06 |
| 101258142 | LOC101258142 | -2.058350926 | 0.001052638 |
| 101258150 | LOC101258150 | -1.13277143 | 2.79E-04 |
| 101258152 | LOC101258152 | -3.441020092 | 9.67E-05 |
| 101258158 | LOC101258158 | -1.917318818 | 0.00424272 |
| 101258160 | LOC101258160 | -2.408457871 | 3.10E-07 |
| 101258163 | LOC101258163 | -2.040683601 | 0.001270403 |
| 101258170 | LOC101258170 | -2.049869141 | 2.01E-10 |
| 101258206 | LOC101258206 | -2.690853319 | 1.05E-04 |
| 101258225 | LOC101258225 | -3.992049755 | 0.005955937 |
| 101258234 | LOC101258234 | -2.787905918 | 1.12E-04 |
| 101258235 | LOC101258235 | -2.980951432 | 0.001194159 |
| 101258242 | LOC101258242 | -2.470623964 | 4.11E-04 |
| 101258243 | LOC101258243 | -2.204453352 | 0.002527021 |
| 101258259 | LOC101258259 | -2.477813745 | 4.39E-06 |
| 101258273 | LOC101258273 | -1.374164866 | 0.003217466 |
| 101258281 | LOC101258281 | -1.310513575 | 7.75E-05 |
| 101258304 | LOC101258304 | -2.474366221 | 2.00E-06 |
| 101258323 | LOC101258323 | -3.517027662 | 0.001738154 |
| 101258334 | LOC101258334 | -2.426081401 | 4.78E-08 |
| 101258346 | LOC101258346 | -2.2826628 | 5.18E-06 |
| 101258352 | LOC101258352 | -2.134491834 | 0.005775941 |
| 101258372 | LOC101258372 | -2.060032747 | 0.006064455 |
| 101258376 | LOC101258376 | -3.542079876 | 1.47E-07 |
| 101258383 | TRM5 | -1.468569829 | 1.67E-04 |
| 101258472 | LOC101258472 | -3.404358446 | 5.76E-07 |
| 101258477 | LOC101258477 | -2.270366386 | 3.60E-04 |
| 101258483 | LOC101258483 | -1.250038339 | 0.002136533 |
| 101258497 | LOC101258497 | -2.316225668 | 7.29E-09 |
| 101258502 | LOC101258502 | -1.276909452 | 4.55E-05 |
| 101258506 | LOC101258506 | -1.290730296 | 0.007087241 |
| 101258510 | LOC101258510 | -1.803920992 | 0.002927455 |
| 101258542 | LOC101258542 | -2.334169823 | 0.006982851 |
| 101258570 | LOC101258570 | -2.123578071 | 4.79E-04 |
| 101258601 | LOC101258601 | -1.397793848 | 0.00820965 |
| 101258612 | LOC101258612 | -2.324054163 | 1.06E-05 |
| 101258615 | LOC101258615 | -2.180361454 | 7.96E-04 |
| 101258622 | LOC101258622 | -2.248690831 | 0.001172416 |
| 101258635 | LOC101258635 | -2.132721494 | 0.005540053 |
| 101258638 | LOC101258638 | -1.979401362 | 4.34E-04 |
| 101258646 | LOC101258646 | -4.126872771 | 1.45E-06 |
| 101258662 | LOC101258662 | -1.7986967 | 5.94E-05 |
| 101258667 | LOC101258667 | -3.046743967 | 3.44E-05 |
| 101258672 | LOC101258672 | -1.03550461 | 0.008601957 |
| 101258691 | LOC101258691 | -1.995320613 | 7.70E-04 |
| 101258746 | LOC101258746 | -2.093824214 | 4.34E-04 |
| 101258748 | LOC101258748 | -2.248243685 | 9.64E-05 |
| 101258756 | LOC101258756 | -2.011524532 | 4.26E-11 |
| 101258774 | PPO | -5.37834356 | 5.64E-07 |
| 101258777 | LOC101258777 | -1.295575728 | 0.001543229 |
| 101258780 | LOC101258780 | -1.987704017 | 3.11E-04 |
| 101258781 | LOC101258781 | -2.698152383 | 5.65E-12 |
| 101258811 | LOC101258811 | -1.553725574 | 0.00279922 |
| 101258831 | LOC101258831 | -1.117614485 | 0.002911464 |
| 101258844 | LOC101258844 | -1.878770858 | 3.23E-07 |
| 101258850 | LOC101258850 | -1.781226285 | 0.007109767 |
| 101258865 | LOC101258865 | -2.748437422 | 1.94E-08 |
| 101258872 | LOC101258872 | -2.838185272 | 6.64E-15 |
| 101258882 | LOC101258882 | -3.895241223 | 0.006117061 |
| 101258890 | LOC101258890 | -1.911674909 | 0.006355152 |
| 101258898 | LOC101258898 | -1.261474997 | 0.006771869 |
| 101258914 | LOC101258914 | -1.170344854 | 0.00861795 |
| 101258925 | LOC101258925 | -1.523892922 | 9.36E-06 |
| 101258934 | LOC101258934 | -1.467618711 | 2.23E-04 |
| 101258936 | LOC101258936 | -1.599660039 | 7.26E-05 |
| 101258946 | LOC101258946 | -3.52620824 | 0.002020918 |
| 101258951 | LOC101258951 | -4.470965543 | 4.81E-06 |
| 101258959 | LOC101258959 | -1.308647784 | 9.38E-04 |
| 101258960 | LOC101258960 | -1.8882445 | 0.001147957 |
| 101258971 | LOC101258971 | -3.432830703 | 5.56E-08 |
| 101258999 | LOC101258999 | -1.436172526 | 0.001673145 |
| 101259028 | LOC101259028 | -2.07763659 | 1.69E-06 |
| 101259050 | MHX | -1.532274193 | 1.23E-04 |
| 101259064 | LOC101259064 | -5.870548238 | 4.77E-07 |
| 101259079 | LOC101259079 | -2.352076415 | 2.10E-06 |
| 101259080 | LOC101259080 | -2.214505896 | 5.61E-04 |
| 101259105 | LOC101259105 | -1.728476767 | 2.20E-05 |
| 101259111 | LOC101259111 | -3.813295684 | 7.61E-08 |
| 101259112 | LOC101259112 | -2.59812858 | 4.31E-06 |
| 101259141 | LOC101259141 | -1.398498766 | 5.49E-04 |
| 101259149 | LOC101259149 | -2.42186038 | 3.20E-05 |
| 101259151 | LOC101259151 | -1.62577567 | 1.22E-06 |
| 101259161 | LOC101259161 | -3.398572182 | 2.07E-11 |
| 101259172 | LOC101259172 | -3.761681383 | 2.76E-05 |
| 101259185 | LOC101259185 | -3.428190841 | 4.82E-04 |
| 101259187 | LOC101259187 | -3.070209229 | 4.78E-04 |
| 101259189 | LOC101259189 | -1.764097861 | 3.14E-05 |
| 101259221 | LOC101259221 | -3.577644958 | 2.95E-10 |
| 101259226 | LOC101259226 | -1.848944962 | 0.007048179 |
| 101259227 | LOC101259227 | -1.521883521 | 0.001066639 |
| 101259237 | LOC101259237 | -1.577703492 | 0.007253794 |
| 101259258 | LOC101259258 | -1.172729435 | 0.00462067 |
| 101259262 | LOC101259262 | -3.094634494 | 1.95E-07 |
| 101259280 | LOC101259280 | -1.977149923 | 2.12E-04 |
| 101259298 | LOC101259298 | -1.679928336 | 7.35E-04 |
| 101259307 | LOC101259307 | -2.306865822 | 5.53E-08 |
| 101259323 | ERF-H6 | -3.149811391 | 3.10E-06 |
| 101259352 | LOC101259352 | -2.906961052 | 2.85E-06 |
| 101259357 | LOC101259357 | -3.442944241 | 1.67E-06 |
| 101259358 | LOC101259358 | -1.843534505 | 0.001737748 |
| 101259405 | LOC101259405 | -1.503696593 | 0.002197249 |
| 101259429 | LOC101259429 | -2.654881995 | 0.008330918 |
| 101259433 | LOC101259433 | -1.027381789 | 0.006769274 |
| 101259446 | LOC101259446 | -2.659431476 | 1.24E-05 |
| 101259457 | LOC101259457 | -2.512808738 | 0.002950138 |
| 101259486 | LOC101259486 | -2.373051538 | 5.24E-07 |
| 101259500 | LOC101259500 | -2.856209424 | 2.40E-04 |
| 101259523 | LOC101259523 | -2.993598341 | 3.10E-04 |
| 101259524 | LOC101259524 | -2.450257818 | 4.37E-08 |
| 101259534 | LOC101259534 | -3.357836722 | 0.005616693 |
| 101259555 | LOC101259555 | -6.364650668 | 6.91E-31 |
| 101259562 | LOC101259562 | -4.473369467 | 1.49E-51 |
| 101259566 | LOC101259566 | -2.417475557 | 1.56E-04 |
| 101259611 | LOC101259611 | -1.324558634 | 0.003315379 |
| 101259650 | LOC101259650 | -1.176979723 | 7.10E-04 |
| 101259671 | LOC101259671 | -1.20185069 | 8.36E-04 |
| 101259676 | LOC101259676 | -1.464398603 | 0.004663881 |
| 101259681 | LOC101259681 | -1.689790912 | 4.79E-04 |
| 101259689 | LOC101259689 | -1.803568688 | 1.65E-10 |
| 101259699 | LOC101259699 | -1.748544684 | 1.66E-04 |
| 101259701 | LOC101259701 | -3.450140946 | 4.01E-04 |
| 101259728 | LOC101259728 | -2.894651679 | 5.33E-14 |
| 101259759 | LOC101259759 | -1.485798158 | 0.00361997 |
| 101259763 | LOC101259763 | -4.288622519 | 2.27E-05 |
| 101259783 | LOC101259783 | -3.479089704 | 4.42E-11 |
| 101259799 | CRTISO-L2 | -1.371920758 | 8.47E-07 |
| 101259803 | LOC101259803 | -3.365414589 | 3.73E-09 |
| 101259808 | LOC101259808 | -1.250920715 | 0.002212511 |
| 101259857 | LOC101259857 | -1.106898684 | 0.00676004 |
| 101259862 | LOC101259862 | -1.969821456 | 3.27E-04 |
| 101259864 | LOC101259864 | -1.430500691 | 2.44E-05 |
| 101259876 | LOC101259876 | -2.785563825 | 2.27E-07 |
| 101259880 | LOC101259880 | -2.9297588 | 3.21E-07 |
| 101259886 | LOC101259886 | -2.554078555 | 5.91E-04 |
| 101259914 | LOC101259914 | -3.054698596 | 0.003404764 |
| 101259915 | LOC101259915 | -2.48899385 | 8.49E-08 |
| 101259942 | LOC101259942 | -2.298351702 | 8.40E-05 |
| 101259959 | LOC101259959 | -2.46321509 | 1.27E-07 |
| 101259964 | LOC101259964 | -2.43002151 | 4.20E-05 |
| 101259980 | LOC101259980 | -2.338638861 | 0.001179017 |
| 101259995 | LOC101259995 | -1.055544588 | 0.004769742 |
| 101260002 | LOC101260002 | -2.552738183 | 2.39E-04 |
| 101260027 | LOC101260027 | -1.369685678 | 0.003576232 |
| 101260040 | MKS2b | -2.656343481 | 0.001258006 |
| 101260044 | Phyt2 | -3.809674951 | 7.99E-09 |
| 101260056 | LOC101260056 | -1.409371779 | 0.007388045 |
| 101260086 | LOC101260086 | -3.503399478 | 1.23E-05 |
| 101260113 | LOC101260113 | -3.051373707 | 0.001770947 |
| 101260128 | LOC101260128 | -2.266282874 | 2.28E-05 |
| 101260133 | LOC101260133 | -1.034163064 | 0.004638421 |
| 101260141 | LOC101260141 | -2.14381392 | 1.32E-04 |
| 101260169 | LOC101260169 | -1.852224201 | 0.00203473 |
| 101260191 | LOC101260191 | -1.540232647 | 0.008164593 |
| 101260197 | LOC101260197 | -2.966043818 | 2.31E-04 |
| 101260206 | LOC101260206 | -2.249316057 | 0.007175248 |
| 101260209 | LOC101260209 | -2.599527097 | 1.95E-09 |
| 101260218 | LOC101260218 | -1.490519394 | 4.14E-07 |
| 101260248 | LOC101260248 | -2.024878323 | 9.00E-06 |
| 101260250 | LOC101260250 | -1.432726906 | 5.13E-04 |
| 101260252 | LOC101260252 | -1.391946213 | 6.33E-04 |
| 101260262 | LOC101260262 | -1.19387945 | 0.003415941 |
| 101260266 | LOC101260266 | -1.585398848 | 0.00285935 |
| 101260274 | LOC101260274 | -1.134357291 | 0.008449281 |
| 101260290 | LOC101260290 | -3.179725291 | 9.67E-05 |
| 101260295 | LOC101260295 | -2.25586561 | 2.09E-04 |
| 101260303 | LOC101260303 | -1.24412622 | 0.005118538 |
| 101260335 | LOC101260335 | -3.139049433 | 3.27E-04 |
| 101260342 | LOC101260342 | -3.100298655 | 3.14E-04 |
| 101260349 | ccs52B | -3.294621134 | 6.14E-05 |
| 101260351 | LOC101260351 | -1.557990176 | 1.76E-09 |
| 101260364 | LOC101260364 | -2.876634363 | 1.82E-06 |
| 101260376 | LOC101260376 | -1.648504037 | 0.001773822 |
| 101260381 | LOC101260381 | -2.480097834 | 1.98E-09 |
| 101260385 | LOC101260385 | -1.163243786 | 4.48E-04 |
| 101260399 | LOC101260399 | -1.756544795 | 0.002836384 |
| 101260406 | LOC101260406 | -1.812031023 | 1.40E-08 |
| 101260456 | LOC101260456 | -2.954981214 | 8.20E-05 |
| 101260474 | LOC101260474 | -2.522303358 | 2.24E-07 |
| 101260476 | LOC101260476 | -1.304265227 | 2.21E-04 |
| 101260479 | LOC101260479 | -1.513744456 | 0.005213176 |
| 101260505 | LOC101260505 | -1.922003222 | 0.001780976 |
| 101260510 | LOC101260510 | -2.119927946 | 0.004956633 |
| 101260512 | LOC101260512 | -4.000205009 | 6.17E-12 |
| 101260516 | LOC101260516 | -1.836546033 | 0.002324993 |
| 101260542 | LOC101260542 | -1.715895419 | 5.60E-07 |
| 101260547 | LOC101260547 | -2.968499876 | 4.48E-08 |
| 101260571 | LOC101260571 | -2.73183345 | 0.002072759 |
| 101260572 | LOC101260572 | -1.909697238 | 9.50E-05 |
| 101260575 | LOC101260575 | -1.201609966 | 9.42E-04 |
| 101260578 | LOC101260578 | -1.89517756 | 6.65E-05 |
| 101260588 | LOC101260588 | -1.787168764 | 5.34E-05 |
| 101260612 | LOC101260612 | -1.57180009 | 0.005994883 |
| 101260638 | LOC101260638 | -1.817390446 | 8.05E-05 |
| 101260642 | LOC101260642 | -3.764763446 | 1.89E-05 |
| 101260662 | LOC101260662 | -1.767987898 | 0.005112836 |
| 101260664 | LOC101260664 | -1.624222781 | 1.75E-04 |
| 101260667 | LOC101260667 | -1.390651594 | 0.001998847 |
| 101260668 | LOC101260668 | -2.353106274 | 4.27E-06 |
| 101260676 | LOC101260676 | -1.825430694 | 5.57E-04 |
| 101260679 | LOC101260679 | -1.369935268 | 5.04E-05 |
| 101260697 | LOC101260697 | -2.910788307 | 2.75E-05 |
| 101260706 | LOC101260706 | -1.734405115 | 2.53E-06 |
| 101260718 | LOC101260718 | -1.52321484 | 1.85E-04 |
| 101260721 | LOC101260721 | -2.19909747 | 0.001189507 |
| 101260722 | LOC101260722 | -1.428706533 | 1.20E-04 |
| 101260734 | LOC101260734 | -2.631066981 | 0.00871878 |
| 101260770 | LOC101260770 | -1.073503621 | 0.007556938 |
| 101260787 | LOC101260787 | -2.99792788 | 4.91E-05 |
| 101260791 | LOC101260791 | -1.874261155 | 0.005095431 |
| 101260824 | LOC101260824 | -2.634371375 | 1.28E-07 |
| 101260831 | LOC101260831 | -2.368005007 | 2.56E-04 |
| 101260833 | LOC101260833 | -1.332203119 | 0.00300959 |
| 101260839 | LOC101260839 | -2.990302217 | 2.91E-07 |
| 101260869 | LOC101260869 | -3.543427721 | 2.29E-18 |
| 101260876 | LOC101260876 | -3.134690587 | 0.001734296 |
| 101260887 | LOC101260887 | -2.696306438 | 4.11E-04 |
| 101260889 | LOC101260889 | -1.141590018 | 0.001316043 |
| 101260892 | LOC101260892 | -1.467425522 | 0.004053262 |
| 101260911 | LOC101260911 | -1.769354837 | 0.001246675 |
| 101260919 | LOC101260919 | -2.312966404 | 3.13E-07 |
| 101260925 | LOC101260925 | -1.273430789 | 0.006442083 |
| 101260941 | LOC101260941 | -5.771109359 | 5.90E-10 |
| 101261007 | LOC101261007 | -2.079548035 | 1.24E-05 |
| 101261017 | WRKY13 | -3.225560697 | 2.56E-04 |
| 101261026 | LOC101261026 | -1.880257002 | 1.77E-04 |
| 101261030 | LOC101261030 | -3.291459071 | 0.001018277 |
| 101261060 | LOC101261060 | -2.441907113 | 4.62E-16 |
| 101261068 | LOC101261068 | -2.93014993 | 7.72E-06 |
| 101261071 | LOC101261071 | -9.074234019 | 3.53E-12 |
| 101261082 | LOC101261082 | -1.128466553 | 4.07E-04 |
| 101261122 | LOC101261122 | -2.157613511 | 3.19E-05 |
| 101261139 | LOC101261139 | -1.357604293 | 0.002086923 |
| 101261161 | LOC101261161 | -7.077904708 | 8.04E-16 |
| 101261166 | PEPC4 | -1.597345304 | 0.001842714 |
| 101261168 | LOC101261168 | -1.219986454 | 2.50E-04 |
| 101261172 | LOC101261172 | -4.318592553 | 2.36E-10 |
| 101261173 | LOC101261173 | -1.474318871 | 0.003018628 |
| 101261197 | LOC101261197 | -6.724074202 | 2.65E-26 |
| 101261198 | LOC101261198 | -7.894264224 | 4.76E-34 |
| 101261200 | MBF1 | -1.39240192 | 0.001770313 |
| 101261209 | LOC101261209 | -1.597065411 | 0.007673526 |
| 101261216 | LOC101261216 | -1.695297548 | 0.008259183 |
| 101261221 | LOC101261221 | -2.974396147 | 6.95E-04 |
| 101261227 | LOC101261227 | -2.851587553 | 1.97E-04 |
| 101261258 | LOC101261258 | -1.649786272 | 3.83E-04 |
| 101261270 | LOC101261270 | -2.142239627 | 7.48E-05 |
| 101261277 | DRM6 | -1.194169536 | 0.003775065 |
| 101261286 | LOC101261286 | -2.339145452 | 0.002821591 |
| 101261312 | LOC101261312 | -1.930301565 | 4.33E-06 |
| 101261314 | LOC101261314 | -1.970224637 | 4.05E-04 |
| 101261318 | LOC101261318 | -2.078201511 | 0.003918234 |
| 101261334 | LOC101261334 | -2.37942879 | 2.82E-04 |
| 101261339 | LOC101261339 | -2.170457435 | 0.001592929 |
| 101261377 | LOC101261377 | -1.440669449 | 0.001726254 |
| 101261417 | LOC101261417 | -1.60238537 | 0.006114314 |
| 101261419 | LOC101261419 | -2.191964866 | 0.001130477 |
| 101261420 | LOC101261420 | -1.762930087 | 0.001468546 |
| 101261429 | LOC101261429 | -3.210377521 | 1.14E-10 |
| 101261432 | LOC101261432 | -6.789323251 | 1.17E-07 |
| 101261433 | LOC101261433 | -1.303377783 | 0.004275133 |
| 101261442 | LOC101261442 | -1.310268918 | 0.001908636 |
| 101261448 | LOC101261448 | -1.543059815 | 6.28E-04 |
| 101261466 | LOC101261466 | -1.749301052 | 7.44E-04 |
| 101261467 | LOC101261467 | -5.443961306 | 2.00E-16 |
| 101261484 | LOC101261484 | -1.981707646 | 0.003490577 |
| 101261496 | LOC101261496 | -1.81008327 | 9.62E-06 |
| 101261506 | LOC101261506 | -3.510140942 | 5.48E-09 |
| 101261508 | LOC101261508 | -1.618424995 | 9.01E-04 |
| 101261537 | LOC101261537 | -6.164457897 | 4.16E-20 |
| 101261553 | LOC101261553 | -1.381557927 | 0.003363137 |
| 101261555 | LOC101261555 | -1.359021171 | 2.70E-04 |
| 101261593 | LOC101261593 | -4.8351362 | 4.97E-04 |
| 101261603 | LOC101261603 | -3.655405005 | 7.61E-07 |
| 101261625 | LOC101261625 | -4.088488751 | 3.00E-04 |
| 101261631 | LOC101261631 | -4.49751749 | 0.002592845 |
| 101261634 | LOC101261634 | -2.390483514 | 4.56E-05 |
| 101261635 | LOC101261635 | -2.123662087 | 0.001001086 |
| 101261637 | LOC101261637 | -2.0355872 | 3.16E-09 |
| 101261644 | LOC101261644 | -1.697060895 | 8.35E-04 |
| 101261651 | LOC101261651 | -1.721045423 | 0.002165108 |
| 101261689 | LOC101261689 | -2.086697436 | 1.10E-06 |
| 101261693 | ERF-E5 | -1.126408087 | 0.009244445 |
| 101261709 | LOC101261709 | -3.821015908 | 0.002574544 |
| 101261710 | LOC101261710 | -1.432274207 | 0.006407221 |
| 101261712 | ERF-H14 | -3.529608679 | 2.67E-06 |
| 101261729 | LOC101261729 | -2.609687287 | 0.002825775 |
| 101261734 | LOC101261734 | -1.972133105 | 7.85E-04 |
| 101261737 | LOC101261737 | -4.102772619 | 3.52E-20 |
| 101261752 | LOC101261752 | -3.677450194 | 0.005616502 |
| 101261789 | LOC101261789 | -2.573961228 | 1.14E-04 |
| 101261814 | LOC101261814 | -1.559783686 | 0.008557036 |
| 101261826 | LOC101261826 | -3.207448891 | 3.75E-09 |
| 101261831 | LOC101261831 | -3.210965981 | 1.41E-06 |
| 101261868 | LOC101261868 | -1.450861764 | 0.00509595 |
| 101261871 | LOC101261871 | -2.646946991 | 8.64E-05 |
| 101261895 | LOC101261895 | -2.017135598 | 2.33E-11 |
| 101261897 | LOC101261897 | -2.263004703 | 0.002497179 |
| 101261900 | LOC101261900 | -1.52627121 | 0.002668968 |
| 101261901 | LOC101261901 | -1.01127754 | 0.008788296 |
| 101261904 | LOC101261904 | -2.136357192 | 0.001777009 |
| 101261939 | LOC101261939 | -1.315644471 | 2.03E-04 |
| 101261963 | CTOMT1 | -2.842657894 | 5.33E-08 |
| 101261967 | LOC101261967 | -3.11559786 | 0.002892293 |
| 101261986 | LOC101261986 | -3.425954015 | 0.003210717 |
| 101262024 | LOC101262024 | -2.789548451 | 1.83E-08 |
| 101262032 | LOC101262032 | -1.918017823 | 0.001674625 |
| 101262037 | LOC101262037 | -1.309468858 | 5.58E-04 |
| 101262041 | LOC101262041 | -1.778814135 | 3.49E-07 |
| 101262042 | LOC101262042 | -5.472646828 | 4.76E-23 |
| 101262052 | LOC101262052 | -4.097196485 | 2.93E-21 |
| 101262058 | LOC101262058 | -1.465633061 | 4.74E-04 |
| 101262064 | LOC101262064 | -1.38488869 | 0.001307613 |
| 101262082 | LOC101262082 | -1.952895075 | 0.001219426 |
| 101262096 | LOC101262096 | -1.992662374 | 0.008920514 |
| 101262111 | LOC101262111 | -3.580046606 | 4.45E-04 |
| 101262116 | LOC101262116 | -2.655968212 | 5.97E-04 |
| 101262136 | LOC101262136 | -2.318610126 | 0.002106877 |
| 101262164 | LOC101262164 | -3.515991819 | 2.02E-12 |
| 101262174 | LOC101262174 | -3.295757935 | 0.008543209 |
| 101262180 | LOC101262180 | -1.831401738 | 0.001415748 |
| 101262213 | LOC101262213 | -1.315108386 | 7.15E-04 |
| 101262214 | LOC101262214 | -1.961779147 | 7.30E-04 |
| 101262222 | LOC101262222 | -2.0984435 | 7.14E-05 |
| 101262230 | LOC101262230 | -1.738846946 | 0.008750759 |
| 101262241 | LOC101262241 | -1.134549753 | 0.003989192 |
| 101262262 | LOC101262262 | -3.011497883 | 5.36E-27 |
| 101262268 | LOC101262268 | -2.756296045 | 2.70E-12 |
| 101262277 | LOC101262277 | -2.243324499 | 0.002323773 |
| 101262286 | LOC101262286 | -1.351444842 | 2.76E-04 |
| 101262306 | LOC101262306 | -1.429582974 | 0.005368804 |
| 101262334 | LOC101262334 | -3.12429698 | 8.97E-05 |
| 101262335 | LOC101262335 | -2.863515538 | 6.62E-21 |
| 101262339 | LOC101262339 | -3.293462533 | 4.33E-13 |
| 101262344 | LOC101262344 | -1.618185739 | 0.00541646 |
| 101262375 | LOC101262375 | -3.011407464 | 0.001928288 |
| 101262376 | LOC101262376 | -4.360398442 | 1.01E-10 |
| 101262384 | LOC101262384 | -2.259410712 | 0.001642764 |
| 101262408 | LOC101262408 | -2.075694415 | 8.09E-10 |
| 101262415 | LOC101262415 | -1.615248123 | 0.003386826 |
| 101262426 | LOC101262426 | -2.261884262 | 0.002560784 |
| 101262444 | LOC101262444 | -2.183345838 | 0.001959156 |
| 101262477 | LOC101262477 | -2.723871777 | 3.16E-08 |
| 101262483 | LOC101262483 | -3.774016785 | 3.33E-09 |
| 101262509 | LOC101262509 | -4.466430997 | 2.62E-06 |
| 101262511 | LOC101262511 | -1.513291454 | 0.006966021 |
| 101262516 | LOC101262516 | -2.171539206 | 1.65E-07 |
| 101262539 | LOC101262539 | -1.900012777 | 0.004358783 |
| 101262545 | LOC101262545 | -1.394470033 | 0.002716616 |
| 101262546 | SMO1 | -1.612738544 | 0.003344685 |
| 101262550 | LOC101262550 | -1.55681943 | 7.77E-04 |
| 101262578 | LOC101262578 | -1.439245764 | 0.003015345 |
| 101262585 | LOC101262585 | -2.835410803 | 0.007400947 |
| 101262597 | LOC101262597 | -2.971763499 | 3.39E-04 |
| 101262600 | LOC101262600 | -1.409005495 | 3.35E-04 |
| 101262603 | LOC101262603 | -1.389805536 | 0.008185727 |
| 101262609 | LOC101262609 | -1.053068 | 2.07E-04 |
| 101262628 | LOC101262628 | -1.389652549 | 2.71E-04 |
| 101262630 | LOC101262630 | -1.263917474 | 5.56E-04 |
| 101262658 | LOC101262658 | -5.185032619 | 0.001041126 |
| 101262669 | LOC101262669 | -2.50447686 | 2.03E-04 |
| 101262681 | COR413IM1 | -1.371424719 | 0.003205317 |
| 101262737 | LOC101262737 | -2.939900217 | 1.73E-08 |
| 101262756 | LOC101262756 | -6.887647995 | 1.10E-07 |
| 101262762 | LOC101262762 | -6.139773819 | 4.72E-04 |
| 101262783 | LOC101262783 | -1.169659134 | 0.007923406 |
| 101262785 | LOC101262785 | -4.121538809 | 7.67E-11 |
| 101262788 | LOC101262788 | -2.563772667 | 9.20E-04 |
| 101262794 | LOC101262794 | -1.442831734 | 2.30E-05 |
| 101262798 | LOC101262798 | -2.863434338 | 1.05E-04 |
| 101262805 | LOC101262805 | -1.285500659 | 0.001664991 |
| 101262830 | ACAT3 | -1.995840251 | 0.00185578 |
| 101262845 | LOC101262845 | -2.091591485 | 2.93E-04 |
| 101262856 | LOC101262856 | -3.641092171 | 1.21E-06 |
| 101262861 | LOC101262861 | -3.042481686 | 0.001274075 |
| 101262870 | LOC101262870 | -2.045489222 | 0.002169751 |
| 101262874 | LOC101262874 | -1.38291436 | 6.20E-04 |
| 101262883 | LOC101262883 | -1.882478373 | 0.001423042 |
| 101262936 | LOC101262936 | -1.250672525 | 7.91E-04 |
| 101262952 | LOC101262952 | -2.994858132 | 8.40E-14 |
| 101262961 | LOC101262961 | -1.184952386 | 0.002412173 |
| 101262972 | LOC101262972 | -1.663436427 | 0.004522745 |
| 101262993 | LOC101262993 | -1.08461506 | 0.001787199 |
| 101263024 | LOC101263024 | -1.694773579 | 5.14E-04 |
| 101263027 | LOC101263027 | -1.744370404 | 0.006187736 |
| 101263033 | LOC101263033 | -3.197646877 | 2.15E-05 |
| 101263056 | ERF-H4 | -2.612078592 | 0.005691054 |
| 101263060 | LOC101263060 | -2.470476408 | 6.07E-19 |
| 101263062 | LOC101263062 | -1.541090307 | 0.003561691 |
| 101263070 | LOC101263070 | -2.012200197 | 0.00121625 |
| 101263076 | LOC101263076 | -1.705797802 | 2.49E-04 |
| 101263087 | LOC101263087 | -1.155839177 | 0.007218786 |
| 101263088 | LOC101263088 | -2.463825468 | 2.36E-13 |
| 101263097 | LOC101263097 | -3.081787744 | 3.32E-05 |
| 101263123 | LOC101263123 | -3.879595155 | 1.74E-07 |
| 101263160 | LOC101263160 | -1.411779453 | 0.001324789 |
| 101263173 | LOC101263173 | -2.045320965 | 2.19E-08 |
| 101263178 | LOC101263178 | -2.400691205 | 0.003284507 |
| 101263179 | LOC101263179 | -2.484323457 | 2.37E-05 |
| 101263187 | LOC101263187 | -3.379438813 | 5.41E-04 |
| 101263188 | LOC101263188 | -1.965383478 | 6.83E-04 |
| 101263191 | LOC101263191 | -2.656170376 | 2.83E-05 |
| 101263194 | LOC101263194 | -1.738060903 | 0.007204338 |
| 101263216 | LOC101263216 | -1.408451948 | 0.006828499 |
| 101263229 | LOC101263229 | -3.168154608 | 1.81E-06 |
| 101263230 | LOC101263230 | -4.839094675 | 0.001879179 |
| 101263238 | LOC101263238 | -3.270422656 | 1.03E-04 |
| 101263249 | LOC101263249 | -2.13630973 | 1.42E-04 |
| 101263268 | LOC101263268 | -1.804693752 | 3.04E-05 |
| 101263269 | LOC101263269 | -3.691589408 | 4.40E-05 |
| 101263275 | LOC101263275 | -2.596866104 | 1.03E-04 |
| 101263280 | LOC101263280 | -2.544488568 | 7.39E-04 |
| 101263296 | LOC101263296 | -2.008706828 | 0.001855422 |
| 101263304 | LOC101263304 | -4.330058188 | 3.08E-13 |
| 101263327 | LOC101263327 | -1.208666326 | 3.31E-05 |
| 101263337 | LOC101263337 | -1.220894139 | 4.63E-04 |
| 101263369 | LOC101263369 | -2.606620609 | 7.64E-04 |
| 101263386 | LOC101263386 | -2.592679267 | 3.49E-05 |
| 101263397 | LOC101263397 | -1.936125678 | 0.006439579 |
| 101263407 | LOC101263407 | -1.508744528 | 0.001416572 |
| 101263431 | LOC101263431 | -3.977288148 | 5.50E-05 |
| 101263437 | LOC101263437 | -1.280312705 | 0.00144142 |
| 101263456 | LOC101263456 | -1.668667723 | 7.71E-04 |
| 101263458 | LOC101263458 | -1.688698229 | 0.003740079 |
| 101263480 | LOC101263480 | -2.079890334 | 0.003451135 |
| 101263496 | LOC101263496 | -4.399247824 | 3.70E-20 |
| 101263509 | LOC101263509 | -6.469468766 | 6.44E-05 |
| 101263519 | LOC101263519 | -2.166416133 | 0.005989607 |
| 101263532 | LOC101263532 | -2.483979919 | 2.95E-10 |
| 101263533 | LOC101263533 | -1.255633263 | 0.003166953 |
| 101263546 | LOC101263546 | -1.976519241 | 2.38E-09 |
| 101263552 | LOC101263552 | -3.142378542 | 0.002500569 |
| 101263563 | LOC101263563 | -2.102606744 | 5.92E-07 |
| 101263578 | LOC101263578 | -1.242611941 | 0.001347249 |
| 101263579 | LOC101263579 | -2.139631145 | 6.12E-05 |
| 101263591 | LOC101263591 | -1.456898076 | 0.004874737 |
| 101263618 | LOC101263618 | -1.15920115 | 3.16E-04 |
| 101263619 | LOC101263619 | -2.16055656 | 3.97E-07 |
| 101263631 | LOC101263631 | -2.325229457 | 0.002304513 |
| 101263642 | LOC101263642 | -1.345755828 | 1.51E-04 |
| 101263649 | LOC101263649 | -1.41349659 | 0.005875485 |
| 101263656 | LOC101263656 | -3.92447952 | 0.006329747 |
| 101263662 | LOC101263662 | -2.475665425 | 6.74E-04 |
| 101263672 | LOC101263672 | -3.151779218 | 1.46E-05 |
| 101263676 | LOC101263676 | -4.534134472 | 4.17E-15 |
| 101263678 | LOC101263678 | -1.039366103 | 0.003366 |
| 101263682 | LOC101263682 | -2.494171117 | 7.84E-14 |
| 101263705 | LOC101263705 | -1.481437646 | 0.001110186 |
| 101263706 | fen | -1.308666838 | 0.007471078 |
| 101263721 | LOC101263721 | -1.378436039 | 0.004559373 |
| 101263739 | LOC101263739 | -2.804298139 | 4.90E-05 |
| 101263747 | LOC101263747 | -1.275550261 | 5.74E-04 |
| 101263751 | LOC101263751 | -2.058303088 | 1.95E-05 |
| 101263758 | LOC101263758 | -1.516654032 | 0.006739016 |
| 101263759 | LOC101263759 | -1.326330133 | 0.008659562 |
| 101263775 | LOC101263775 | -1.436971528 | 0.007375173 |
| 101263778 | LOC101263778 | -1.236350969 | 0.001572348 |
| 101263802 | LOC101263802 | -2.045179027 | 0.005314101 |
| 101263810 | LOC101263810 | -1.375216008 | 0.003866041 |
| 101263825 | LOC101263825 | -2.217901722 | 9.08E-04 |
| 101263826 | DEFL1 | -2.881213867 | 1.14E-04 |
| 101263833 | LOC101263833 | -1.139678271 | 0.006892051 |
| 101263845 | LOC101263845 | -3.039257855 | 1.45E-04 |
| 101263849 | LOC101263849 | -1.212914265 | 0.002004925 |
| 101263861 | LOC101263861 | -1.79414335 | 1.21E-04 |
| 101263863 | LOC101263863 | -1.001637694 | 8.36E-04 |
| 101263884 | LOC101263884 | -4.188345324 | 7.05E-06 |
| 101263895 | LOC101263895 | -3.533211475 | 6.29E-06 |
| 101263897 | LOC101263897 | -1.88362927 | 0.006320559 |
| 101263901 | LOC101263901 | -1.462940402 | 0.002696579 |
| 101263903 | LOC101263903 | -2.654787205 | 3.15E-05 |
| 101263911 | LOC101263911 | -1.766559539 | 2.59E-04 |
| 101263915 | LOC101263915 | -2.127815864 | 6.95E-07 |
| 101263925 | LOC101263925 | -1.313608996 | 6.83E-06 |
| 101263940 | LOC101263940 | -1.827584845 | 0.006936228 |
| 101263963 | LOC101263963 | -1.155830664 | 0.004130162 |
| 101264068 | LOC101264068 | -1.298794085 | 6.65E-04 |
| 101264069 | LOC101264069 | -2.894916997 | 4.36E-04 |
| 101264088 | LOC101264088 | -2.371666429 | 4.64E-06 |
| 101264096 | LOC101264096 | -2.519420699 | 5.86E-07 |
| 101264133 | LOC101264133 | -6.563029624 | 3.24E-04 |
| 101264140 | LOC101264140 | -1.444631995 | 0.003005153 |
| 101264172 | LOC101264172 | -2.669886321 | 7.19E-04 |
| 101264202 | LOC101264202 | -2.561233384 | 2.64E-05 |
| 101264234 | LOC101264234 | -1.004817252 | 0.007914477 |
| 101264282 | LOC101264282 | -1.797955561 | 0.009233576 |
| 101264288 | LOC101264288 | -4.264375214 | 0.002760924 |
| 101264294 | LOC101264294 | -1.168946253 | 0.003817063 |
| 101264309 | LOC101264309 | -1.534495368 | 0.007153926 |
| 101264329 | LOC101264329 | -1.194927948 | 4.16E-04 |
| 101264357 | LOC101264357 | -3.629006626 | 2.65E-18 |
| 101264365 | LOC101264365 | -8.044465664 | 1.93E-08 |
| 101264367 | LOC101264367 | -2.34242595 | 0.002082958 |
| 101264370 | LOC101264370 | -2.233323144 | 0.001103238 |
| 101264401 | LOC101264401 | -1.564601661 | 7.65E-04 |
| 101264404 | LOC101264404 | -1.649421234 | 0.00340584 |
| 101264428 | LOC101264428 | -5.241585668 | 2.21E-14 |
| 101264429 | LOC101264429 | -1.375757978 | 0.005499743 |
| 101264434 | LOC101264434 | -1.044115174 | 0.004322707 |
| 101264436 | LOC101264436 | -2.082564795 | 1.89E-04 |
| 101264462 | LOC101264462 | -4.745585331 | 0.004245547 |
| 101264472 | LOC101264472 | -2.777391686 | 0.00141434 |
| 101264524 | LOC101264524 | -4.488147886 | 5.13E-05 |
| 101264539 | LOC101264539 | -2.449270643 | 1.46E-06 |
| 101264544 | LOC101264544 | -1.336559544 | 0.002098013 |
| 101264556 | TRM9 | -4.592571337 | 4.20E-06 |
| 101264559 | LOC101264559 | -2.56397948 | 4.60E-07 |
| 101264564 | LOC101264564 | -1.054518962 | 4.41E-04 |
| 101264583 | LOC101264583 | -1.599918161 | 3.57E-05 |
| 101264586 | LOC101264586 | -2.321168176 | 0.001799456 |
| 101264591 | LOC101264591 | -2.845282169 | 4.45E-04 |
| 101264597 | LOC101264597 | -2.972724429 | 3.12E-10 |
| 101264612 | LOC101264612 | -3.374089508 | 1.10E-05 |
| 101264616 | LOC101264616 | -1.965552082 | 1.48E-04 |
| 101264620 | LOC101264620 | -4.894223039 | 2.89E-08 |
| 101264622 | LOC101264622 | -2.231306212 | 6.09E-04 |
| 101264624 | LOC101264624 | -1.20966316 | 0.001189006 |
| 101264638 | LOC101264638 | -1.699646774 | 8.70E-04 |
| 101264659 | LOC101264659 | -2.212886154 | 0.007387027 |
| 101264672 | LOC101264672 | -1.257945895 | 3.11E-04 |
| 101264683 | LOC101264683 | -1.81351116 | 0.009314067 |
| 101264707 | LOC101264707 | -1.959026906 | 3.39E-05 |
| 101264726 | THIC | -5.286805552 | 2.19E-22 |
| 101264734 | LOC101264734 | -1.51861499 | 0.006587605 |
| 101264759 | LOC101264759 | -1.105302107 | 0.002170858 |
| 101264768 | LOC101264768 | -1.941135188 | 0.005069315 |
| 101264779 | LOC101264779 | -2.39017345 | 8.32E-04 |
| 101264780 | LOC101264780 | -1.883464758 | 8.45E-06 |
| 101264789 | LOC101264789 | -2.068785399 | 0.00197059 |
| 101264790 | LOC101264790 | -1.509905252 | 0.001413678 |
| 101264814 | LOC101264814 | -3.980328154 | 5.73E-25 |
| 101264838 | LOC101264838 | -2.274509029 | 1.42E-04 |
| 101264862 | LOC101264862 | -1.800979474 | 0.004688605 |
| 101264882 | LOC101264882 | -1.787980903 | 0.005354198 |
| 101264893 | LOC101264893 | -2.291964969 | 3.25E-07 |
| 101264899 | LOC101264899 | -1.314213221 | 0.004374069 |
| 101264919 | LOC101264919 | -7.271786621 | 4.56E-05 |
| 101264923 | LOC101264923 | -1.85523906 | 0.005990029 |
| 101264924 | LOC101264924 | -1.260935447 | 4.34E-04 |
| 101264936 | Hsp20.0 | -2.360772484 | 3.89E-04 |
| 101264937 | ABCG60 | -5.281366277 | 0.004381406 |
| 101264972 | LOC101264972 | -2.326839707 | 1.39E-05 |
| 101264975 | LOC101264975 | -3.189781499 | 2.72E-04 |
| 101264980 | LOC101264980 | -1.601063273 | 0.001345125 |
| 101264985 | LOC101264985 | -2.763307994 | 2.63E-05 |
| 101264987 | LOC101264987 | -2.96038852 | 2.63E-05 |
| 101265016 | LOC101265016 | -1.287094581 | 0.008986898 |
| 101265021 | LOC101265021 | -4.491223956 | 2.26E-09 |
| 101265030 | LOC101265030 | -2.060104244 | 1.06E-05 |
| 101265056 | CMT3 | -3.468835032 | 2.03E-06 |
| 101265059 | LOC101265059 | -1.092345772 | 0.002308538 |
| 101265089 | LOC101265089 | -2.410214095 | 1.16E-07 |
| 101265092 | LOC101265092 | -2.300173761 | 6.07E-12 |
| 101265093 | LOC101265093 | -2.602872031 | 1.93E-04 |
| 101265128 | LOC101265128 | -1.262067844 | 0.001029764 |
| 101265143 | LOC101265143 | -1.995183511 | 2.17E-04 |
| 101265154 | LOC101265154 | -2.549683677 | 1.78E-05 |
| 101265169 | LOC101265169 | -3.002380981 | 0.003338988 |
| 101265170 | LOC101265170 | -3.86163439 | 1.43E-14 |
| 101265206 | LOC101265206 | -1.412920421 | 0.001412365 |
| 101265231 | LOC101265231 | -2.071928147 | 4.05E-06 |
| 101265232 | FKBP18 | -1.292306057 | 4.22E-05 |
| 101265234 | LOC101265234 | -4.161096772 | 1.65E-08 |
| 101265239 | LOC101265239 | -1.97239059 | 2.60E-05 |
| 101265242 | LOC101265242 | -2.53472046 | 1.93E-04 |
| 101265254 | LOC101265254 | -1.316113872 | 0.003122581 |
| 101265280 | LOC101265280 | -1.928796378 | 7.77E-06 |
| 101265322 | LOC101265322 | -3.041843492 | 2.39E-17 |
| 101265323 | LOC101265323 | -1.270924504 | 0.002899894 |
| 101265327 | LOC101265327 | -1.385371302 | 6.78E-06 |
| 101265339 | LOC101265339 | -1.793804436 | 3.61E-04 |
| 101265346 | LOC101265346 | -1.686969746 | 6.85E-05 |
| 101265355 | LOL1 | -2.587040741 | 2.32E-07 |
| 101265371 | LOC101265371 | -2.679916775 | 4.97E-06 |
| 101265380 | LOC101265380 | -6.530054266 | 1.45E-36 |
| 101265401 | LOC101265401 | -2.808302825 | 9.71E-04 |
| 101265405 | LOC101265405 | -6.027354684 | 2.76E-08 |
| 101265431 | LOC101265431 | -2.11599523 | 5.31E-04 |
| 101265439 | LOC101265439 | -1.347458103 | 0.005453272 |
| 101265448 | LOC101265448 | -3.961973065 | 7.70E-09 |
| 101265455 | LOC101265455 | -3.277490225 | 7.25E-05 |
| 101265487 | LOC101265487 | -4.208236733 | 2.33E-14 |
| 101265521 | LOC101265521 | -1.296632117 | 0.001582901 |
| 101265534 | LOC101265534 | -1.302468248 | 2.45E-04 |
| 101265537 | LOC101265537 | -2.432718737 | 0.001660338 |
| 101265549 | LOC101265549 | -1.717701712 | 8.49E-04 |
| 101265579 | LOC101265579 | -2.080393605 | 1.13E-04 |
| 101265590 | LOC101265590 | -3.083961716 | 1.97E-07 |
| 101265619 | LOC101265619 | -3.35986946 | 5.49E-04 |
| 101265673 | LOC101265673 | -2.755166434 | 3.35E-13 |
| 101265679 | LOC101265679 | -1.821028179 | 0.002236189 |
| 101265691 | LOC101265691 | -3.476354932 | 2.76E-05 |
| 101265706 | LOC101265706 | -1.498004912 | 0.001002696 |
| 101265718 | LOC101265718 | -2.150658766 | 0.001379828 |
| 101265731 | LOC101265731 | -2.849528168 | 0.001931671 |
| 101265749 | LOC101265749 | -2.796130061 | 1.08E-04 |
| 101265765 | TRM6/7/8a | -2.773597635 | 5.39E-05 |
| 101265780 | GRP1 | -2.988200211 | 1.21E-16 |
| 101265786 | LOC101265786 | -1.520507246 | 0.006865872 |
| 101265808 | LOC101265808 | -2.899024033 | 0.001661557 |
| 101265837 | LOC101265837 | -2.151212566 | 7.93E-04 |
| 101265854 | LOC101265854 | -2.376500119 | 1.94E-06 |
| 101265862 | LOC101265862 | -1.632537419 | 4.32E-07 |
| 101265864 | LOC101265864 | -1.121920236 | 0.00187187 |
| 101265867 | LOC101265867 | -2.415900521 | 3.82E-04 |
| 101265881 | LOC101265881 | -4.491748615 | 3.67E-10 |
| 101265893 | LOC101265893 | -3.514125878 | 1.05E-11 |
| 101265921 | LOC101265921 | -3.069692407 | 9.27E-07 |
| 101265935 | LOC101265935 | -1.435713054 | 0.004643646 |
| 101265937 | LOC101265937 | -5.380976473 | 1.76E-04 |
| 101265950 | LOC101265950 | -1.167837141 | 1.21E-04 |
| 101265951 | LOC101265951 | -2.596509367 | 6.64E-05 |
| 101265972 | LOC101265972 | -2.99845262 | 3.84E-06 |
| 101265982 | LOC101265982 | -1.532448074 | 5.14E-04 |
| 101265984 | TRM22 | -1.33568944 | 9.87E-05 |
| 101265988 | LOC101265988 | -1.082614124 | 0.004918234 |
| 101265993 | LOC101265993 | -1.720661675 | 0.002384261 |
| 101265996 | LOC101265996 | -2.070799587 | 7.20E-05 |
| 101266025 | LOC101266025 | -3.15737858 | 3.39E-05 |
| 101266037 | LOC101266037 | -1.536422517 | 6.80E-04 |
| 101266043 | LOC101266043 | -3.173553865 | 3.91E-05 |
| 101266047 | LOC101266047 | -2.698101673 | 0.00323996 |
| 101266083 | LOC101266083 | -1.450088301 | 0.003319117 |
| 101266105 | LOC101266105 | -1.83290622 | 0.006919818 |
| 101266110 | LOC101266110 | -3.370202815 | 1.21E-06 |
| 101266112 | LOC101266112 | -3.067697294 | 3.70E-07 |
| 101266118 | TMT2 | -1.805626167 | 1.33E-05 |
| 101266139 | LOC101266139 | -1.177824148 | 0.001048231 |
| 101266146 | LOC101266146 | -1.19461111 | 0.002373798 |
| 101266183 | LOC101266183 | -1.441194861 | 0.00116383 |
| 101266186 | LOC101266186 | -3.206650425 | 2.69E-05 |
| 101266207 | LOC101266207 | -2.695971049 | 6.35E-04 |
| 101266240 | LOC101266240 | -3.241373926 | 8.00E-07 |
| 101266265 | LOC101266265 | -3.047616762 | 4.39E-13 |
| 101266300 | LOC101266300 | -2.604235675 | 1.26E-06 |
| 101266314 | LOC101266314 | -1.350020798 | 0.005443627 |
| 101266336 | LOC101266336 | -1.080646476 | 4.41E-04 |
| 101266372 | LOC101266372 | -1.355152541 | 0.001148071 |
| 101266376 | LOC101266376 | -2.285308687 | 1.09E-05 |
| 101266402 | LOC101266402 | -1.006157641 | 4.15E-04 |
| 101266406 | LOC101266406 | -1.672877877 | 1.24E-05 |
| 101266408 | LOC101266408 | -2.511539747 | 2.14E-05 |
| 101266414 | LOC101266414 | -1.202888621 | 0.004754142 |
| 101266437 | LOC101266437 | -2.282367954 | 1.99E-05 |
| 101266455 | LOC101266455 | -2.9085807 | 7.46E-05 |
| 101266475 | LOC101266475 | -1.453952574 | 0.004543538 |
| 101266483 | LOC101266483 | -1.863091448 | 3.81E-06 |
| 101266486 | LOC101266486 | -1.878990873 | 8.18E-07 |
| 101266487 | LOC101266487 | -1.966184356 | 2.65E-07 |
| 101266500 | LOC101266500 | -3.220710935 | 0.001279903 |
| 101266530 | LOC101266530 | -2.964825449 | 0.00248598 |
| 101266544 | ABCC7 | -2.329564399 | 2.56E-08 |
| 101266546 | TRM10/11 | -3.900714482 | 3.31E-07 |
| 101266605 | LOC101266605 | -2.704302487 | 4.67E-04 |
| 101266628 | LOC101266628 | -1.770092249 | 1.72E-04 |
| 101266644 | LOC101266644 | -1.433942155 | 1.98E-05 |
| 101266648 | LOC101266648 | -2.626758488 | 0.009286852 |
| 101266650 | LOC101266650 | -2.906738852 | 2.50E-15 |
| 101266674 | LOC101266674 | -1.902025932 | 9.81E-05 |
| 101266709 | LOC101266709 | -1.271577608 | 0.002860165 |
| 101266764 | LOC101266764 | -2.085459925 | 0.002470769 |
| 101266778 | LOC101266778 | -4.115484286 | 1.26E-07 |
| 101266779 | LOC101266779 | -1.557443577 | 0.002666976 |
| 101266782 | LOC101266782 | -1.266623335 | 0.003880454 |
| 101266789 | LOC101266789 | -3.497609578 | 2.68E-11 |
| 101266818 | LOC101266818 | -3.423542932 | 2.34E-09 |
| 101266842 | LOC101266842 | -2.412089711 | 1.20E-04 |
| 101266870 | LOC101266870 | -1.809917641 | 6.57E-05 |
| 101266875 | LOC101266875 | -2.815792132 | 2.41E-14 |
| 101266890 | LOC101266890 | -2.212259208 | 0.006924575 |
| 101266902 | AOS2 | -2.047710383 | 0.002298139 |
| 101266906 | LOC101266906 | -1.052847052 | 8.23E-05 |
| 101266928 | LOC101266928 | -2.290969081 | 4.64E-05 |
| 101266944 | LOC101266944 | -1.404652953 | 4.48E-07 |
| 101266952 | LOC101266952 | -1.35630031 | 0.005511679 |
| 101266964 | LOC101266964 | -2.512797261 | 2.99E-05 |
| 101266976 | LOC101266976 | -3.480906619 | 3.00E-10 |
| 101266980 | LOC101266980 | -1.693072322 | 5.20E-05 |
| 101266997 | TRM25 | -2.791153026 | 3.56E-05 |
| 101267024 | LOC101267024 | -1.819913285 | 1.19E-06 |
| 101267033 | LOC101267033 | -5.037585744 | 7.34E-23 |
| 101267037 | LOC101267037 | -1.814384286 | 0.00914436 |
| 101267047 | LOC101267047 | -1.998652161 | 0.001232599 |
| 101267050 | LOC101267050 | -5.889707011 | 3.80E-04 |
| 101267064 | LOC101267064 | -3.7732235 | 1.04E-09 |
| 101267067 | LOC101267067 | -1.930245325 | 8.95E-04 |
| 101267071 | LOC101267071 | -2.386664671 | 0.001679126 |
| 101267098 | GPXle-2 | -2.306536631 | 5.80E-08 |
| 101267100 | LOC101267100 | -2.118390176 | 3.62E-04 |
| 101267113 | LOC101267113 | -1.426517636 | 0.003045102 |
| 101267135 | LOC101267135 | -2.589056063 | 2.66E-05 |
| 101267136 | LOC101267136 | -1.451647387 | 7.49E-04 |
| 101267140 | LOC101267140 | -2.575627535 | 8.41E-04 |
| 101267142 | LOC101267142 | -2.537234474 | 1.50E-05 |
| 101267157 | LOC101267157 | -2.413931063 | 0.002835306 |
| 101267191 | LOC101267191 | -5.692478411 | 1.50E-05 |
| 101267194 | LOC101267194 | -1.491871373 | 5.94E-04 |
| 101267201 | LOC101267201 | -2.484479168 | 4.56E-05 |
| 101267247 | LOC101267247 | -1.518358855 | 0.004946852 |
| 101267260 | LOC101267260 | -1.999609481 | 0.004847379 |
| 101267266 | LOC101267266 | -1.006867693 | 0.004895834 |
| 101267286 | LOC101267286 | -2.096152311 | 0.00426472 |
| 101267287 | LOC101267287 | -1.611228299 | 1.15E-06 |
| 101267307 | LOC101267307 | -2.125276313 | 0.001455684 |
| 101267323 | LOC101267323 | -1.970724995 | 2.56E-04 |
| 101267344 | LOC101267344 | -1.508444947 | 0.003763464 |
| 101267350 | LOC101267350 | -2.413760949 | 7.01E-04 |
| 101267367 | LOC101267367 | -3.40592696 | 1.06E-06 |
| 101267404 | LOC101267404 | -4.261475538 | 4.01E-12 |
| 101267430 | LOC101267430 | -1.450796772 | 0.005696447 |
| 101267452 | LOC101267452 | -1.131264437 | 0.006390779 |
| 101267460 | LOC101267460 | -1.181596865 | 0.008399545 |
| 101267470 | LOC101267470 | -1.47106755 | 5.09E-04 |
| 101267471 | LOC101267471 | -2.275791002 | 0.001832484 |
| 101267480 | LOC101267480 | -4.708983062 | 6.01E-23 |
| 101267486 | LOC101267486 | -2.066234337 | 1.59E-07 |
| 101267488 | LOC101267488 | -3.236466711 | 1.67E-04 |
| 101267503 | LOC101267503 | -1.851124577 | 3.99E-05 |
| 101267512 | LOC101267512 | -4.068215473 | 1.57E-04 |
| 101267517 | LOC101267517 | -8.097469314 | 7.62E-06 |
| 101267520 | LOC101267520 | -3.046520443 | 5.52E-06 |
| 101267522 | LOC101267522 | -1.720038669 | 0.002003354 |
| 101267527 | LOC101267527 | -1.78369934 | 9.33E-06 |
| 101267575 | LOC101267575 | -3.113150152 | 0.006647067 |
| 101267609 | LOC101267609 | -1.25669694 | 0.005363052 |
| 101267611 | LOC101267611 | -1.274012695 | 2.59E-04 |
| 101267613 | LOC101267613 | -1.204575247 | 0.003447307 |
| 101267629 | LOC101267629 | -1.730218475 | 9.87E-08 |
| 101267644 | LOC101267644 | -1.024114731 | 5.25E-04 |
| 101267647 | LOC101267647 | -1.684290452 | 7.09E-04 |
| 101267663 | LOC101267663 | -1.884762568 | 8.65E-06 |
| 101267671 | LOC101267671 | -2.367111009 | 2.27E-13 |
| 101267673 | LOC101267673 | -5.047500272 | 3.90E-36 |
| 101267675 | LOC101267675 | -2.283561187 | 2.07E-10 |
| 101267677 | LOC101267677 | -1.379375908 | 3.09E-04 |
| 101267681 | LOC101267681 | -2.963262747 | 2.01E-04 |
| 101267686 | LOC101267686 | -2.333169794 | 5.71E-08 |
| 101267691 | LOC101267691 | -2.026065764 | 9.20E-05 |
| 101267705 | LOC101267705 | -1.566296388 | 1.64E-04 |
| 101267720 | LOC101267720 | -2.693237465 | 0.003974421 |
| 101267742 | LOC101267742 | -2.591756058 | 6.24E-04 |
| 101267781 | LOC101267781 | -2.272803642 | 1.34E-06 |
| 101267784 | LOC101267784 | -2.326568034 | 2.35E-10 |
| 101267788 | LOC101267788 | -1.396989639 | 7.06E-04 |
| 101267790 | LOC101267790 | -1.124953523 | 0.001480108 |
| 101267799 | LOC101267799 | -2.129950802 | 4.33E-04 |
| 101267811 | LOC101267811 | -1.041826467 | 0.00351175 |
| 101267831 | CYP706C2 | -1.337251409 | 0.003104613 |
| 101267838 | LOC101267838 | -2.600672033 | 5.23E-05 |
| 101267854 | LOC101267854 | -3.83899699 | 1.99E-05 |
| 101267861 | LOC101267861 | -2.442593432 | 1.68E-05 |
| 101267862 | LOC101267862 | -1.039177881 | 0.004089798 |
| 101267869 | LOC101267869 | -1.159532487 | 0.002671381 |
| 101267894 | LOC101267894 | -1.01016115 | 0.002220399 |
| 101267898 | LOC101267898 | -4.036022379 | 1.14E-09 |
| 101267900 | LOC101267900 | -1.497475368 | 0.006966341 |
| 101267934 | LOC101267934 | -2.844647447 | 9.35E-05 |
| 101267973 | LOC101267973 | -3.606935279 | 9.83E-04 |
| 101267988 | LOC101267988 | -1.734815323 | 7.54E-05 |
| 101268000 | LOC101268000 | -1.254222136 | 0.007363847 |
| 101268012 | LOC101268012 | -2.12622006 | 6.94E-04 |
| 101268017 | LOC101268017 | -1.999051216 | 1.11E-07 |
| 101268051 | LOC101268051 | -1.655119686 | 3.29E-04 |
| 101268055 | LOC101268055 | -2.188346483 | 1.16E-04 |
| 101268070 | LOC101268070 | -3.177864176 | 2.33E-06 |
| 101268115 | LOC101268115 | -2.401441661 | 0.003124106 |
| 101268142 | LOC101268142 | -1.314946822 | 0.001917022 |
| 101268154 | LOC101268154 | -3.658076886 | 3.59E-06 |
| 101268176 | LOC101268176 | -1.771469028 | 9.12E-05 |
| 101268192 | LOC101268192 | -2.24301598 | 0.002496177 |
| 101268204 | LOC101268204 | -1.615564583 | 0.002160044 |
| 101268210 | LOC101268210 | -3.22134172 | 0.005395219 |
| 101268223 | LOC101268223 | -1.439795423 | 0.005941067 |
| 101268225 | LOC101268225 | -1.484077954 | 0.006569541 |
| 101268227 | LOC101268227 | -2.873970916 | 1.86E-06 |
| 101268231 | LOC101268231 | -1.905609319 | 0.001306697 |
| 101268238 | LOC101268238 | -3.449963547 | 2.51E-11 |
| 101268246 | LOC101268246 | -2.158213849 | 0.001223147 |
| 101268247 | LOC101268247 | -3.354522241 | 2.09E-08 |
| 101268249 | LOC101268249 | -1.378347726 | 1.51E-05 |
| 101268251 | LOC101268251 | -1.687090781 | 0.009118617 |
| 101268259 | LOC101268259 | -1.743907805 | 0.00380003 |
| 101268314 | LOC101268314 | -5.603382371 | 1.11E-51 |
| 101268331 | LOC101268331 | -1.913908492 | 2.09E-04 |
| 101268335 | LOC101268335 | -1.873769971 | 0.00570739 |
| 101268348 | LOC101268348 | -1.927653533 | 8.76E-05 |
| 101268370 | LOC101268370 | -2.584947988 | 2.80E-04 |
| 101268375 | LOC101268375 | -3.427924682 | 2.25E-20 |
| 101268407 | LOC101268407 | -1.691138115 | 0.001560352 |
| 101268410 | LOC101268410 | -3.266818967 | 1.02E-09 |
| 101268420 | LOC101268420 | -2.186715726 | 3.86E-05 |
| 101268475 | LOC101268475 | -1.598169483 | 2.98E-04 |
| 101268479 | LOC101268479 | -1.797440859 | 1.89E-04 |
| 101268496 | LOC101268496 | -1.366142427 | 3.20E-05 |
| 101268514 | LOC101268514 | -2.912420469 | 4.13E-05 |
| 101268530 | LOC101268530 | -2.64691125 | 1.12E-10 |
| 101268547 | LOC101268547 | -2.494511841 | 2.56E-06 |
| 101268549 | ABCB18 | -2.104184719 | 0.003834815 |
| 101268555 | LOC101268555 | -4.021511467 | 1.23E-07 |
| 101268556 | LOC101268556 | -2.892048507 | 8.55E-10 |
| 101268576 | LOC101268576 | -3.61970271 | 2.52E-12 |
| 101268615 | LOC101268615 | -3.887096335 | 3.89E-18 |
| 101268619 | LOC101268619 | -6.276252404 | 6.63E-08 |
| 101268630 | LOC101268630 | -2.836419992 | 3.54E-05 |
| 101268631 | LOC101268631 | -1.841626983 | 0.007430911 |
| 101268639 | LOC101268639 | -3.107381705 | 2.30E-04 |
| 101268680 | LOC101268680 | -1.880733254 | 0.002256685 |
| 101268700 | LOC101268700 | -2.26172161 | 3.03E-06 |
| 101268708 | LOC101268708 | -1.182839328 | 0.002984062 |
| 101268710 | LOC101268710 | -1.358773907 | 0.006223877 |
| 101268722 | LOC101268722 | -1.169326394 | 0.004495861 |
| 101268731 | LOC101268731 | -2.35491167 | 1.87E-04 |
| 101268742 | LOC101268742 | -1.412749783 | 0.004601595 |
| 101268772 | LOC101268772 | -1.711787099 | 0.00137602 |
| 101268800 | LOC101268800 | -1.607511634 | 3.01E-07 |
| 101268810 | LOC101268810 | -1.00206652 | 0.008004589 |
| 101268821 | LOC101268821 | -2.18500345 | 0.001037051 |
| 101268824 | LOC101268824 | -1.305028299 | 1.86E-05 |
| 101268839 | LOC101268839 | -2.27179843 | 0.004058367 |
| 101268851 | LOC101268851 | -1.653536348 | 0.001396969 |
| 101268864 | LOC101268864 | -2.457405693 | 6.96E-04 |
| 104644379 | LOC104644379 | -1.208288961 | 0.004569572 |
| 104644567 | LOC104644567 | -3.244603118 | 2.63E-04 |
| 104644778 | LOC104644778 | -1.930539913 | 0.003948217 |
| 104645197 | LOC104645197 | -2.048317941 | 0.006480944 |
| 104645201 | LOC104645201 | -2.191247004 | 0.006290755 |
| 104645217 | LOC104645217 | -1.524333386 | 7.17E-05 |
| 104645225 | LOC104645225 | -5.337989437 | 0.005337947 |
| 104645714 | LOC104645714 | -4.602176697 | 4.65E-05 |
| 104645730 | LOC104645730 | -2.287169838 | 0.002320238 |
| 104645845 | LOC104645845 | -3.051507747 | 0.002186918 |
| 104645850 | LOC104645850 | -4.074332491 | 1.63E-04 |
| 104645871 | LOC104645871 | -1.62524343 | 9.00E-05 |
| 104645888 | LOC104645888 | -3.958027906 | 1.93E-10 |
| 104645908 | LOC104645908 | -2.718807676 | 2.50E-04 |
| 104646014 | LOC104646014 | -3.645431566 | 2.87E-04 |
| 104646036 | LOC104646036 | -3.385744364 | 3.90E-09 |
| 104646307 | LOC104646307 | -2.674540889 | 0.001039534 |
| 104646528 | LOC104646528 | -1.362343687 | 0.006940606 |
| 104646538 | OFP8 | -1.12633647 | 0.00548122 |
| 104646570 | LOC104646570 | -1.970688774 | 5.01E-04 |
| 104646595 | LOC104646595 | -2.186628518 | 4.89E-05 |
| 104646606 | LOC104646606 | -1.555812564 | 0.002378901 |
| 104646731 | LOC104646731 | -1.64591055 | 8.01E-06 |
| 104647252 | LOC104647252 | -2.442167592 | 1.74E-04 |
| 104647295 | LOC104647295 | -2.922540761 | 6.43E-04 |
| 104647560 | LOC104647560 | -1.696556063 | 0.004314638 |
| 104647672 | LOC104647672 | -1.995255033 | 4.52E-06 |
| 104647762 | LOC104647762 | -1.780201645 | 2.11E-04 |
| 104647773 | LOC104647773 | -5.156344898 | 0.005986301 |
| 104647802 | LOC104647802 | -2.925799745 | 3.55E-04 |
| 104647982 | LOC104647982 | -3.028065801 | 0.005707309 |
| 104648009 | LOC104648009 | -5.36782261 | 1.36E-08 |
| 104648246 | LOC104648246 | -2.381378096 | 6.20E-04 |
| 104648268 | LOC104648268 | -5.046677692 | 1.32E-05 |
| 104648378 | LOC104648378 | -3.252235939 | 9.32E-04 |
| 104648415 | LOC104648415 | -1.907733438 | 0.002026782 |
| 104648418 | LOC104648418 | -2.325449423 | 5.20E-04 |
| 104648447 | LOC104648447 | -3.189079429 | 0.009079782 |
| 104648477 | LOC104648477 | -1.965393315 | 0.002471562 |
| 104648481 | LOC104648481 | -5.873673418 | 0.001067359 |
| 104648487 | LOC104648487 | -4.0496924 | 1.19E-09 |
| 104648542 | LOC104648542 | -1.969113432 | 0.002474479 |
| 104648543 | LOC104648543 | -1.78880239 | 0.0034429 |
| 104648544 | LOC104648544 | -1.998404465 | 0.002183204 |
| 104648550 | LOC104648550 | -6.119375538 | 9.85E-08 |
| 104648556 | LOC104648556 | -2.779713185 | 4.89E-12 |
| 104648557 | LOC104648557 | -1.431618512 | 1.48E-05 |
| 104648772 | LOC104648772 | -2.462515551 | 0.004563274 |
| 104648816 | LOC104648816 | -2.155250738 | 7.94E-06 |
| 104648958 | LOC104648958 | -3.115413795 | 0.003366538 |
| 104648962 | LOC104648962 | -1.727611992 | 0.007160511 |
| 104649154 | LOC104649154 | -2.2499154 | 1.54E-06 |
| 104649361 | LOC104649361 | -1.306326311 | 4.11E-04 |
| 104649465 | LOC104649465 | -2.596659552 | 0.005256763 |
| 104649487 | LOC104649487 | -1.332609613 | 0.006143712 |
| 104649608 | LOC104649608 | -3.538674558 | 3.64E-06 |
| 107196795 | LOC107196795 | -1.626963593 | 0.003008293 |
| 108348021 | sbt4c | -2.480307044 | 4.18E-09 |
| 109118724 | LOC109118724 | -1.889181319 | 0.002752598 |
| 109119452 | LOC109119452 | -4.320200024 | 0.007232115 |
| 109119998 | LOC109119998 | -1.922169254 | 0.001745949 |
| 109120152 | LOC109120152 | -2.534055563 | 0.005712403 |
| 109120363 | LOC109120363 | -1.862760444 | 2.77E-04 |
| 109120423 | LOC109120423 | -2.375585549 | 2.12E-04 |
| 109120649 | LOC109120649 | -5.801633751 | 7.50E-04 |
| 109121165 | LOC109121165 | -5.7396618 | 7.45E-05 |
| 112940010 | LOC112940010 | -2.04613968 | 1.14E-05 |
| 112941536 | LOC112941536 | -3.322614241 | 3.89E-12 |
| 112941556 | LOC112941556 | -1.926112462 | 4.35E-05 |
| 112941717 | LOC112941717 | -1.855703507 | 0.005142309 |
| 543527 | LEJA1 | -1.271860464 | 0.005045932 |
| 543532 | ProT3 | -2.289348448 | 2.60E-05 |
| 543544 | IAA17 | -3.019985816 | 6.02E-05 |
| 543549 | SSTLE1 | -3.42591527 | 7.22E-09 |
| 543556 | EXPA3 | -1.258354408 | 4.50E-04 |
| 543594 | Rem-2 | -2.507666787 | 2.23E-07 |
| 543610 | TBG7 | -2.391532859 | 4.97E-05 |
| 543612 | TBG6 | -2.130715184 | 0.004062037 |
| 543626 | XET4 | -2.047537267 | 0.003121777 |
| 543630 | FALSIFLORA | -5.029269538 | 2.56E-08 |
| 543640 | Aqp2 | -1.457158775 | 3.38E-04 |
| 543641 | LOC543641 | -1.973737379 | 0.001978081 |
| 543644 | TKR | -3.472179071 | 2.98E-05 |
| 543649 | NSY | -2.408071514 | 0.006714163 |
| 543650 | AO1 | -2.30330569 | 0.002241478 |
| 543656 | LOC543656 | -1.693079184 | 0.001192743 |
| 543670 | PTOX | -2.894438072 | 4.61E-05 |
| 543694 | LOC543694 | -2.040385239 | 1.02E-04 |
| 543698 | LOC543698 | -2.876557098 | 1.66E-07 |
| 543699 | LOC543699 | -1.119415859 | 0.001598421 |
| 543702 | HMGR | -2.341586999 | 3.78E-06 |
| 543711 | DREB1 | -2.224622595 | 0.002639583 |
| 543715 | LOC543715 | -2.921043344 | 7.31E-04 |
| 543722 | Cycd3c2 | -3.952910532 | 6.91E-09 |
| 543724 | Exp18 | -3.15162658 | 1.55E-18 |
| 543730 | LOC543730 | -3.846231373 | 9.17E-05 |
| 543738 | Ht3 | -1.070099089 | 0.00379921 |
| 543741 | TGAS118 | -6.472576732 | 1.52E-04 |
| 543744 | CycA1 | -2.384500708 | 5.83E-05 |
| 543747 | CycB2 | -3.238190494 | 1.36E-04 |
| 543748 | CycD3 | -1.721418164 | 1.03E-04 |
| 543756 | Gts1 | -1.726802277 | 1.27E-08 |
| 543760 | Yfe37 | -4.666942567 | 8.87E-48 |
| 543762 | Opr2 | -2.770399273 | 8.73E-07 |
| 543765 | St3 | -1.669476604 | 0.001198401 |
| 543766 | KdsA | -1.419572081 | 6.42E-04 |
| 543768 | CdkB1 | -2.347216524 | 1.02E-04 |
| 543769 | CdkB2 | -3.032440537 | 1.51E-04 |
| 543778 | Ptpkis1 | -1.807716276 | 5.07E-07 |
| 543779 | Hxk1 | -2.980597155 | 3.06E-09 |
| 543801 | Det2 | -3.268796674 | 1.61E-14 |
| 543845 | MCA1 | -1.197725814 | 0.001161497 |
| 543874 | GABA-TP2 | -2.133953795 | 9.58E-04 |
| 543899 | LOC543899 | -6.350359247 | 8.52E-15 |
| 543932 | LOC543932 | -1.734029801 | 4.31E-05 |
| 543937 | LOC543937 | -1.994631861 | 5.03E-06 |
| 543947 | MKK3 | -2.153616278 | 1.10E-05 |
| 543952 | Adi3 | -1.502314245 | 0.004297716 |
| 543957 | BiP/grp78 | -1.326924895 | 0.001574872 |
| 543971 | LOC543971 | -1.589343951 | 0.00321209 |
| 543984 | CaM6 | -1.125298938 | 0.005832385 |
| 543994 | LOX1.1 | -3.874624531 | 2.91E-05 |
| 543999 | Cellulase | -3.842297799 | 4.24E-11 |
| 544002 | Gad2 | -1.801495941 | 0.009159174 |
| 544008 | LoxC | -3.307363056 | 2.85E-08 |
| 544016 | PMEU1 | -3.263160753 | 2.82E-07 |
| 544067 | TPRP-F1 | -3.689994986 | 3.37E-04 |
| 544074 | ADH2 | -4.86460955 | 4.01E-05 |
| 544081 | LOC544081 | -2.640505675 | 9.72E-04 |
| 544122 | MFP1 | -1.33197685 | 0.001866268 |
| 544150 | DFR | -5.694245942 | 7.54E-11 |
| 544163 | NCED1 | -2.510497654 | 9.58E-07 |
| 544196 | Dem | -2.115012437 | 0.001338269 |
| 544211 | 20ox-2 | -4.731769845 | 0.003146907 |
| 544259 | FESOD | -1.809834812 | 0.003765775 |
| 544263 | LOC544263 | -1.450515888 | 4.90E-06 |
| 544274 | P18 | -2.783495994 | 0.006128371 |
| 544280 | IMP1 | -1.350441921 | 1.10E-04 |
| 544306 | AOC | -1.396920111 | 0.008091013 |
| 778227 | TUB | -2.526641481 | 1.10E-08 |
| 778232 | LOC778232 | -1.102231768 | 0.007968737 |
| 778246 | MTS1 | -2.442095558 | 0.005202154 |
| 778250 | FKBP12 | -1.244565347 | 0.005496332 |
| 778259 | LOC778259 | -1.755448817 | 8.17E-04 |
| 778262 | LOC778262 | -1.90753662 | 0.008428945 |
| 778272 | LOC778272 | -2.605497671 | 9.74E-16 |
| 778274 | VPE5 | -2.592559945 | 7.20E-10 |
| 778282 | H2B-1 | -2.72143829 | 5.13E-05 |
| 778288 | MDHAR | -1.22887387 | 0.008216657 |
| 778294 | CHS1 | -5.131063178 | 1.38E-12 |
| 778295 | CHS2 | -4.1139356 | 9.05E-13 |
| 778300 | MT3 | -2.197854607 | 0.007861848 |
| 778305 | MSH1 | -1.403608807 | 2.27E-04 |
| 778320 | LOC778320 | -1.377247464 | 0.003051528 |
| 778347 | CIP2a | -1.676784322 | 2.23E-04 |
| 778354 | H2B-2 | -2.531500203 | 2.05E-04 |

**Ta****ble S3.** **Differentially expressed genes related with plant hormone signaling pathways affected by *Cladosporium fulvum* treatment**

| **Gene ID** | **Gene Symbol** | **Seq Description** | **Log_2_ (*C.fulvum*/ Control)** | ***P*-value** |
| --- | --- | --- | --- | --- |
| 100037510 | SRK2C | SNF1-related kinase | 1.21 | 0.003101157 |
| 100191111 | LOC100191111 | PR1 protein | 6.51 | 2.22E-09 |
| 100736509 | ARF1 | Auxin response factor 1 | 1.17 | 0.00645183 |
| 100736531 | SERK3B | Somatic embryogenesis receptor kinase 3B | 0.87 | 0.002665399 |
| 101055547 | LOC101055547 | IAA14 | 0.84 | 0.003315808 |
| 101055555 | LOC101055555 | IAA35 | 1.80 | 0.009160947 |
| 101055583 | LOC101055583 | Small auxin-up protein 58 | 3.18 | 2.27E-12 |
| 101246226 | LOC101246226 | Auxin-responsive protein SAUR32 | 2.02 | 2.33E-04 |
| 101246590 | LOC101246590 | Ethylene-responsive transcription factor 1B | 4.73 | 1.18E-19 |
| 101246797 | LOC101246797 | Two-component response regulator ORR24-like | 1.00 | 9.06E-04 |
| 101246807 | LOC101246807 | Abscisic acid receptor PYL9 | 1.06 | 0.008230946 |
| 101247719 | LOC101247719 | Histidine kinase 3 | 0.94 | 0.004658903 |
| 101249950 | LOC101249950 | ETHYLENE INSENSITIVE 3-like 3 protein | 2.11 | 0.002604271 |
| 101250433 | LOC101250433 | Serine/threonine-protein kinase SAPK3 | 1.23 | 0.004081382 |
| 101250847 | LOC101250847 | Auxin-responsive protein SAUR50-like | 6.60 | 2.18E-07 |
| 101251509 | LOC101251509 | Serine/threonine-protein kinase BSK2 | 1.35 | 0.001163874 |
| 101251682 | GH3-5 | Putative indole-3-acetic acid-amido synthetase GH3.5 | 1.65 | 1.17E-05 |
| 101253234 | LOC101253234 | Auxin-responsive protein SAUR50-like | 2.23 | 0.00387351 |
| 101254707 | LOC101254707 | Probable xyloglucan endotransglucosylase/hydrolase protein 25 | 4.04 | 0.003732272 |
| 101255303 | IAA4 | Auxin-responsive protein IAA4 | 3.22 | 1.28E-10 |
| 101258886 | LOC101258886 | Abscisic acid receptor PYL3 | 1.07 | 0.003742465 |
| 101262109 | LOC101262109 | BRI1 kinase inhibitor 1 | 2.17 | 4.09E-05 |
| 101265243 | LOC101265243 | Auxin-responsive protein SAUR71 | 3.24 | 8.21E-04 |
| 101265524 | LOC101265524 | Protein phosphatase 2C 53 | 2.68 | 1.60E-09 |
| 101265927 | LOC101265927 | Probable xyloglucan endotransglucosylase/hydrolase protein 23 | 3.94 | 1.49E-09 |
| 101266334 | LOC101266334 | Two-component response regulator ARR1 | 1.52 | 1.74E-06 |
| 101267127 | LOC101267127 | Abscisic acid receptor PYR1 | 0.97 | 0.005559441 |
| 101268624 | GH3-1 | Jasmonic acid-amido synthetase JAR1-like | 1.34 | 7.05E-04 |
| 104645435 | LOC104645435 | Auxin-induced protein 15A-like | 1.98 | 5.94E-04 |
| 104645436 | LOC104645436 | Auxin-responsive protein SAUR21-like | 1.81 | 0.001693518 |
| 109118704 | LOC109118704 | Auxin-induced protein 15A-like | 2.31 | 4.33E-04 |
| 543542 | IAA7 | IAA7 protein | 1.88 | 3.94E-05 |
| 543600 | LOC543600 | Transcription factor TGA2.2 | 1.15 | 0.001692675 |
| 543712 | EREB | Ethylene responsive element binding protein | 5.26 | 5.03E-05 |
| 543939 | NPR1 | Regulatory protein NPR1 | 1.39 | 2.07E-04 |
| 544123 | PR1b1 | Pathogenesis-related leaf protein 6 | 7.54 | 1.38E-14 |
| 544185 | P4 | Pathogenesis-related protein P4 | 8.69 | 2.47E-06 |
| 544270 | NML2 | NIM1-like protein 2 | 1.94 | 1.83E-07 |
| 606712 | LOC606712 | Ethylene-responsive transcription factor 1 | 3.19 | 3.68E-05 |
| 100736448 | ARF5 | Auxin response factor 5 | -3.50 | 3.37E-11 |
| 100736477 | LAX2 | Auxin transporter-like protein 2 | -3.38 | 4.80E-14 |
| 100736541 | LAX5 | Auxin transporter-like protein 5 | -4.55 | 6.05E-04 |
| 100820704 | ABF4 | ABA responsive transcription factor | -1.10 | 0.006243886 |
| 101055548 | IAA15 | Auxin-regulated IAA15 | -1.83 | 0.002256359 |
| 101055549 | IAA19 | Auxin-responsive protein IAA19 | -1.92 | 0.004643909 |
| 101246001 | BOP2 | BLADE-ON-PETIOLE protein BOP2 | -3.21 | 0.006220984 |
| 101246270 | LOC101246270 | Auxin-responsive protein SAUR36-like | -2.09 | 6.36E-04 |
| 101247526 | LOC101247526 | Transcription factor TGA2.3-like | -1.47 | 1.58E-04 |
| 101248065 | LOC101248065 | Auxin-responsive protein SAUR71-like | -5.45 | 0.002862421 |
| 101248844 | LOC101248844 | Auxin-responsive protein SAUR50 | -5.53 | 8.48E-04 |
| 101249794 | LOC101249794 | Protein phosphatase 2C 51-like | -2.19 | 0.004197722 |
| 101251432 | LOC101251432 | Serine/threonine-protein kinase SRK2I | -2.84 | 3.23E-12 |
| 101255313 | LOC101255313 | Auxin-responsive protein SAUR71 | -1.74 | 0.006481308 |
| 101257321 | LOC101257321 | Auxin-responsive protein SAUR71-like | -3.85 | 4.31E-05 |
| 101260027 | LOC101260027 | Cyclin-D3-3 | -1.37 | 0.003576232 |
| 101265431 | LOC101265431 | Transcription factor TGA1 | -2.12 | 5.31E-04 |
| 101265854 | LOC101265854 | Pathogenesis-related leaf protein 4 | -2.38 | 1.94E-06 |
| 543544 | IAA17 | Auxin-responsive protein IAA17 | -3.02 | 6.02E-05 |
| 543722 | Cycd3c2 | D-type cyclin-2 | -3.95 | 6.91E-09 |
| 543748 | CycD3 | Cyclin D3.1 | -1.72 | 1.03E-04 |

**Table S4. Differentially expressed genes related with plant-pathogen interaction pathway by *Cladosporium fulvum* treatment**

| **Gene ID** | **Gene Symbol** | **Seq Description** | **Log_2_ (*C.fulvum*/ Control)** | ***P*-value** |
| --- | --- | --- | --- | --- |
| 100191111 | LOC100191111 | PR1 protein | 6.51 | 2.22E-09 |
| 100736531 | SERK3B | Somatic embryogenesis receptor kinase 3B | 0.87 | 0.002665399 |
| 101055527 | LOC101055527 | Hop-interacting protein THI080 | 1.45 | 9.70E-04 |
| 101244290 | LOC101244290 | Calmodulin-like protein 3 | 3.15 | 1.68E-09 |
| 101244669 | LOC101244669 | Cyclic nucleotide-gated ion channel 1-like | 4.73 | 1.11E-06 |
| 101245220 | CER6 | 3-ketoacyl-CoA synthase 6 | 0.74 | 0.007012538 |
| 101245298 | LOC101245298 | Calmodulin | 4.78 | 4.67E-06 |
| 101245539 | LOC101245539 | Probable calcium-binding protein CML44 | 1.24 | 0.00819727 |
| 101246110 | LOC101246110 | LRR receptor-like serine/threonine-protein kinase EFR | 2.20 | 1.97E-04 |
| 101248095 | LOC101248095 | FLAGELLIN-SENSING 3 protein | 4.76 | 1.80E-08 |
| 101251145 | Rcr3 | Cysteine protease | 4.05 | 4.63E-08 |
| 101252097 | LOC101252097 | WRKY transcription factor 1 | 2.12 | 5.29E-05 |
| 101255470 | LOC101255470 | Ethylene-inducing xylanase | 3.73 | 0.001123917 |
| 101256183 | LOC101256183 | Serine/threonine-protein kinase PBS1 | 0.81 | 0.003420758 |
| 101257476 | LOC101257476 | Calmodulin-like protein 8 | 4.16 | 2.38E-04 |
| 101257866 | LOC101257866 | Probable serine/threonine-protein kinase PBL7 | 4.75 | 1.48E-08 |
| 101260143 | HSP90 | Heat shock protein 90 | 7.66 | 4.12E-11 |
| 101260391 | LOC101260391 | Calcium-dependent protein kinase 18-like | 2.10 | 0.008481838 |
| 101260537 | LOC101260537 | Probable WRKY transcription factor 26 | 3.09 | 3.29E-10 |
| 101260980 | LOC101260980 | Probable LRR receptor-like serine/threonine-protein kinase At3g47570 | 3.41 | 0.003189953 |
| 101262858 | LOC101262858 | 3-ketoacyl-CoA synthase 20-like | 1.67 | 1.40E-05 |
| 101263667 | LOC101263667 | LRR receptor-like serine/threonine-protein kinase FLS2 | 2.62 | 1.82E-04 |
| 101264183 | LOC101264183 | Heat shock protein 83 | 4.65 | 4.23E-06 |
| 101264550 | LOC101264550 | Calcium-binding protein CP1 | 5.09 | 2.52E-09 |
| 101265816 | LOC101265816 | Caltractin | 1.06 | 0.007541617 |
| 101268230 | LOC101268230 | 3-ketoacyl-CoA synthase 1 | 2.67 | 1.06E-09 |
| 101268257 | LOC101268257 | 3-ketoacyl-CoA synthase 11-like | 4.88 | 0.004680944 |
| 112940015 | LOC112940015 | Calmodulin-like protein 1 | 1.81 | 4.66E-04 |
| 543571 | RBOH1 | NADPH oxidase | 1.68 | 0.003047459 |
| 543902 | Hsp90-1 | Molecular chaperone Hsp90-1 | 1.56 | 0.005490198 |
| 544041 | PTI4 | DNA-binding protein Pti4 | 2.87 | 2.42E-04 |
| 544123 | PR1b1 | Pathogenesis-related leaf protein 6 | 7.54 | 1.38E-14 |
| 544185 | P4 | Pathogenesis-related protein P4 | 8.69 | 2.47E-06 |
| 101244112 | LOC101244112 | Protein CNGC15b-like | -2.87 | 1.55E-04 |
| 101244728 | LOC101244728 | Caltractin | -3.98 | 4.44E-06 |
| 101246133 | LOC101246133 | Calcium-dependent protein kinase 29 | -2.37 | 1.23E-08 |
| 101250418 | LOC101250418 | Calcium-dependent protein kinase 24 | -1.81 | 0.006829089 |
| 101255379 | LOC101255379 | Calcium-dependent protein kinase 17-like | -3.60 | 4.70E-08 |
| 101256200 | LOC101256200 | Calcium-dependent protein kinase 1 | -2.30 | 2.84E-12 |
| 101262214 | LOC101262214 | 3-ketoacyl-CoA synthase 20 | -1.96 | 7.30E-04 |
| 101265854 | LOC101265854 | Pathogenesis-related leaf protein 4 | -2.38 | 1.94E-06 |
| 543947 | MKK3 | MAPKK | -2.15 | 1.10E-05 |
| 543984 | CaM6 | Calmodulin 6 | -1.13 | 0.005832385 |
| 778272 | LOC778272 | Putative nitric oxide synthase | -2.61 | 9.74E-16 |

**Table S5.** **Differentially expressed genes involved in phenylpropanoid biosynthesis pathway by *Cladosporium fulvum* treatment**

| **Gene ID** | **Gene Symbol** | **Seq Description** | **Log_2_ (*C.fulvum*/ Control)** | ***P*-value** |
| --- | --- | --- | --- | --- |
| 101261193 | LOC101261193 | Anthocyanidin 3-O-glucosyltransferase 5 | 5.80 | 6.61E-09 |
| 101261765 | LOC101261765 | Cytochrome P450 98A3 | 5.73 | 1.85E-07 |
| 101244961 | LOC101244961 | Agmatine coumaroyltransferase-2 | 5.36 | 4.94E-11 |
| 101255851 | LOC101255851 | Acylsugar acyltransferase 3-like | 5.21 | 0.004820866 |
| 101259903 | LOC101259903 | 8-hydroxygeraniol dehydrogenase | 5.03 | 6.01E-09 |
| 101253503 | LOC101253503 | Vinorine synthase-like | 4.53 | 1.22E-11 |
| 101250635 | LOC101250635 | Probable cinnamyl alcohol dehydrogenase 1 | 2.48 | 7.33E-20 |
| 544103 | Twi1 | Scopoletin glucosyltransferase | 1.93 | 0.007606809 |
| 101265187 | LOC101265187 | Caffeoyl-CoA O-methyltransferase-like | 1.66 | 0.006712243 |
| 101260278 | LOC101260278 | Caffeoyl-CoA O-methyltransferase 6 | 1.55 | 0.002367959 |
| 101266953 | LOC101266953 | Vinorine synthase | 1.51 | 2.96E-04 |
| 101266599 | LOC101266599 | Feruloyl CoA ortho-hydroxylase 2 | 1.14 | 0.004924102 |
| 101260057 | LOC101260057 | Beta-glucosidase BoGH3B | 0.97 | 2.56E-04 |
| 101247788 | LOC101247788 | Aldehyde dehydrogenase family 2 member C4 | -1.04 | 1.42E-04 |
| 101248047 | LOC101248047 | Beta-glucosidase 11 | -1.39 | 1.18E-04 |
| 101266300 | LOC101266300 | Feruloyl CoA ortho-hydroxylase 2 | -2.60 | 1.26E-06 |
| 101261963 | CTOMT1 | Catechol-O-methyltransferase 1 | -2.84 | 5.33E-08 |
| 543698 | LOC543698 | Beta-mannosidase | -2.88 | 1.66E-07 |
| 101245999 | LOC101245999 | Probable cinnamyl alcohol dehydrogenase 6 | -4.07 | 4.69E-09 |
| 101247513 | LOC101247513 | Beta-glucosidase BoGH3B-like | -4.08 | 3.25E-17 |
| 101252173 | LOC101252173 | Probable caffeoyl-CoA O-methyltransferase At4g26220 | -4.79 | 8.51E-07 |
| 101255734 | AnthOMT | Flavonoid 3,5-methyltransferase | -4.86 | 6.02E-07 |
| 100191129 | LOC100191129 | Anthocyanin acyltransferase | -6.23 | 5.78E-12 |
| 101246223 | LOC101246223 | Beta-glucosidase 44-like | -6.32 | 2.28E-05 |
| 101255272 | LOC101255272 | Beta-glucosidase 46 | -7.50 | 1.21E-07 |
